# Common, low-frequency, and rare genetic variants associated with lipoprotein subclasses and triglyceride measures in Finnish men from the METSIM study

**DOI:** 10.1371/journal.pgen.1007079

**Published:** 2017-10-30

**Authors:** James P. Davis, Jeroen R. Huyghe, Adam E. Locke, Anne U. Jackson, Xueling Sim, Heather M. Stringham, Tanya M. Teslovich, Ryan P. Welch, Christian Fuchsberger, Narisu Narisu, Peter S. Chines, Antti J. Kangas, Pasi Soininen, Mika Ala-Korpela, Johanna Kuusisto, Francis S. Collins, Markku Laakso, Michael Boehnke, Karen L. Mohlke

**Affiliations:** 1 Department of Genetics, University of North Carolina at Chapel Hill, Chapel Hill, NC, United States of America; 2 Department of Biostatistics and Center for Statistical Genetics, University of Michigan, Ann Arbor, MI, United States of America; 3 National Human Genome Research Institute, National Institutes of Health, Bethesda, MD, United States of America; 4 Computational Medicine, Faculty of Medicine, University of Oulu and Biocenter Oulu, Oulu, Finland; 5 NMR Metabolomics Laboratory, School of Pharmacy, University of Eastern Finland, Kuopio, Finland; 6 Population Health Science, Bristol Medical School, University of Bristol and Medical Research Council Integrative Epidemiology Unit at the University of Bristol, Bristol, United Kingdom; 7 Systems Epidemiology, Baker Heart and Diabetes Institute, Melbourne, Victoria, Australia; 8 Department of Epidemiology and Preventive Medicine, School of Public Health and Preventive Medicine, Faculty of Medicine, Nursing and Health Sciences, The Alfred Hospital, Monash University, Melbourne, Victoria, Australia; 9 Institute of Clinical Medicine, Internal Medicine, University of Eastern Finland and Kuopio University Hospital, Kuopio, Finland; Icahn School of Medicine at Mount Sinai, UNITED STATES

## Abstract

Lipid and lipoprotein subclasses are associated with metabolic and cardiovascular diseases, yet the genetic contributions to variability in subclass traits are not fully understood. We conducted single-variant and gene-based association tests between 15.1M variants from genome-wide and exome array and imputed genotypes and 72 lipid and lipoprotein traits in 8,372 Finns. After accounting for 885 variants at 157 previously identified lipid loci, we identified five novel signals near established loci at *HIF3A*, *ADAMTS3*, *PLTP*, *LCAT*, and *LIPG*. Four of the signals were identified with a low-frequency (0.005<minor allele frequency [MAF]<0.05) or rare (MAF<0.005) variant, including Arg123His in *LCAT*. Gene-based associations (*P*<10^−10^) support a role for coding variants in *LIPC* and *LIPG* with lipoprotein subclass traits. 30 established lipid-associated loci had a stronger association for a subclass trait than any conventional trait. These novel association signals provide further insight into the molecular basis of dyslipidemia and the etiology of metabolic disorders.

## Author summary

Lipid and lipoproteins are heritable traits that differ in content and size and are correlated with coronary heart disease and mortality. To identify genetic variants associated with different subclasses of lipoproteins, we conducted a genome-wide association study of 8,372 Finnish men. We curated a dataset of all genetic variants known to be associated with lipid or lipoprotein subclasses and used these data to conduct rigorous analyses to identify new associations in the same gene region or new ones. We identified five new signals at established lipid-associated loci revealing possible complex regulatory mechanisms underlying the signals. Using the contribution of rare coding variants predicted to be protein truncating or missense, we uncovered novel associations for a set of variants at *LIPC* and *LIPG* with HDL subclasses. Investigating the genetic association of lipoprotein subclass traits may help lead to a better understanding of the etiology of cardio-metabolic diseases, and provide novel therapeutic targets.

## Introduction

Genome-wide association studies (GWAS) have identified hundreds of common (MAF>0.05) variants associated with conventional lipid and lipoprotein traits: high-density lipoprotein cholesterol (HDL-C), low-density lipoprotein cholesterol (LDL-C), total cholesterol (TC), and triglycerides (TG)[1–4]. While some low-frequency (0.005<MAF≤0.05) and rare variants (MAF≤0.005) have been associated with lipid and lipoprotein traits, additional loci remain to be identified[2,5,6]. High-throughput proton nuclear magnetic resonance (NMR)-based measurements of lipid and lipoprotein subclasses provide a more comprehensive view of particle size and composition than conventional blood lipid profile measurements[7], and these expanded sets of traits have been associated with metabolic and cardiovascular diseases[8–10]. For example, HDL subclasses are differentially associated with incidence of coronary heart disease, and VLDL particle size is negatively associated with mortality[11,12].

Previous association studies for lipid traits have identified several genomic regions of <1 Mb that contain more than one association signal for which the lead variants are not in strong linkage disequilibrium (LD) (*r*^*2*^<0.8)[2,3,13]. Fine-mapping with higher density variants and conditional analyses can determine which signals are distinct (remain significant after conditional analysis) and which are independent, which we define here as *r*^*2*^<0.01. For example, Teslovich *et al*. used conditional analysis at 95 lipid loci to identify 26 loci that harbor at least two distinct association signals[3]. Association signals at the same locus can be population-specific or shared across populations, with potentially different effect sizes and/or lead variants[13]. Multiple association signals at a locus may indicate allelic heterogeneity in gene function or regulation or that more than one gene at the locus affects the trait[14]. Furthermore, identifying and accounting for additional independent association signals increases the variance in traits that can be explained by genetic loci[15,16].

In this study, we performed genome-wide single-variant and gene-based association analyses of 68 NMR lipid and lipoprotein subclass traits and four conventional traits (TC, TG, HDL-C, and LDL-C) in 8,372 non-diabetic Finnish men from the METabolic Syndrome In Men (METSIM) study[17]. To identify novel associations, we performed analyses with and without conditioning on lipid-associated variants at loci previously described in array- and sequence-based GWAs. We identified the most strongly associated lipid and lipoprotein subclass traits at established loci for conventional lipid and lipoprotein traits. Since several subclasses are associated with cardiovascular and metabolic diseases, identifying the variants that influence these traits is the first step to develop novel clinical treatments. These expanded association results have the potential to lead to advances in determining the etiological role of the variants and genes in cardiovascular and metabolic disease.

## Results

### Genome-wide association study

To identify genetic variants associated with the 72 lipid and lipoprotein traits, we analyzed 15.1M genotyped and imputed variants in 8,372 non-diabetic Finnish men (S1 Table, S1 Fig). Each trait was adjusted for age, age^2^, lipid-lowering medication use, and smoking status. Inverse normalized trait residuals were tested for association with each variant assuming additive allelic effects using a linear mixed model to account for relatedness among study participants[18]. Many of the traits are highly correlated with each other, with 104 trait-pair comparisons having a pairwise Pearson correlation greater than 0.98 (S2 Fig). We used a genome-wide significance threshold of *P*≤5×10^−8^, consistent with previous association studies of this scale and high trait correlation[19]. We note where associations meet a conservative experiment-wide Bonferonni-corrected *P*-value (*P*≤4.6×10^−11^). We identified 32,524 variant-trait associations (*P*_discovery_<5×10^−8^) for the 72 lipid and lipoprotein traits (S3 Fig). 30,348 (93%) of the 32,524 associations were for one of the 68 subclass traits and 2,176 (7%) for one of the four conventional lipid traits (TC, TG, HDL-C, LDL-C). More than half the associations were with the VLDL- (38%) or HDL-subclass (29%) traits. 3,784 unique variants comprise the total 32,524 trait-variant associations (S2 Table). 73% (2,780) of the 3,784 variants had a greater association with one of the 68 subclass traits, and 27% (1,004) were more highly associated with at least one of the four conventional traits (S2 Table). These variants cluster into 42 loci that were associated with at least one of the 72 traits (S1 Table). For example, at the well-characterized *APOA5* locus on chromosome 11, rs964184 was significantly associated (*P*_discovery_<5×10^−8^) with 43 of the 72 lipid and lipoprotein traits. At *CETP*, rs12446515 was significantly associated with 34 of the 72 traits. For such loci, the high correlation between the traits obscures identification of a causal trait underlying the signal.

### Conditional analyses to identify novel loci and signals

To identify novel associations not reported previously for any conventional or subclass lipid or lipoprotein trait, we identified and curated a list of previously known associated variants to use in genome-wide conditional analyses (Methods). We identified 1,714 variants (S3 Table) that we clustered based on stringent LD (*r*^*2*^>0.95) into 885 representative variants (S4 Table). After genome-wide conditional analysis using these 885 variants, we defined novel association signals using a significance threshold of *P*_*conditional*_<5×10^−8^ (S5 Table). Consistent with highly correlated traits, we observed that most of the associated variants were associated with multiple correlated traits. We considered variants located within 1 Mb of an established lipid or lipoprotein signal to be an additional signal in the region, and we define a locus as the region 1 Mb up- and downstream of a signal. We considered additional signals independent if the signal was not in LD (*r*^*2*^<0.01) with known lipid/lipoprotein signals, and remained significant (*P*_*single*_<5×10^−8^) after single-variant conditional analyses. Associated variants with MAF<0.01 were validated by direct genotyping or sequencing (see Methods). Using this genome-wide conditional approach, we identified five novel signals near established lipid and lipoprotein loci (Table 1).

**Table 1:** Newly identified signals associated with lipoprotein subclasses and triglyceride measures.

*Variant*	Lead trait*	*Chr*†	*Position (hg19)*	Locus**	*Nearest GWAS gene*	*Distance to GWAS gene*	*Ref/Alt*	*MAF¶*	*MAC¶¶*	*Beta§*	*P*_*discovery*_*‡*	*P*_*conditional*_*‡‡*	*Variance explained*
**Common variants**													
rs73059724	Phospholipids in small VLDL	19	46,796,767	*HIF3A*	*HIF3A*	3.5 kb	T/C	0.091	1,521	-0.14	3.8×10^−7^	1.4×10^−8^	0.004
**Low-frequency variants**													
rs187918276	Conc.†† of small LDL particles	4	74,033,564	*ALB*	*ADAMTS3*	887 kb	G/C	0.017	278	0.60	6.3×10^−22^§§	3.2×10^−11^§§	0.011
rs184392658	Conc.†† of large HDL particles	20	44,067,565	*SYS1*	*PLTP*	460 kb	T/C	0.008	137	0.45	2.3×10^−7^	2.5×10^−9^	0.003
**Rare variants**													
rs199717050	HDL cholesterol	16	67,976,823	*LCAT*	*LCAT*	*—*	C/T	0.005	79	-0.72	5.9×10^−10^	2.5×10^−12^§§	0.005
rs538509310	Phospholipids in medium HDL	18	47,343,410	*LIPG-ACAA2*	*LIPG*	255 kb	T/A	0.004	74	0.72	1.7×10^−9^	3.2×10^−10^	0.004

*Lead trait is the lipoprotein subclass or triglyceride measure with lowest P-value across the 72 traits.

**Locus is labeled with the most biologically relevant gene within 1 Mb of lead variant.

§Beta is reported for the alternate allele, and is in standard deviation units.

‡P_discovery_, is the unconditional p-value for the lead variant and trait.

‡‡P_conditional_, is the p-value for the lead variant after conditioning on 885 known lipid GWAS variants (S4 Table).

†Chr, chromosome.

¶MAF, minor allele frequency.

¶¶MAC, minor allele count.

††Conc, concentration.

§§ Meets Bonferroni-corrected P-value (*P*≤4.6×10^−11^)

### One novel common variant signal at *HIF3A*

Common variant rs73059724 (MAF = 0.09), associated with decreased (β = –0.14) concentrations of phospholipids in small VLDL, is located 3.5 kb upstream of *HIF3A* (hypoxia inducible factor 3, alpha subunit) and 1.4 Mb from *APOE* (S4A and S5A Figs). Additionally, this signal is associated with decreased VLDL subclass traits (S6 Fig). This signal achieved significance after conditioning on known lipid GWAS variants (*P*_*discovery*_ = 3.8×10^−7^, *P*_*conditional*_ *=* 1.4×10^−8^) (Table 1, S6 Table). When adjusted for total triglycerides, the strength of the association of rs73059724 with phospholipids in small VLDL was reduced (P = 3.6×10^−2^, S7 Table). This signal is located in a gene-dense region on chromosome 19 that includes 10 previously reported lipoprotein-associated variants within 1 Mb of the index variant (S6 Table)[20]; none of these variants exhibited LD (*r*^*2*^>0.02) with rs73059724. Further analysis of the *APOE* locus with additional samples may be necessary to elucidate the haplotype relationships between these signals. Twenty-nine proxy variants in LD (*r*^*2*^>0.7) with rs73059724 span a 25-kb region including the promoter and intron 1 of *HIF3A*, and five of these variants overlap ≥5 liver and adipose regulatory element (histone marks of transcriptional regulation and open chromatin) datasets (S8 Table). Hyper-methylation at *HIF3A* is associated with increased adiposity and BMI in Asian infants and children[21,22]. HIF3A is a known negative regulator of *HIF1A* (hypoxia inducible factor 1, alpha subunit)[23], which has been shown to regulate the cellular uptake of cholesterol esters and VLDL by creating hypoxic conditions[24]. One or more of the associated variants may affect *HIF3A* transcription or other genes in the region, leading to fewer phospholipids in small VLDL particles.

### Two novel low-frequency variant signals at *ALB* and *SYS1*

We identified two new signals with low-frequency variants located near *ALB* and *SYS1* (Table 1, S4B and S5B Figs). At the *ALB* locus, the low-frequency allele of rs187918276 (MAF = 0.017) located in intron 1 of *ANKRD17* was associated with increased (β = 0.60) concentration of small LDL particles (*P*_*discovery*_ *=* 6.3×10^−22^, *P*_*conditional*_ *=* 3.2×10^−11^) and 26 additional traits, including increased TC, LDL-C, esterified cholesterol, free cholesterol, and IDL/LDL/VLDL subclasses (S6 Fig). When adjusted for total cholesterol, the strength of the association of rs187918276 with small LDL particles was reduced (*P* = 9.1×10^−7^, S7 Table). Variants in LD (*r*^*2*^>0.7, METSIM) with this variant span >1.2 Mb (S5B Fig, S8 Table), consistent with long haplotypes previously described in Finns[25]. The 885 variants used for the conditional analysis included established TC-associated signals at rs60873279 and rs182616603, located 337 kb and 1 Mb away; these variants exhibited low (*r*^*2*^<0.01) and moderate (*r*^*2*^ = 0.39) pairwise LD with rs187918276 (S6 Table). When conditioned on rs182616603, the association with rs187918276 was reduced but still highly significant (*P*_*single*_ *=* 5×10^−15^), suggesting the signals are distinct. An additional variant at this locus, rs115136538, was reported previously to be associated with albumin levels[5]. rs115136538 is located 710 kb away from and is not in LD with rs187918276 (*r*^*2*^<0.01 in METSIM), and the association of rs187918276 with small LDL particles was essentially unchanged when conditioned on rs115136538 (S6 Table). Taken together, the *ALB* region contains three distinct signals for lipid traits (rs60873279, rs182616603, and now rs187918276).

*ALB* encodes albumin, which is responsible for shuttling cholesterol in the blood to the lipoprotein particle acceptors; deletion of *Alb* in mice led to a hyperlipidemic condition[26,27]. One of the 12 variants in LD (*r*^*2*^>0.7) with rs187918276, chr4:74265673, is located 4.3 kb upstream of the *ALB* transcription start site (TSS), and is the only variant that overlapped any epigenomic marks of transcriptional regulation from the adipose, blood, and liver datasets (S8 Table). This variant may mediate a regulatory effect on *ALB* to increase the plasma concentration of small LDL particles, or another of the candidate variants spanning 1.2 Mb may act on this or another nearby gene.

In an intergenic region downstream of *PIGT*, we identified the low-frequency allele of lead variant rs184392658 (MAF = 0.008) associated with the increased (β = 0.45) concentration of large HDL particles (*P*_*discovery*_ *=* 2.3×10^−7^, *P*_*conditional*_ *=* 2.5×10^−9^, Table 1, S4C Fig and S5C Fig). When adjusted for HDL-C, the association of rs184392658 with large HDL particles was reduced (*P* = 4.1×10^−5^, S7 Table). Two previously established lipid-associated variants are located within 1 Mb of rs184392658: rs1800961 near *HNF4A* and rs6065904 near *PLTP*. rs184392658 was not in LD (*r*^*2*^<0.015) with either of these established variants, and conditioning on the individual known variants did not substantially change the association signal (all *P*_*single*_<3.7×10^−6^, S6 Table). Thus, rs184392658 represents a new distinct signal in this region. Of six variants in high LD (*r*^*2*^>0.7) with lead variant rs184392658, only rs149985455 overlaps multiple epigenomic marks of transcription regulation from liver, blood, and adipose tissue datasets (S8 Table). This variant is located 2.2 kb upstream from *SYS1* (Sys1 Golgi trafficking protein), which may have a role in lipid metabolism through an interaction with GTPases[28]. SYS1 targets ARFRP1 (ADP-ribosylation factor-related protein 1) and forms a complex in the Golgi membrane[29]; deletion of *Arfrp1* in mouse adipocytes led to lipodystrophy caused by failure in lipid droplet formation[30]. rs149985455 may mediate a regulatory effect on *SYS1* to increase the plasma concentration of large HDL particles, or another of the candidate variants spanning >500 kb may act on this or another nearby gene.

### Two novel rare variant signals at *LCAT* and *LIPG*

We identified additional novel independent signals with rare variants near *LCAT* and *LIPG* (Table 1). The rare allele (MAF = 0.005) of the missense variant rs199717050 (Arg123His) in exon 3 of *LCAT* (lecithin-cholesterol acyltransferase) was associated with decreased (β = –0.72) HDL-C levels (*P*_*discovery*_ *=* 5.9×10^−10^, *P*_*conditional*_ *=* 2.5×10^−12^, Table 1, S4D Fig and S5D Fig). This signal was not significantly associated with any of the HDL subclass traits or other traits from this study (S6 Fig). The association of rs199717050 with HDL-C was nominally reduced (*P* = 2.9×10^−8^) when adjusted for total cholesterol (S7 Table). Six variants at this locus, within 1 Mb of rs199717050, were reported previously to be associated with HDL-C[2,4] (S6 Table). However, these six variants all show low pairwise LD with rs199717050 (*r*^*2*^<0.01), and single-variant conditional analyses using any one of the six variants did not substantially change the association of rs199717050 with HDL-C (*P*_*single*_ ≤1.9×10^−9^, S6 Table). rs199717050 may be nearly specific to Finns; the Exome Aggregation Consortium (ExAC) database shows a total allele count of 16: fifteen in Finns and one in a non-European population. LCAT is responsible for cholesterol esterification for eventual transfer into the lipoprotein core, and facilitates the transport of cholesterol into the liver[31]. rs199717050 is predicted to be deleterious (SIFT, 0.02) or possibly damaging (PolyPhen, 0.55)[32], consistent with a plausible functional effect on LCAT to decrease levels of HDL-C.

**Fig 1:**
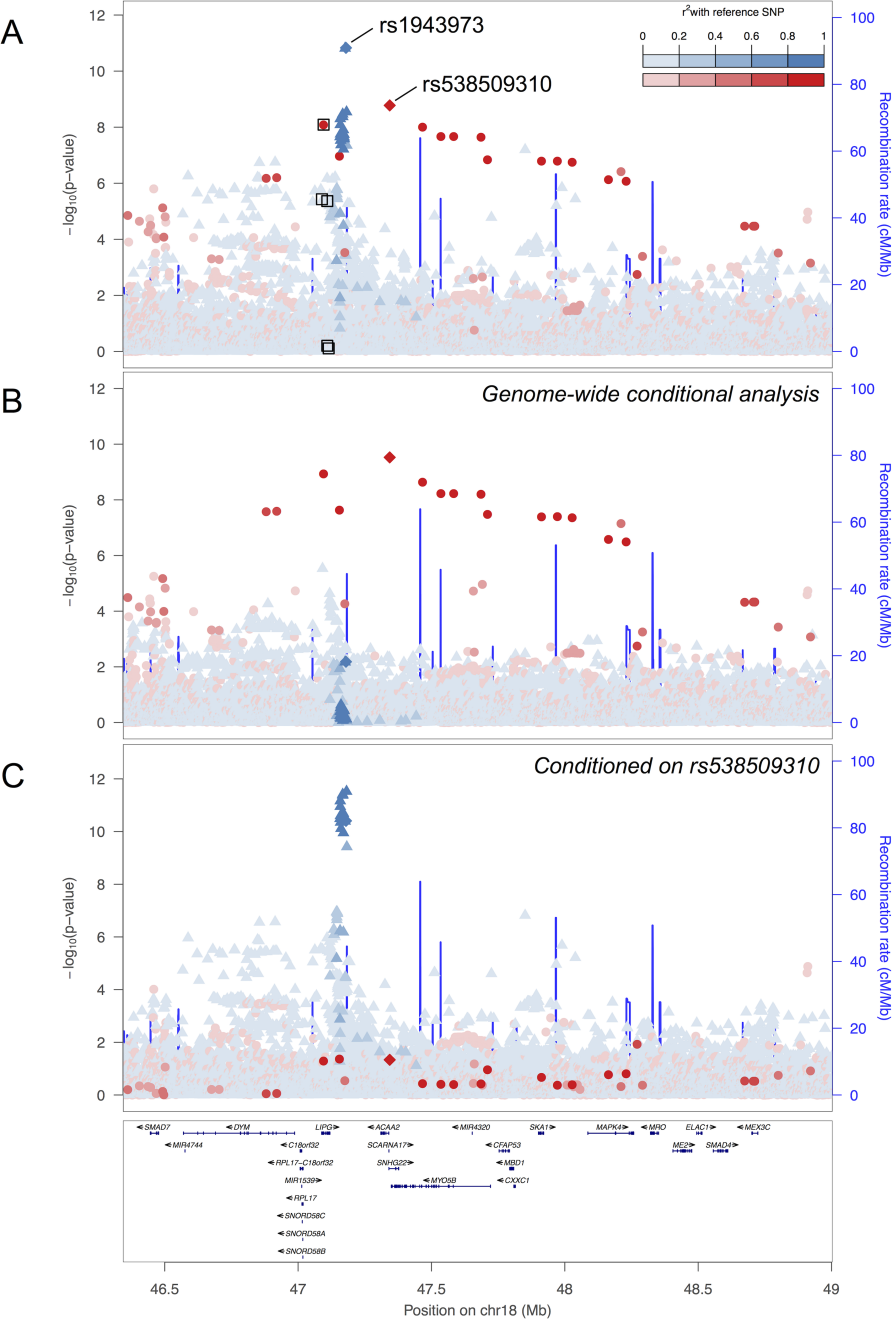
Novel independent signal at *LIPG*. Association with phospholipids in medium HDL at the *LIPG* locus. The colors and shapes distinguish the association signals and are based on the LD (r^2^) in METSIM samples between each variant and a reference variant, rs538509310 or rs1943973, represented in red and blue, respectively. X-axis, genomic (GRCh37/hg19) position in Mb. Left y-axis, p- value of variant-trait association in–log_10_. Right y-axis, local estimates of genomic recombination rate in cM/Mb, represented by blue lines. (A) Unconditional association with phospholipids in medium HDL. Black squares indicate the five coding variants (rs200435657, rs201922257, rs142545730, rs138438163, and rs77960347) used in the *LIPG* gene-based association tests. (B) Association with phospholipids in medium HDL after genome-wide conditional analysis of known lipid-associated variants (n = 885). (C) Association with phospholipids in medium HDL after conditioning on rs538509310. The association plots for four additional signals at *HIF3A*, *ALB*, *SYS1*, and *LCAT* are provided in S4 Fig and S5 Fig.

Another novel signal was located at the well-established HDL-C-associated *LIPG* locus (Fig 1)[33]. The rare allele (MAF = 0.004) of lead variant rs538509310 is located 3.6 kb upstream from *ACAA2*, and was most strongly associated with increased (β = 0.72) levels of phospholipids in medium-size HDL (*P*_*discovery*_ *=* 1.7×10^−9^, *P*_*conditional*_ *=* 3.2×10^−10^, Table 1, Fig 1A and 1B). This signal was also significantly associated with increased levels of four other HDL subclass traits and apolipoprotein A-I (S6 Fig). When adjusted for HDL-C, the association of rs538509310 with phospholipids in medium-size HDL was reduced (*P* = 4.5×10^−5^) (S7 Table). rs538509310 is in near complete LD (*r*^*2*^ = 0.98) with rs201922257, which encodes a missense substitution (Ala172Val) in exon 4 of *LIPG*. At least four previously described HDL-C variant association signals are located within 1 Mb of this variant, including rs74558535 (*P* = 2×10^−10^), rs10438978 (*P* = 7.7×10^−36^), rs77960347 (*P* = 3.6×10^−11^), and rs2156552 (*P* = 2×10^−12^). The new signal is not in LD (*r*^*2*^<0.043) with the previously described variants and remained significant after single-variant conditional analyses (S6 Table, Fig 1C). *LIPG* encodes endothelial lipase (EL), which catalyzes HDL phospholipids and aids in the sequestration of HDL from circulation, and is expressed in several tissues and organs including the liver[34–36]. The association with phospholipids in medium-size HDL is consistent with the known phospholipase of EL[37]. Several variants in *LIPG* have been shown to decrease endothelial lipase levels and increase HDL-C[38]. Based on the direction of effect in these previous studies, missense variant (A172V) may decrease function of LIPG, leading to increased phospholipids in medium-size HDL and other HDL subclasses.

### Gene-based tests of association

**Fig 2:**
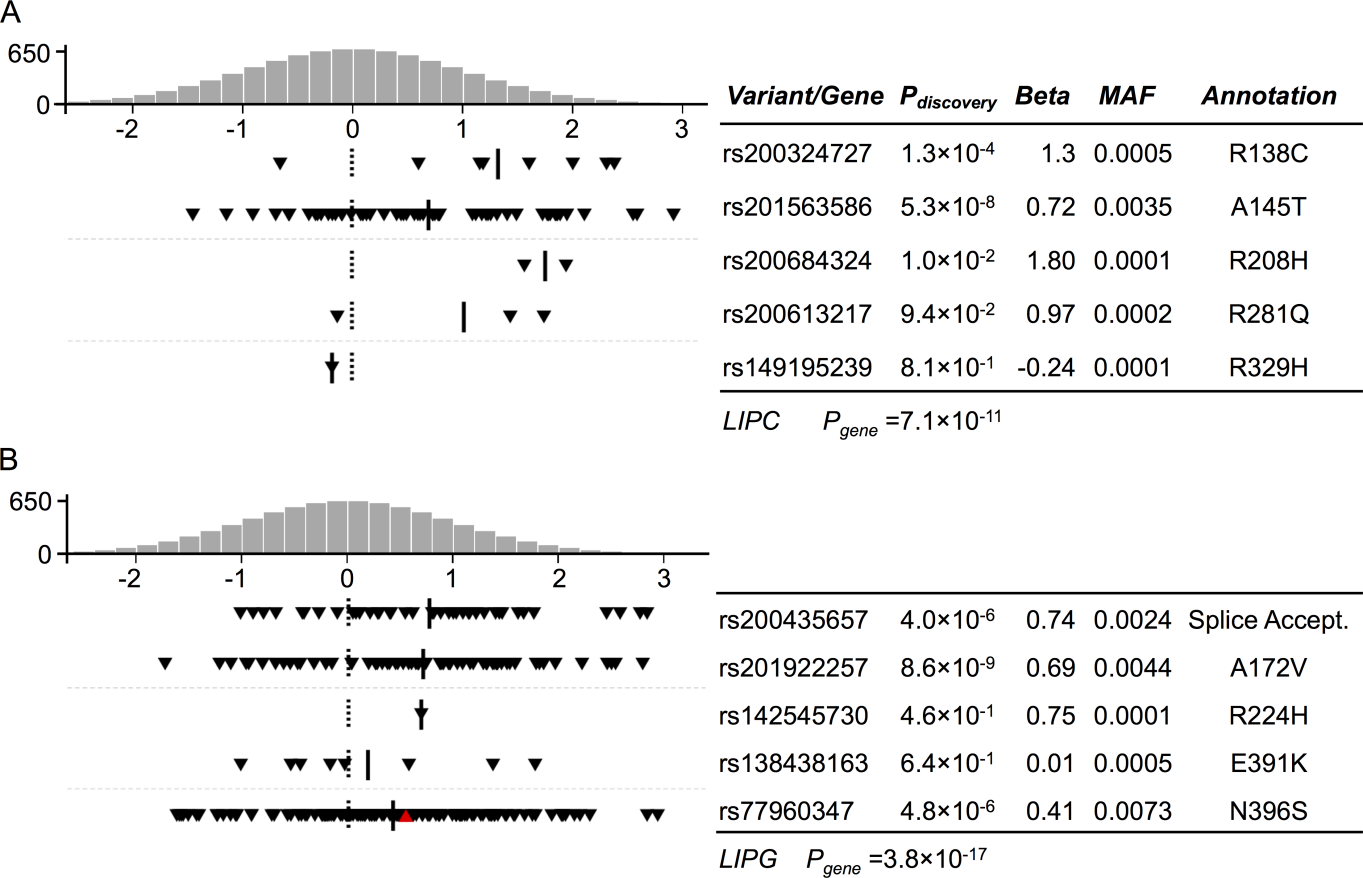
Gene-based tests of association with HDL subclass traits for *LIPC* and *LIPG*. The distribution of the inverse normalized residuals of the trait values for all individuals (histogram) compared to individuals carrying variants included in the gene-based tests of association (triangles) (A) at *LIPC* with triglycerides in very large HDL and (B) at *LIPG* with phospholipids in medium HDL. The histograms indicate counts of individuals per trait bin in the METSIM study, and the dashed gray line below the histograms indicates the mean trait level. The rows of black and red triangles represent individuals that are heterozygous and homozygous, respectively for each variant indicated, and the solid black lines indicate the mean trait level for variant carriers. *P*_*discovery*_, p-value for the individual variant-trait association; *P*_*gene*_, p-value for the gene-based test of association; Annotation, functional annotation of the variants; Splice accept., splice acceptor variant. Figure created with VARV (https://github.com/shramdas/varv).

To test the association between lipid and lipoprotein subclasses and sets of coding variants within a gene, we performed gene-based tests of association using SKAT-O with four variant masks (Methods) based on the predicted function of the coding variants. Sets of variants in *LIPC* (*P*_*gene*_ = 7.1×10^−11^) and *LIPG* (*P*_*gene*_ *=* 3.8×10^−17^) were associated with lipid and lipoprotein subclasses using the gene-based method; these results remained significant after adjusting for nearby noncoding signals (*LIPC P*<1.3×10^−10^ and *LIPG P*<1.2×10^−17^) (Fig 2, S9 Table).

At *LIPC*, the set of five rare missense variants, R138C, A145T, R208H, R281Q, and R329H, showed the strongest association using the protein truncating variant (PTV)+missense mask with triglycerides in very large HDL (Fig 2A, *P*_*gene*_ = 7.1×10^−11^). Of the five single-variant tests of association with triglycerides in very large HDL, A145T was individually the most significant (*P*_*discovery*_ *=* 5.3×10^−8^). Four of the variants (R138C, A145T, R208H, and R281Q) showed higher trait levels (β = 0.72 to 1.8) and were predicted to be deleterious by Variant Effect Predictor (VEP), while R329H, observed in one individual, showed a modestly lower trait level (β = –0.24) and was predicted to be benign[32]. While rare, A145T had 1.7-fold higher allele frequency in Finns (0.003%) than other populations[39]. Three of the variants, A145T, R138C, and R208H, were associated with increased HDL-C in a previous gene-based association study, consistent with our results[40]. Among the other variants, the relatively high trait values for R281Q suggest that it may also increase HDL-C. Based on previous data that decreased *LIPC* expression can result in increased large HDL levels[41], the rare alleles may lead to reduced LIPC function. Consistent with the gene-based test, deficiency in hepatic lipase activity resulted in increased concentration of triglycerides in plasma HDL[42].

At *LIPG*, the PTV+missense mask showed five variants with the strongest association with phospholipids in medium-size HDL (Fig 2B, *P*_*gene*_ *=* 3.8×10^−17^). Of the five single-variant tests, a rare missense (A172V) variant rs201922257 was the only one significantly associated (*P*_*discovery*_ *=* 8.6×10^−9^) with the subclass trait, and in three of four transcripts the amino acid substitution is predicted by VEP to be ‘deleterious’ and ‘probably damaging’ in most of the transcripts (Fig 2B). This variant is in LD (*r*^*2*^ = 0.98) with the non-coding index variant rs538509310 for phospholipids in medium HDL (Table 1, Fig 1A). The other associated variants may also affect LIPG function despite less-significant *P*-values. A splice variant rs200435657 (MAF = 0.0035, *P*_*discovery*_ *=* 4.0×10^−6^) is located at the 3’ end of intron 1; this variant has only been observed once (1/121,029; 0.0008%) in non-Finnish ExAC samples. Based on position, this splice variant is predicted to cause skipping of exon 2, which would lead to four aberrantly coded amino acids and a stop codon in exon 3. The remaining missense variants are predicted by VEP to be deleterious except for E391K. N396S and E391K have been reported previously to be associated with increased HDL-C levels[43–45]. However, our data suggest that all five variants increase phospholipids in medium HDL (β = 0.01 to 0.75) (Fig 2B). Together, the gene-based tests suggest that additional rare variants may influence *LIPG* function and HDL-C subclass levels.

### Lipid and lipoprotein associations at known lipid and coronary artery disease loci

We next asked whether any of 157 previously known loci associated with one or more of the four conventional lipid and lipoprotein traits exhibited stronger evidence of association with one of the lipid or lipoprotein subclass traits. Among the 157 loci associated (*P*<5×10^−8^) here with at least one subclass trait, 30 showed stronger association with a subclass trait than any conventional trait (Table 2, S7 Fig). For example, at *PLTP* (phospholipid transfer protein), rs4812975 was much more strongly associated with HDL diameter (*P*_*subclass*_ = 1.4×10^−15^) than with HDL-C (*P*_*conventional*_ = 2.6×10^−3^), consistent with PLTP mediating the net transfer of phospholipids between lipoproteins and uptake of phospholipids into the HDL-C core[46]. In addition, at *ANGPTL3* (angiopoietin-like 3), *ANGPTL4* (angiopoietin-like 4), and *LPL* (lipoprotein lipase), the variants were all more strongly associated with VLDL subclass traits than with the conventional traits (Table 2), consistent with studies showing that mouse *Angptl3* knockout and *Angptl4* overexpression may act via *Lpl* to decrease or increase VLDL, respectively[47,48].

**Table 2:** Comparison of METSIM association data between conventional lipid traits and subclass traits at established loci.

*Variant*	*Chr*:*Position*	*Locus*	Standard Trait*	*P*_conventional_	Subclass Trait**	*P*_subclass_	*Log Difference§*
rs261291	15:58680178	*LIPC*	HDL	1.2×10^−17^	Triglycerides in very large HDL	2.0×10^−85^	67.8
rs1065853	19:45413233	*APOE*	LDL	5.1×10^−50^	Free cholesterol in large LDL	1.2×10^−62^	12.6
rs4812975	20:44545460	*PLTP*	HDL	2.6×10^−3^	Mean HDL diameter	1.4×10^−15^	12.3
rs174554	11:61579463	*FADS1-2-3*	TG	1.7×10^−4^	Conc. of very large HDL particles	1.6×10^−16^	12.0
rs2156552	18:47181668	*LIPG*	TC	5.7×10^−3^	Phospholipids in medium HDL	2.9×10^−9^	6.3
rs10864726	1:230296153	*GALNT2*	HDL	4.9×10^−7^	Cholesterol esters in medium HDL	2.1×10^−12^	5.4
rs964184	11:116648917	*APOA1-C3-A4-A5*	TG	1.0×10^−30^	Triglycerides in small VLDL	1.4×10^−35^	4.8
rs4752801	11:47907641	*MTCH2-NUP160*	HDL	8.2×10^−6^	Free cholesterol in large HDL	1.4×10^−9^	3.8
rs55714927	17:7080316	*DLG4*	TG	1.2×10^−5^	Free cholesterol	3.9×10^−9^	3.5
rs12130333	1:63191777	*ANGPTL3*	TC	2.9×10^−4^	Conc. of very small VLDL particles	1.0×10^−7^	3.5
rs193042029	17:4667984	*TM4SF5*	TG	1.2×10^−5^	Free cholesterol	3.9×10^−9^	3.5
rs3890182	9:107647655	*ABCA1*	HDL	1.2×10^−4^	Mean LDL diameter	4.6×10^−8^	3.4
rs3177928	6:32412435	*HLA*	HDL	1.2×10^−4^	Triglycerides in IDL	4.6×10^−8^	3.4
rs2602836	4:100014805	*ADH5*	HDL	1.5×10^−2^	Total Cholesterol in very large HDL	7.3×10^−6^	3.3
rs58542926	19:19379549	*TM6SF2*	TG	1.4×10^−8^	Apolipoprotein B	9.1×10^−12^	3.2
rs141150988	19:38832410	*KCNK6*	TG	1.2×10^−2^	Triglycerides in CM and largest VLDL	1.6×10^−5^	2.9
rs115849089	8:19912370	*LPL*	TG	5.8×10^−13^	Triglycerides in small VLDL	3.6×10^−15^	2.2
rs116843064	19:8429323	*ANGPTL4*	TG	1.1×10^−7^	Phospholipids in large HDL	8.2×10^−10^	2.1
rs2972146	2:227100698	*IRS1*	HDL	2.5×10^−5^	Mean HDL diameter	2.0×10^−7^	2.1
rs182616603	4:75084732	*MTHFD2L*	TC	4.4×10^−7^	Conc. of small LDL particles	6.0×10^−9^	1.9
rs1129555	10:113910721	*GPAM*	TG	3.0×10^−4^	Mean HDL diameter	3.4×10^−6^	1.9
rs2287623	2:169830155	*ABCB11*	TC	2.4×10^−3^	Free cholesterol	3.4×10^−5^	1.8
rs13392272	2:21217490	*APOB*	LDL	3.0×10^−8^	Free cholesterol	5.7×10^−10^	1.7
rs1501908	5:156398169	*TIMD4*	TC	1.9×10^−3^	Triglycerides in IDL	4.7×10^−5^	1.6
rs737337	19:11347493	*ANGPTL8*	HDL	4.7×10^−4^	Phospholipids in large HDL	1.1×10^−5^	1.6
rs643531	9:15296034	*TTC39B*	HDL	8.3×10^−6^	Free cholesterol in very large HDL	3.1×10^−7^	1.4
rs72836561	17:41926126	*CD300LG*	HDL	1.1×10^−4^	Phospholipids in large HDL	9.8×10^−6^	1.1
rs112777051	16:57470884	*CIAPIN1-COQ9*	HDL	1.7×10^−4^	Cholesterol esters in medium HDL	4.3×10^−5^	0.6
rs649129	9:136154304	*ABO*	LDL	6.4×10^−7^	Conc. of very small VLDL particles	2.2×10^−7^	0.5
rs599839	1:109822166	*SORT1*	LDL	1.4×10^−14^	Ratio of apolipoprotein A-I to apolipoprotein B	6.6×10^−15^	0.3

Variants at established lipid trait loci for which the METSIM association (*P*<5×10^−5^) for a subclass trait was stronger than for any of four conventional lipid traits (HDL, LDL, TC, or TG). CM, chylomicrons. Chr, chromosome. IDL, intermediate-density lipoprotein.

§Log difference, −log10(*P*_subclass_/*P*_conventional_).

*Strongest associated conventional trait for the variant.

**Strongest associated subclass trait for the variant.

At less well-characterized and gene-dense loci, lipid and lipoprotein subclass associations may help suggest target genes or biological roles. At the gene-dense *MTCH2*-*NUP160* locus, rs4752801 was >3 log units more strongly associated with decreased free cholesterol in large HDL levels (*P*_subclass_ = 1.4×10^−9^) than any conventional trait (HDL-C, *P*_conventional_ = 8.2×10^−6^, Table 2). The pattern of association of rs4752801 with all 72 subclass traits (S7 Fig) is similar to the pattern of association and direction of effect for at least two other signals, rs737337 at *ANGPTL8* and rs1129555 at *GPAM*. *ANGPTL8* and *GPAM* are both regulated directly or indirectly by LXR, encoded by *NR1H3*,[49,50] which is a positional candidate gene at this locus[49,50]. Thus, the global pattern of association supports a contribution of *NR1H3* at the *MTCH2*-*NUP160* locus and suggests that the lipid and lipoprotein subclass traits can be a useful tool to help determine which genes underlie association signals.

We performed a similar analysis of lipoprotein associations at coronary artery disease (CAD) loci (S10 Table). Variants at the *APOA5/APOA1* locus were 5.2 log units more strongly associated with triglycerides in small VLDL than total triglycerides, and *APOE/APOC1* was 2.3 log units more strongly associated with ratio of apoA-I/apoB than any conventional trait. *APOA5* has been shown to affect VLDL concentrations and TG-rich particle metabolism, and the stronger association with the subclass trait is consistent with the known functions of these genes[51].

## Discussion

In this study we conducted GWAS for 72 lipid and lipoprotein subclass traits in 8,372 Finnish men participating the METSIM study, and focused on identifying association signals that had not been identified previously with any lipid or lipoprotein trait. From the literature of existing lipid and lipoprotein association studies, we identified 1,714 cholesterol, TG, lipid, and lipoprotein-associated variants. We trimmed this list based on LD (*r*^*2*^>0.95) to 885 variants to account for multiple known signals in a genome-wide conditional analysis. With this approach, we identified five novel signals at established lipid loci. We confirmed that signals were independent by reciprocal conditional analyses.

This analysis focused on NMR measurements of 72 lipid and lipoprotein subclasses, including four conventionally measured lipid traits: TC, TG, HDL-C, and LDL-C. 892 of the association signals were located at or near loci previously associated with one or more of the four conventional traits. Lipid and lipoprotein subclass traits have been linked to metabolic and cardiovascular diseases, which underline their clinical importance[8–10]. We identified variants at 30 loci that showed a more significant association with a subclass trait than one of the conventional lipid traits, consistent with previous observations[52].

The identification of multiple independent association signals at established GWAS loci can provide supporting evidence to identify target genes, as with monogenic disorders. Loci that harbor more than one association signal that affect transcriptional regulation of the same gene, or more than one coding variant that affect the same gene’s function, provide stronger evidence for a gene’s role in determining trait variability. Multiple signals can be critical to understanding the relationship between genetic variants and gene function, quantitative traits, and disease[53]. Multiple association signals at established loci can also be used to detect molecular interactions between coding and regulatory variants on protein levels[54]. In addition, multiple signals at the same locus may suggest that more than one nearby gene affects trait variation, and the association signals may represent different routes of transcriptional regulation. Further study of the multiple association signals at a locus may more precisely define the functional genetic mechanisms.

The gene-based tests of association at *LIPC* and *LIPG* identified new rare coding variants that may alter the function of these genes, and of lipid and lipoprotein subclass levels. While the missense variants identified here all have mean normalized lipoprotein trait values above the population mean, this type of analysis can help distinguish variants that lead to loss or decrease vs gain or increase of gene function[53]. As well, the rise of whole exome sequencing will likely uncover many more rare coding variants, including variants with unknown significance on gene function. While it is still unclear which variants included in the gene-based tests for *LIPC* and *LIPG* truly affect gene function, the comparison of trait values between carriers of different variants may be used to help interpret the potential role of these variants in individual carriers.

In conclusion, this GWAS of 72 lipid and lipoprotein subclass traits in 8,372 Finnish participants in the METSIM study identified associations with 42 loci previously identified only with the conventional lipid and lipoprotein traits[2], five novel signals associated with lipoprotein subclasses, and eight rare, potentially functional, coding variants at *LIPC* and *LIPG*. Our use of a dense reference panel of >15M variants combined with the high-throughput NMR-measured traits allowed us to conduct higher-resolution genetic analyses than reported previously. Functional analysis of the variants identified in this study is the next step to determine which variants and genes are affected, and replication of these lipid and lipoprotein subclass associations in women and in other ancestry groups will be useful to better understand the genetic architecture of lipid and lipoprotein metabolism.

## Materials and methods

### Ethics statement

The METSIM study was performed in accordance with the Helsinki Declaration and was approved by the Research Ethics Committee, Hospital District of Northern Savo (number 171/2004). All study participants gave their written informed consent.

### Study participants

Among the 10,197 participants in the METSIM study, we analyzed 8,372 non-diabetic individuals (mean age 57±7 SD years and BMI 26.8±3.8 kg/m^2^)[55].

### Subclass trait measurements

We measured the 72 lipid and lipoprotein traits from blood serum samples by proton NMR, as previously described[56]. Briefly, lipid samples are extracted and measured by proton NMR, and the NMR-spectra and automated phasing are compared to plate, background, and serum controls. Regression modeling is used to quantify the spectral areas to produce the quantified molecular data. The samples included 60 lipoprotein subclasses, 6 cholesterol and triglyceride measures, 3 cholesterol diameter measures, and 3 apolipoprotein measurements (S1 Table). Definition of the subclass traits has been previously described[56,57]. We visualized the Pearson correlation matrix between lipoprotein traits using a corrgram with the ellipse (https://cran.r-project.org/web/packages/ellipse/) and lattice packages (http://lattice.r-forge.r-project.org/) within R (S2 Fig)[58].

### Genotyping and imputation

We genotyped the study samples using the HumanOmniExpress-12v1_C BeadChip and Infinium HumanExome-12 v1.0 BeadChip, resulting in 631,879 and 236,849 variants, respectively. Imputation was performed using the GoT2D reference panel of >19M variants (SNPs, in-dels, and large deletions) based on whole-genome sequence of 2,657 Europeans consisting of German, Swedish, Finnish, and British participants; with the majority of the cohort comprised of Finns[59]. The resulting 15,144,991 variants were subjected to quality controls including sample- and variant-level controls for detecting sample contamination, sex and relatedness confirmation, and detection of sample outliers using principal-component analysis. To exclude samples with evidence of DNA contamination, we used BAFRegress v0.9 (http://genome.sph.umich.edu/wiki/BAFRegress). Based on principal component analysis, eighteen exome array sample duplicates, one individual each from seven monozygotic twin pairs, and twelve population outliers were removed from analysis. Due to sex chromosome inconsistencies, fourteen OmniExpress samples were removed. Samples with low genotype call rate (<95%) for either array were removed. Variants with low-mapping quality to build hg19, low genotype completeness (<95% for OmniExpress and <98% for exome array), or multi-allelic variants were removed. The remaining high quality variants were phased using Shape-It v2[60].

### Single-variant analysis

We tested for association using imputed dosages for all variants with summed minor allele count dosage >1 with each of the 72 lipid and lipoprotein traits assuming an additive model and accounting for cryptic relatedness using the EMMAX linear mixed model approach as implemented in EPACTS (http://genome.sph.umich.edu/wiki/epacts). Traits were adjusted for age, age^2^, smoking status, and lipid lowering medication. Residuals were inverse normalized. To assess the level of genomic inflation, we calculated the genomic control statistic (λ_GC_) for all of the trait-variant associations using R[58] (S1 Fig). Reported effect size regression coefficients (betas) sizes are given in standard deviation units. The rare lead associated variants that were imputed and had MAF<0.01 were tested for genotype accuracy by using TaqMan assays (Thermo Fisher Scientific) or Sanger sequencing in 499 METSIM participants who carried one or more rare alleles at these variants. Variants that had >10% discordance between the imputed genotype and the sequenced genotype in the examined individuals were removed from the analysis. Variants with MAF<0.001 were excluded from analysis (S5 Table).

### Compilation of existing lipid and lipoprotein trait associated variants

To identify variant association signals distinct or independent from those reported previously, we identified variants previously reported to be associated with any cholesterol, lipid, lipoprotein, or triglyceride trait. We performed a literature review of GWAS and sequencing studies using PubMed (https://www.ncbi.nlm.nih.gov/pubmed/) and Google Scholar (https://scholar.google.com/), screened a GWAS Catalog (http://www.ebi.ac.uk/gwas/), and used SNIPPER (https://csg.sph.umich.edu/boehnke/snipper/) to query publicly accessible databases. The resulting curated list contained 1,714 variants, at >150 loci from 33 studies (S3 Table). The resulting curated list contained 1,714 variants, at >150 loci from 33 studies (S3 Table). We used this list to represent the known genome-wide lipid and lipoprotein-associated variants.

### Conditional analyses

We LD-pruned (r^2^>0.95) the compiled list of 1,714 variants (S3 Table) to 885 variants (S4 Table) and we used this list (n = 885) in genome-wide conditional tests of association; this stringent LD threshold facilitates conditioning on multiple known signals at a locus. Signals that remained significant (*P*_*conditional*_<5×10^−8^) after genome-wide conditional analysis were considered novel and further tested by single-variant conditional analyses to determine independence. At each of the five loci (Table 1), variants within 1 Mb up- and downstream of the lead variant and on the compiled list of 1,714 variants were included in single-marker conditional analyses (S6 Table). Signals that remained significant (*P*_*single*_<5×10^−8^) after single-variant conditional analysis were considered independent. Signals that only achieved a significance threshold of *P*_*single*_<5×10^−6^ after single-variant conditional analysis were considered distinct. The single-variant conditional analyses considered variants within 1 Mb of the signal, which accounts for <1% of the genome. Therefore, the significance thresholds for the distinct and independent additional signals are conservative. At each locus, we validated that signals were distinct/independent by reciprocal conditional analysis with the putative novel lead associated variant for the trait. Additionally, the association data for the novel signals was adjusted for each of the four conventional traits (HDL-C, LDL-C, TC, and TG), and the effect of the association is reported in S7 Table.

### Comparison of subclass trait associations with conventional lipid and lipoprotein trait associations

For each of the 885 lipid/lipoprotein-associated variants (S4 Table), we determined whether the variant showed stronger association with one of the 68 subclass traits compared to the four conventional lipid traits (TC, TG, LDL-C, HDL-C). Variants were included for comparison if the variant association with a subclass trait satisfied *P*<5×10^−5^ and if–log_10_pvalue for subclass trait association was greater than any of the four conventional lipid traits.

### Gene-based tests of association

To determine the contribution of rare coding variants, we used the Optimal Sequence Kernel Association Test (SKAT-O) with EMMAX, as implemented in EPACTS, to test for gene-based associations with the 72 lipoprotein traits[61]. Only coding variants directly genotyped on the OmniExpress or Exome array were included, resulting in 709,600 variants. Since SKAT-O requires no missing data, we imputed missing genotype data with the variant mean genotype. We annotated coding variants using VEP. These annotations were the basis for four masks that we implemented in the gene-based tests, as previously described[53]. Briefly, the four masks were: Protein-Truncating Variants (PTV): no MAF limit; variants are nonsense, frameshift, or essential splice variants. PTV+missense: MAF<1%; all PTVs and missense variants. PTV+Nonsynonymous strict (NS_strict_): no MAF limit; all PTVs and missense variants predicted as deleterious by five variant annotation algorithms: LRT, Mutation Taster, PolyPhen2-HumDiv, PolyPhen2-HumVar, and SIFT. PTV+NS_strict_+NS_broad_: MAF<1%; all variants in PTV+NS_strict_ and variants predicted to be deleterious by any of the five algorithms above. Only genes containing two or more variants in a given mask were tested. We conducted gene-based conditional analyses to determine whether a single variant or a net-effect of multiple variants could explain the observed association signal.

### Variant annotation

To better characterize the novel signals from this study, we determined whether the associated lead variants and LD proxies (*r*^*2*^>0.7 in METSIM) at each signal were within ChIP-seq peaks of epigenomic transcriptional regulatory elements (S8 Table). We built lists of such elements using data from the ENCODE Consortium[62] and Roadmap Epigenomics Project[63]. We used datasets from three lipid and cholesterol relevant tissues (adipose, blood, and liver datasets) that were comprised of experimentally defined regions of transcription factor binding sites (ChIP-seq), open chromatin (DNase- and FAIRE-seq), and histone modification marks (H3K4me1, H3K4me2, and H3K4me3, H3K27ac, and H3K9ac).

## Supporting information

S1 Fig Distribution of the METSIM lambda genomic control (GC) for the 72 lipoproteins. (PDF)

S2 Fig Correlogram showing Pearson correlations between lipoprotein traits. Correlogram showing Pearson correlations between lipoprotein traits. Rows and columns are ordered according to a complete linkage hierarchical clustering of traits based on the Pearson correlation matrix. Percent correlation is shown for each trait-trait comparison. The color scale is from red (negative correlation) to blue (positive correlation). The shapes are representations of the correlation with a circle representing ‘no correlation’, ellipse representing ‘moderate correlation’, and a diagonal line representing ‘high correlation’.
(PDF)

S3 Fig Manhattan plots of traits. Manhattan plots for the five traits with signals identified from this study. X-axis shows the chromosomes, and the y-axis is the–log_10_(*P*value) for the variant-trait association. The horizontal line is at the cutoff *P* = 5×10^−8^.
(PDF)

S4 Fig Plots of the five association signals after conditioning on the 885 known lipid signals. Each circle represents a single variant. The color is based on LD (r^2^) between each variant and the reference variant (purple diamond); X-axis, genomic (GRCh37/hg19) position in Mb; Left y-axis, p- value of variant-trait association in–log_10_; Right y-axis, local estimates of genomic recombination rate in cM/Mb, represented by blue vertical lines.
(PDF)

S5 Fig Unconditional plots of the five lipid and lipoprotein subclass-associated loci. Each circle represents a single variant. The color is based on LD (r^2^) between each variant and the reference variant (purple diamond); X-axis, genomic (GRCh37/hg19) position in Mb; Left y-axis, p-value of variant-trait association in–log_10_; Right y-axis, local estimates of genomic recombination rate in cM/Mb, represented by blue vertical lines.
(PDF)

S6 Fig Association of all 72 lipoprotein/lipid traits with the variants in Table 1. The *P*value is shown in -log_10_ and in the direction (+ or −) of the effect (Beta). The red line denotes the significance cutoff of P≤5E-8. The red asterisk indicates the most significantly associated trait. CAD, coronary artery disease.
(PDF)

S7 Fig Association of all 72 lipoprotein/lipid traits with the variants in Table 2. The *P*value is shown in -log_10_ and in the direction (+ or −) of the effect (Beta). The red line denotes the significance cutoff of P≤5E-8. The red asterisk indicates the most significantly associated trait. CAD, coronary artery disease.
(PDF)

S1 Table Characteristics of 8,372 METSIM study participants and 72 lipoprotein subclasses and triglyceride measures. (XLSX)

S2 Table 3,784 variants associated (*P*<5×10^−8^) with at least one of the 72 lipoprotein traits. 3,784 unique variants that comprise the significant (P<5×10^−8^) 32,524 trait-variant associations; Chr:position, hg19 chromosome and position; Genotype counts for homozygous reference allele/heterozygous/homozygous alternate allele; MAC, minor allele count; MAF, minor allele frequency; P-value, best unconditional p-value for any of the 72 traits; Beta, effect size of the alternate allele; Trait, trait with strongest association.
(XLSX)

S3 Table Previously reported associations between 1,714 variants and one or more lipid or lipoprotein traits. Variants and association data in this table were collected from 33 published GWAS, fine-mapping, and exome-sequencing studies. The reported trait, Beta, and P-value are taken from the study with the lowest p-value. N.R., data not reported in the study; Chr, chromosome; Position, hg19; Trait, strongest associated trait from study shown; EA, effect allele; NEA, non-effect allele; N, sample size of the study; Reference, the first study in the list is the study with the lowest p-value for the given trait.
(XLSX)

S4 Table 885 variants used for the conditional analysis (see Methods). Variants on this table were trimmed from the 1,714 variants on S3 Table by using an r^2^>0.95 cutoff. The reported trait, Beta, and P-value are taken from the study with the lowest p-value. N.R., data not reported in the study; Chr, chromosome; Position, hg19; Trait, strongest associated trait from study shown; EA, effect allele; NEA, non-effect allele; N, sample size of the study; Reference, the first study in the list is the study with the lowest p-value for the given trait.
(XLSX)

S5 Table Variants that remained significantly associated (*P* <5×10^−8^) with at least one of the 72 lipid or lipoprotein traits after conditioning on 885 known variants. Trait, subclass trait (defined in S1 Table); Chr, chromosome; Genotype counts for homozygous reference allele/heterozygous/homozygous alternate allele; MAC, minor allele count; MAF, minor allele frequency; P-value, p-value for the variant after conditioning on 885 known lipid-associated variants (S4 Table); Beta, effect size of the alternate allele; R2, variance explained.
(XLSX)

S6 Table Results of single-marker and reciprocal conditional analyses for the associated variants from this study. Lead variant, the most associated variant for the Lead Trait at a given locus; Lead trait, the lipoprotein subclass or triglyceride measure with lowest p-value across the 72 traits; Locus, biologically relevant gene within 1 Mb of lead variant; Chr, chromosome; MAF, minor allele frequency; *P*_*discovery*_, unconditional p-value for the lead variant and trait; *P*_*conditional*_, p-value for the lead variant after conditioning on 885 known lipid GWAS variants (S4 Table); Conditional variant, known lipid or lipoprotein associated variants used for single-marker conditional analyses; *P*_*single*_, p-value for the Lead variant after conditioning on the Conditional variant; *P*_*trait*_, unconditional p-value of the Conditional variant for the Lead trait; *P*_*reciprocal*_, p-value of the Conditional variant after conditioning on the Lead variant; Conc, concentration; At chromosome 18, the variants identified in the gene-based test for *LIPG* are in italics. § The effect of the unconditional p-value for the lead variant and trait after adjusting for each of the four conventional traits.
(XLSX)

S7 Table Results of conventional trait conditional analyses for the associated variants from this study. Lead variant, the most associated variant for the Lead Trait at a given locus; Lead trait, the lipoprotein subclass or triglyceride measure with lowest p-value across the 72 traits; Locus, biologically relevant gene within 1 Mb of lead variant; Chr, chromosome; MAF, minor allele frequency; *P*_discovery_, unconditional p-value for the lead variant and trait. § *P*_HDL_, *P*_LDL_, *P*_TC_, and *P*_TG_, p-value for the lead variant and trait after adjusting for each of the four traits.
(XLSX)

S8 Table Lipoprotein subclass and triglyceride measure loci associated variants that overlap epigenetic evidence of regulatory elements. Variants listed were in LD (r^2^>0.7) with the lead trait variant (in bold) from this study and overlapped a regulatory element in relevant tissue datasets. Abbreviations of the tissues tested for overlapping of regulatory elements are: A, adipose; B, blood; L, liver. Chr, chromosome; Nearest coding TSS, distance from nearest GENCODEv12 basic annotation transcription start site. Negative distance indicates the variant is upstream of the TSS relative to the direction of transcription; N, total number of overlapping datasets across experiments and cell types; Open chromatin, variants overlapping FAIRE/DNase hypersensitivity elements; ChIP-seq Peak, Variant overlaps ChIP-seq peaks. Transcription factor name:Tissue.
(XLSX)

S9 Table Results of single-marker conditional analyses for the gene-based association data from this study. Gene, gene tested in gene-based association tests; Lead trait, the lipoprotein subclass or triglyceride measure with lowest p-value across the 72 traits; *P*_*gene*_, p-value for the gene and trait; Conditional variant, known lipid or lipoprotein associated variants used for single-marker conditional analyses; Chr:Position, the hg19 chromosome and base-pair position of the Conditional variant; P, p-value for the gene-based association after conditioning on the Conditional variant.
(XLSX)

S10 Table Comparison of METSIM association data between conventional lipid traits and subclass traits at established coronary artery disease loci. Variants at established CAD loci for which the METSIM association (*P* <5×10^−5^) for a subclass trait was stronger than for any of four conventional lipid traits (HDL, LDL, TC, or TG). Log difference, log_10_(*P*_subclass_/*P*_conventional_). Chr, chromosome.
(XLSX)
