# Supplementary figures and images for: Common, low-frequency, and rare genetic variants associated with lipoprotein subclasses and triglyceride measures in Finnish men from the METSIM study
Source: PLoS Genet. 2017 Oct 30;13(10):e1007079. doi: 10.1371/journal.pgen.1007079 (PMC5679656; doi:10.1371/journal.pgen.1007079)

**S1 Fig. Distribution of the METSIM lambda genomic control (GC) for the 72 lipoprotein traits**

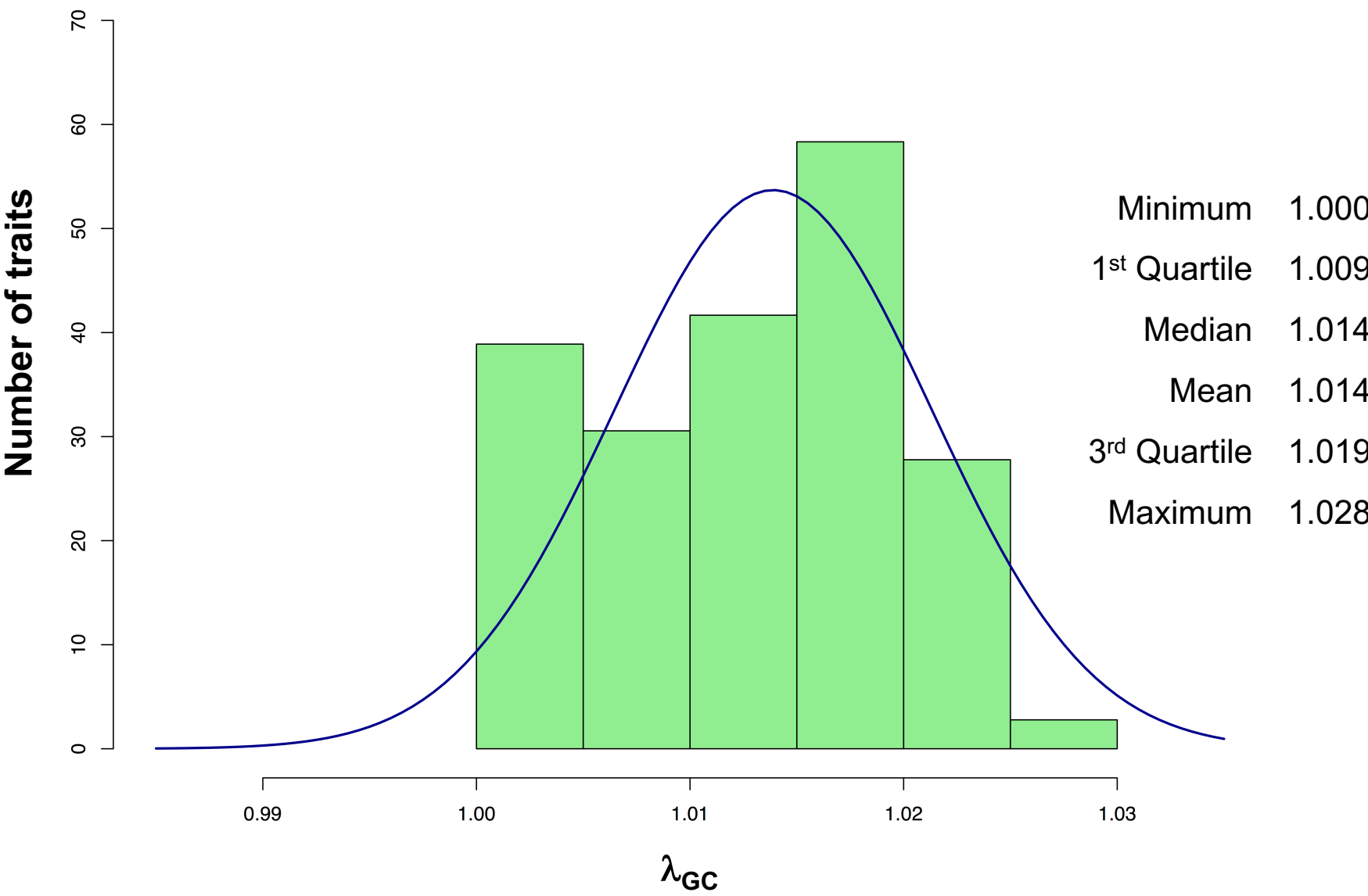

Supplement: S1 Fig — (PDF) [file pgen.1007079.s001.pdf]
