# Supplementary material for: Common, low-frequency, and rare genetic variants associated with lipoprotein subclasses and triglyceride measures in Finnish men from the METSIM study
Source: PLoS Genet. 2017 Oct 30;13(10):e1007079. doi: 10.1371/journal.pgen.1007079 (PMC5679656; doi:10.1371/journal.pgen.1007079)

S2 Fig. Correlogram showing Pearson correlations between lipoprotein traits. Correlogram showing Pearson correlations between lipoprotein traits. Rows and columns are ordered according to a complete linkage hierarchical clustering of traits based on the Pearson correlation matrix. Percent correlation is shown for each trait-trait comparison. The color scale is from red (negative correlation) to blue (positive correlation). The shapes are representations of the correlation with a circle representing ‘no correlation’, ellipse representing ‘moderate correlation’, and a diagonal line representing ‘high correlation’.

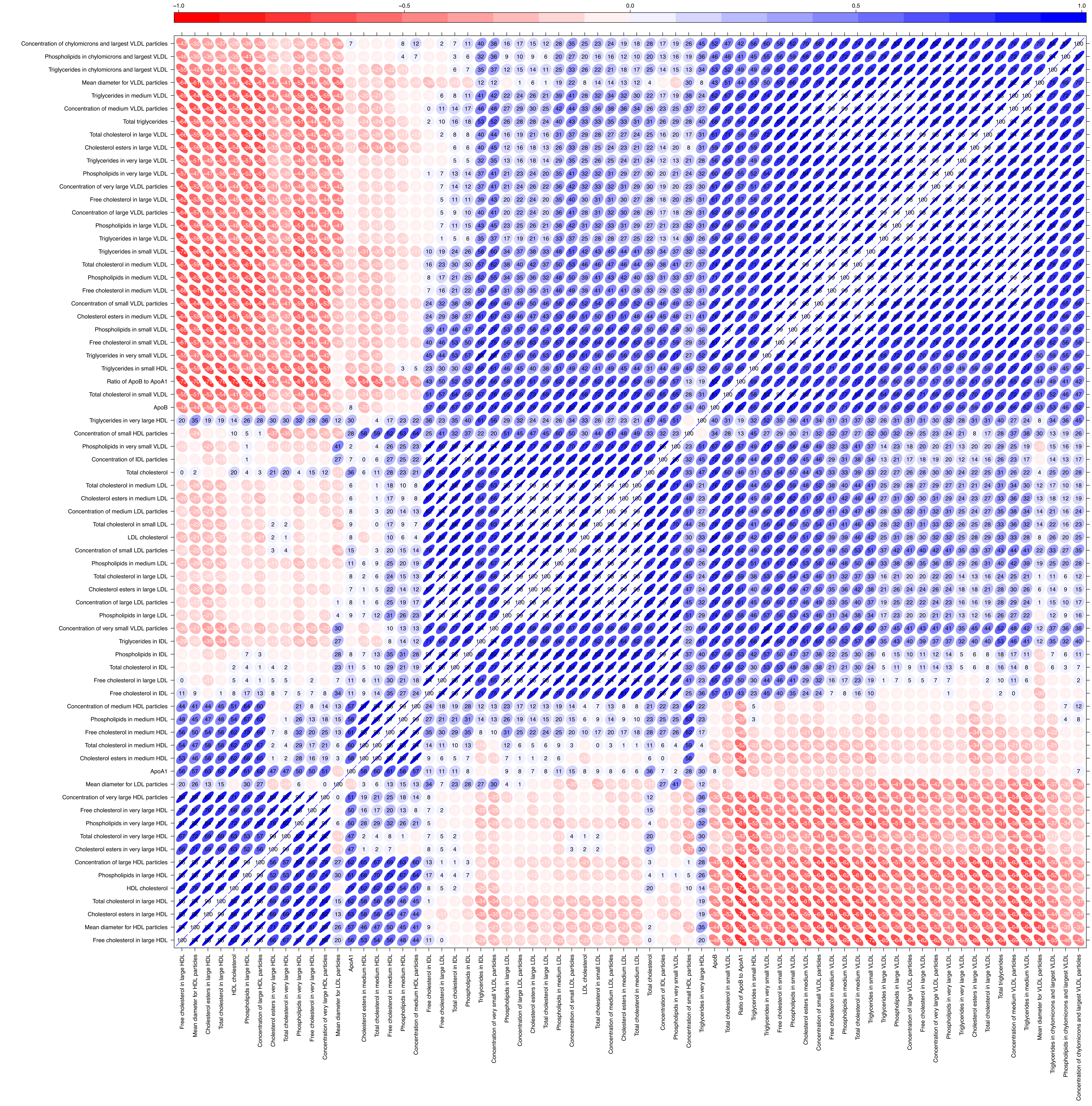

Supplement: S2 Fig — Correlogram showing Pearson correlations between lipoprotein traits. Rows and columns are ordered according to a complete linkage hierarchical clustering of traits based on the Pearson correlation matrix. Percent correlation is shown for each trait-trait comparison. The color scale is from red (negative correlation) to blue (positive correlation). The shapes are representations of the correlation with a circle representing ‘no correlation’, ellipse representing ‘moderate correlation’, and a diagonal line representing ‘high correlation’. (PDF) [file pgen.1007079.s002.pdf]
