# Supplementary material for: Common, low-frequency, and rare genetic variants associated with lipoprotein subclasses and triglyceride measures in Finnish men from the METSIM study
Source: PLoS Genet. 2017 Oct 30;13(10):e1007079. doi: 10.1371/journal.pgen.1007079 (PMC5679656; doi:10.1371/journal.pgen.1007079)
Supplement: S3 Fig — Manhattan plots for the five traits with signals identified from this study. X-axis shows the chromosomes, and the y-axis is the–log10(Pvalue) for the variant-trait association. The horizontal line is at the cutoff P = 5×10^−8^. (PDF) [file pgen.1007079.s003.pdf]

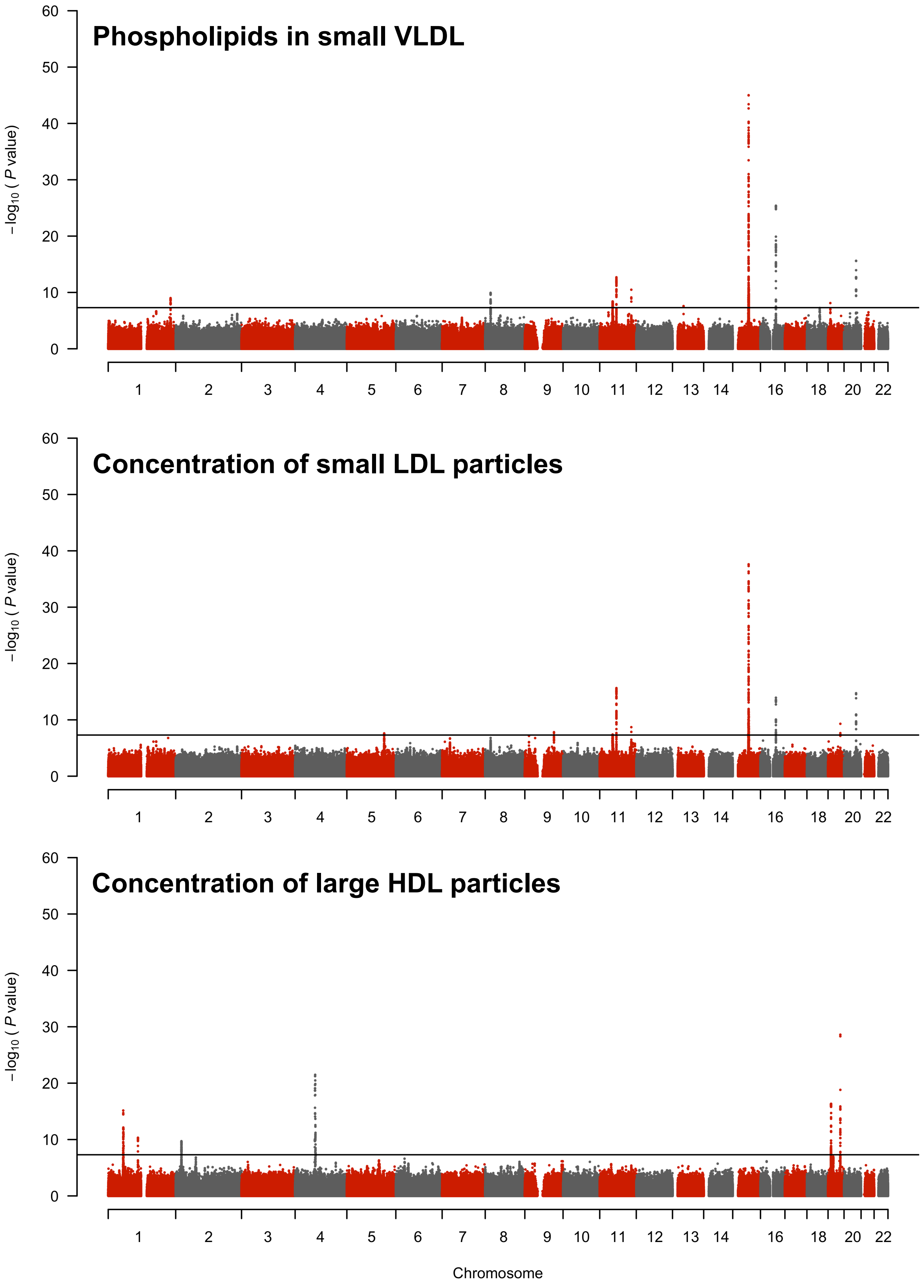
Phospholipids in small VLDL
Concentration of small LDL particles
Concentration of large HDL particles
−log10 (P value)
0
10
20
30
40
50
60
1
2
3
4
5
6
7
8
9
10
11
12
13
14
16
18
20
22
Chromosome

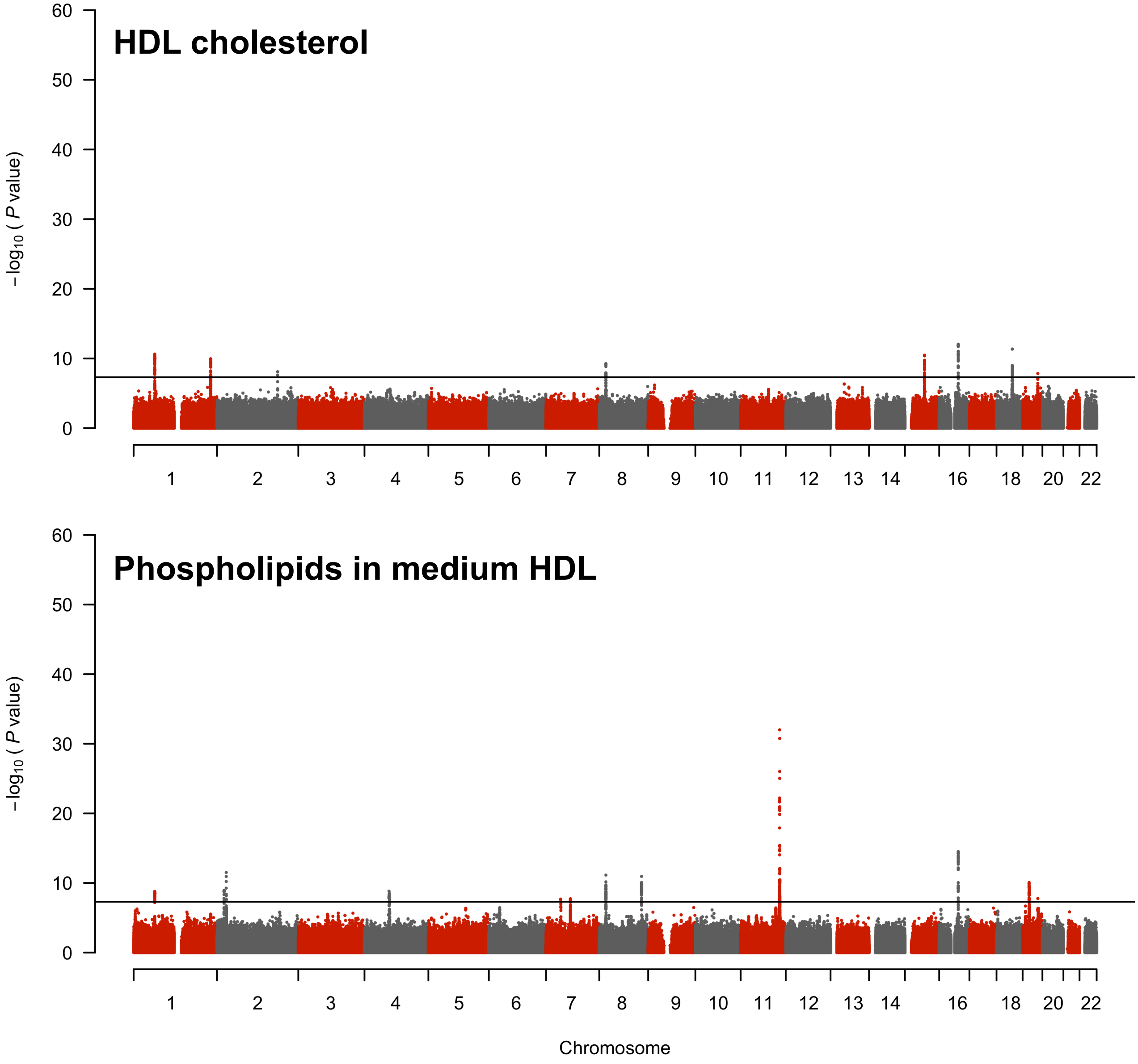

S3 Fig. Manhattan plots of traits. Manhattan plots for the five traits with signals identified from this study. X-axis shows the chromosomes, and the y-axis is the $-\log_{10}(P\text{value})$ for the variant-trait association. The horizontal line is at the cutoff $P=5\times10^{-8}$.
