# Supplementary material for: Common, low-frequency, and rare genetic variants associated with lipoprotein subclasses and triglyceride measures in Finnish men from the METSIM study
Source: PLoS Genet. 2017 Oct 30;13(10):e1007079. doi: 10.1371/journal.pgen.1007079 (PMC5679656; doi:10.1371/journal.pgen.1007079)
Supplement: S4 Fig — Each circle represents a single variant. The color is based on LD (r2) between each variant and the reference variant (purple diamond); X-axis, genomic (GRCh37/hg19) position in Mb; Left y-axis, p- value of variant-trait association in–log10; Right y-axis, local estimates of genomic recombination rate in cM/Mb, represented by blue vertical lines. (PDF) [file pgen.1007079.s004.pdf]

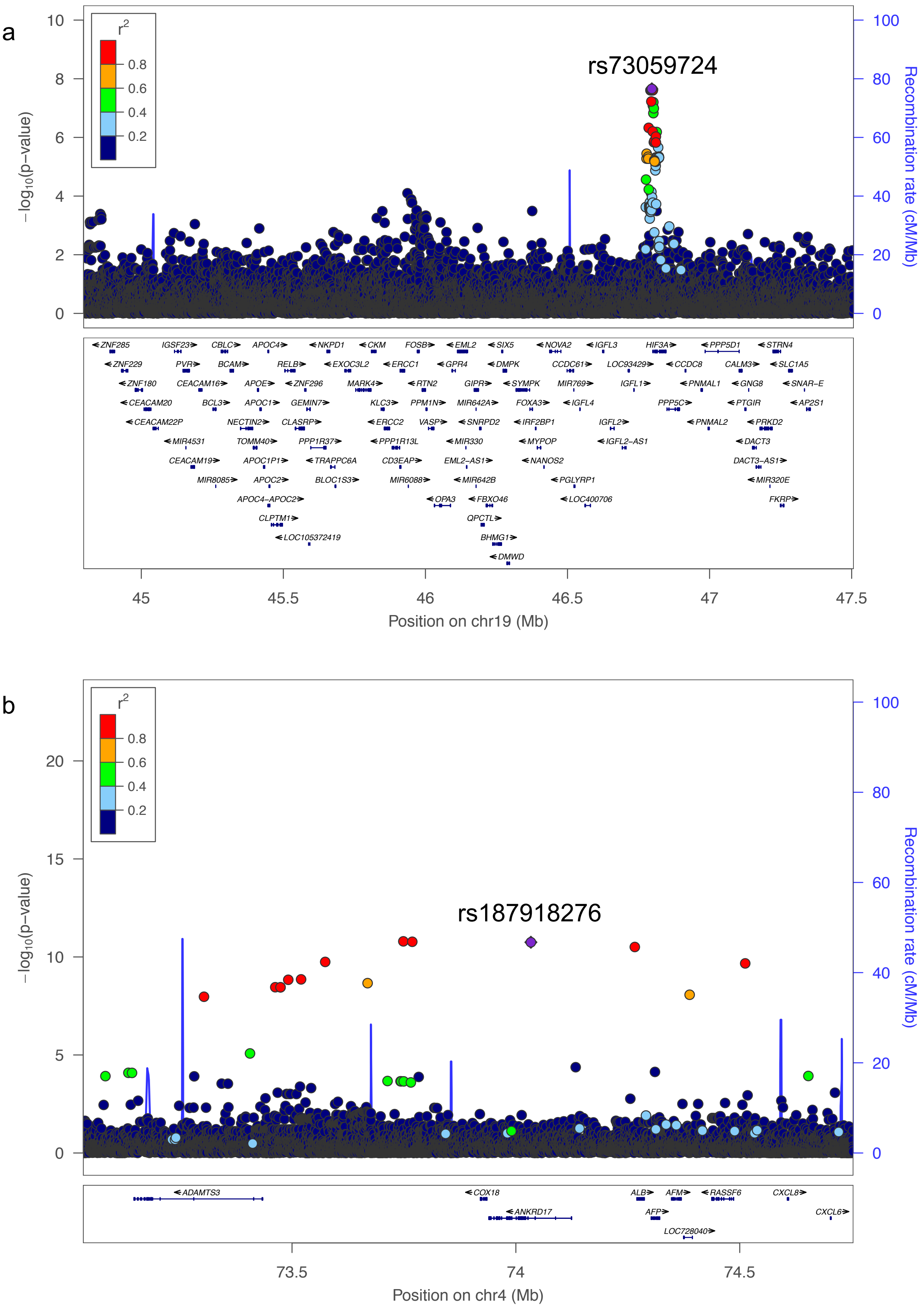

a
rs73059724
−log10(p−value)
Recombination rate (cM/Mb)
r2
0.8
0.6
0.4
0.2
10
8
6
4
2
0
100
80
60
40
20
0
ZNF285
IGSF23
CBLC
APOC4
NKPD1
CKM
FOSB
EML2
SIX5
NOVA2
IGFL3
HIF3A
PPP5D1
STRN4
ZNF229
PVR
BCAM
RELB
EXOC3L2
ERCC1
GPR4
DMPK
CCDC61
LOC93429
CCDC8
CALM3
SLC1A5
ZNF180
CEACAM16
APOE
ZNF296
MARK4
RTN2
GIPR
SYMPK
MIR769
IGFL1
PNMAL1
GNG8
SNAR−E
CEACAM20
BCL3
APOC1
GEMIN7
KLC3
PPM1N
MIR642A
FOXA3
IGFL4
PPP5C
PTGIR
AP2S1
CEACAM22P
NECTIN2
CLASRP
ERCC2
VASP
SNRPD2
IRF2BP1
IGFL2
PNMAL2
PRKD2
MIR4531
TOMM40
PPP1R37
PPP1R13L
MIR330
MYPOP
IGFL2−AS1
DACT3
CEACAM19
APOC1P1
TRAPPC6A
CD3EAP
EML2−AS1
NANOS2
DACT3−AS1
MIR8085
APOC2
BLOC1S3
MIR6088
MIR642B
PGLYRP1
MIR320E
APOC4−APOC2
OPA3
FBXO46
LOC400706
FKRP
CLPTM1
QPCTL
LOC105372419
BHMG1
DMWD
45
45.5
46
46.5
47
47.5
Position on chr19 (Mb)
b
rs187918276
−log10(p−value)
Recombination rate (cM/Mb)
r2
0.8
0.6
0.4
0.2
20
15
10
5
0
100
80
60
40
20
0
ADAMTS3
COX18
ALB
AFM
RASSF6
CXCL8
ANKRD17
AFP
CXCL6
LOC728040
73.5
74
74.5
Position on chr4 (Mb)

c

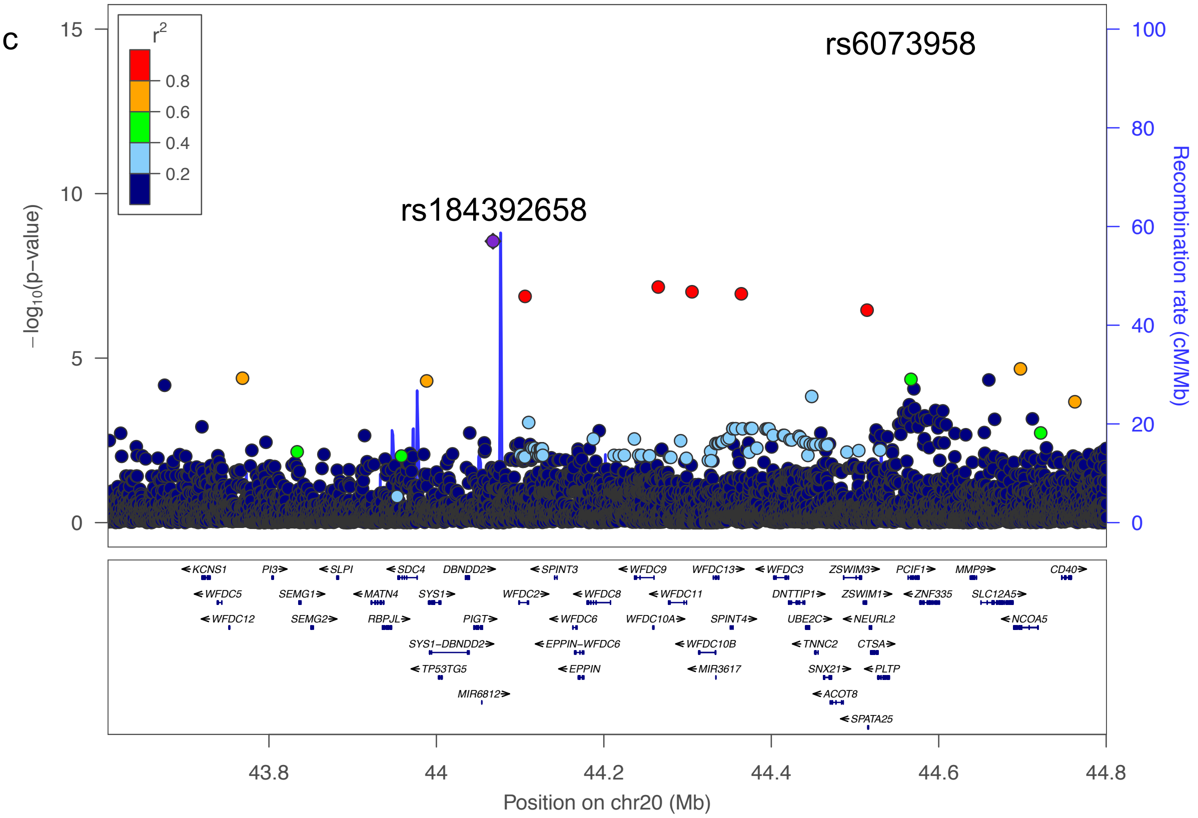

rs6073958
rs184392658
r²
0.8
0.6
0.4
0.2
15
10
5
0
100
80
60
40
20
−log10(p−value)
Recombination rate (cM/Mb)
KCNS1
PI3
SLPI
SDC4
DBNDD2
SPINT3
WFDC9
WFDC13
WFDC3
ZSWIM3
PCIF1
MMP9
CD40
WFDC5
SEMG1
MATN4
SYS1
WFDC2
WFDC8
WFDC11
DNTTIP1
ZSWIM1
ZNF335
SLC12A5
WFDC12
SEMG2
RBPJL
PIGT
WFDC6
WFDC10A
SPINT4
UBE2C
NEURL2
NCOA5
SYS1−DBNDD2
EPPIN−WFDC6
WFDC10B
TNNC2
CTSA
TP53TG5
EPPIN
MIR3617
SNX21
PLTP
MIR6812
ACOT8
SPATA25
43.8
44
44.2
44.4
44.6
44.8
Position on chr20 (Mb)

d

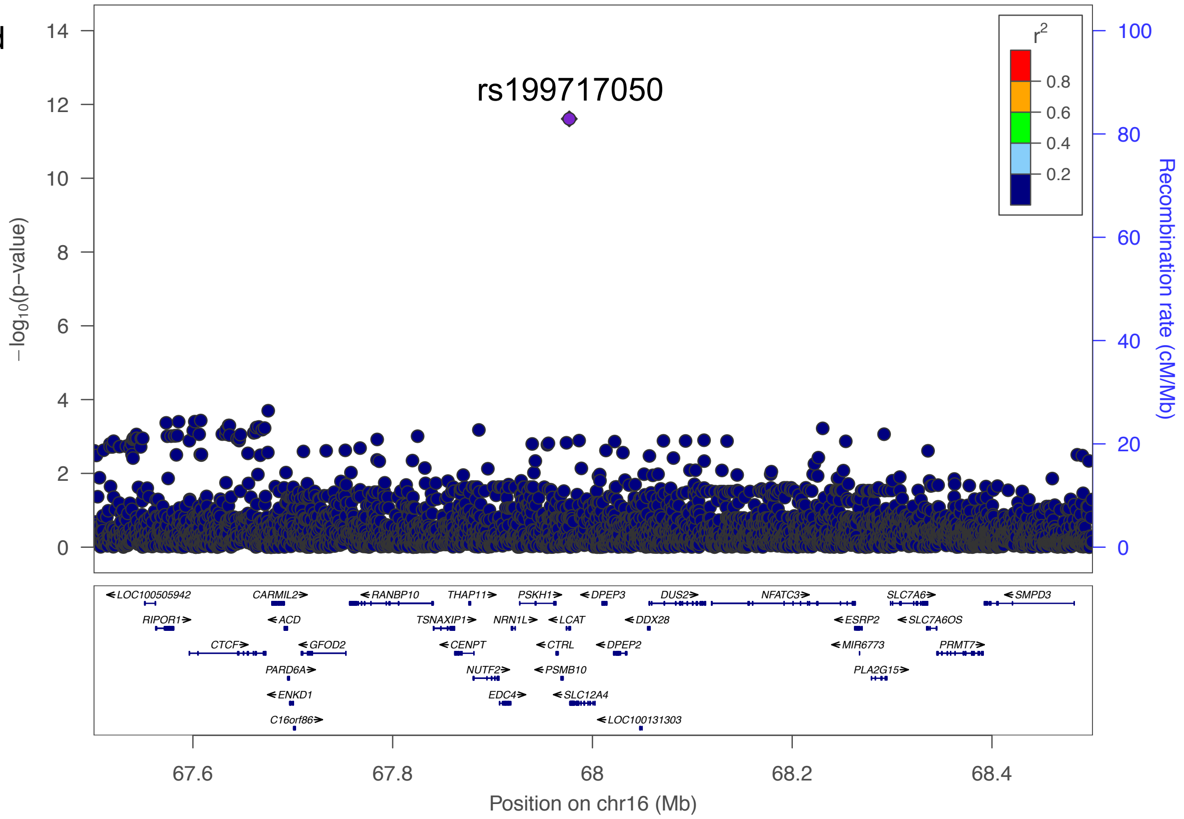

rs199717050
r²
0.8
0.6
0.4
0.2
14
12
10
8
6
4
2
0
100
80
60
40
20
−log10(p−value)
Recombination rate (cM/Mb)
LOC100505942
CARMIL2
RANBP10
THAP11
PSKH1
DPEP3
DUS2
NFATC3
SLC7A6
SMPD3
RIPOR1
ACD
TSNAXIP1
NRN1L
LCAT
DDX28
ESRP2
SLC7A6OS
CTCF
GFOD2
CENPT
CTRL
DPEP2
MIR6773
PRMT7
PARD6A
NUTF2
PSMB10
PLA2G15
ENKD1
EDC4
SLC12A4
C16orf86
LOC100131303
67.6
67.8
68
68.2
68.4
Position on chr16 (Mb)

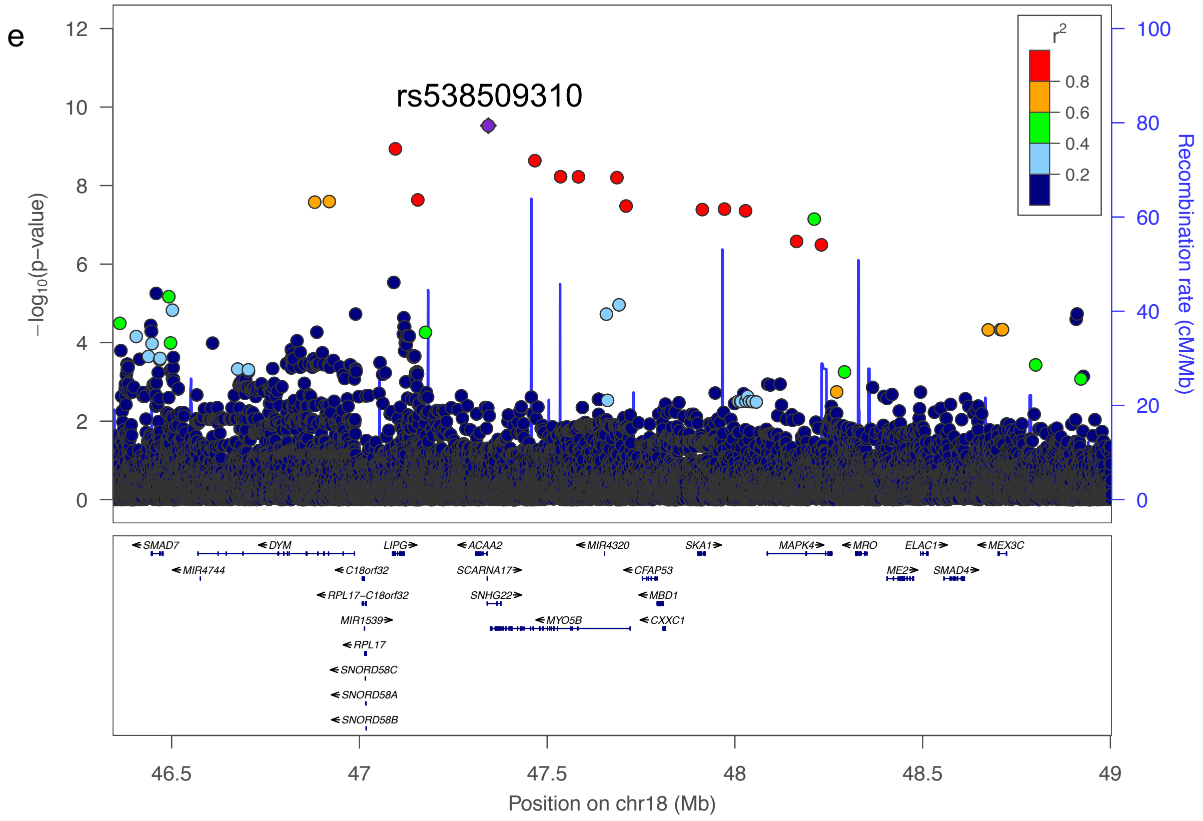

S4 Fig. Plots of the five association signals after conditioning on the 885 known lipid signals. Each circle represents a single variant. The color is based on LD (r^2) between each variant and the reference variant (purple diamond); X-axis, genomic (GRCh37/hg19) position in Mb; Left y-axis, p-value of variant-trait association in $-\log_{10}$; Right y-axis, local estimates of genomic recombination rate in cM/Mb, represented by blue vertical lines.
