# Supplementary material for: Common, low-frequency, and rare genetic variants associated with lipoprotein subclasses and triglyceride measures in Finnish men from the METSIM study
Source: PLoS Genet. 2017 Oct 30;13(10):e1007079. doi: 10.1371/journal.pgen.1007079 (PMC5679656; doi:10.1371/journal.pgen.1007079)
Supplement: S5 Fig — Each circle represents a single variant. The color is based on LD (r2) between each variant and the reference variant (purple diamond); X-axis, genomic (GRCh37/hg19) position in Mb; Left y-axis, p-value of variant-trait association in–log10; Right y-axis, local estimates of genomic recombination rate in cM/Mb, represented by blue vertical lines. (PDF) [file pgen.1007079.s005.pdf]

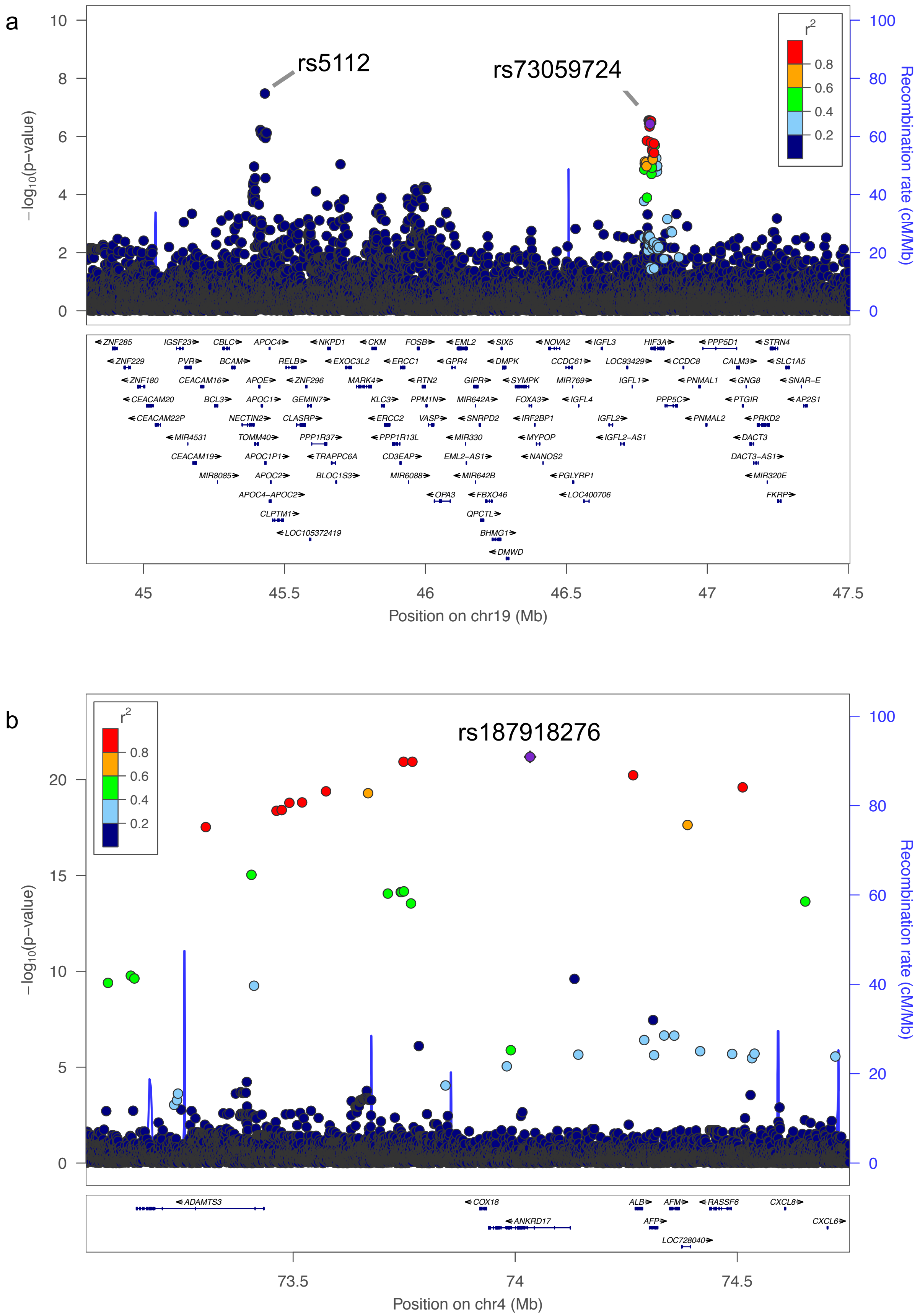
a
rs5112
rs73059724
r2
0.8
0.6
0.4
0.2
−log10(p−value)
Recombination rate (cM/Mb)
ZNF285 IGSF23 CBLC APOC4 NKPD1 CKM FOSB EML2 SIX5 NOVA2 IGFL3 HIF3A PPP5D1 STRN4
ZNF229 PVR BCAM EXOC3L2 ERCC1 GPR4 DMPK CCDC61 LOC93429 CCDC8 CALM3 SLC1A5
ZNF180 CEACAM16 APOE ZNF296 MARK4 RTN2 GIPR SYMPK MIR769 IGFL1 PNMAL1 GNG8 SNAR−E
CEACAM20 BCL3 APOC1 GEMIN7 KLC3 PPM1N MIR642A FOXA3 IGFL4 PPP5C PTGIR AP2S1
CEACAM22P NECTIN2 CLASRP ERCC2 VASP SNRPD2 IRF2BP1 IGFL2 PNMAL2 PRKD2
MIR4531 TOMM40 PPP1R37 PPP1R13L MIR330 MYPOP IGFL2−AS1 DACT3
CEACAM19 APOC1P1 TRAPPC6A CD3EAP EML2−AS1 NANOS2 DACT3−AS1
MIR8085 APOC2 BLOC1S3 MIR6088 MIR642B PGLYRP1 MIR320E
APOC4−APOC2 OPA3 FBXO46 LOC400706 FKRP
CLPTM1 QPCTL
LOC105372419 BHMG1
DMWD
Position on chr19 (Mb)
b
rs187918276
r2
0.8
0.6
0.4
0.2
−log10(p−value)
Recombination rate (cM/Mb)
ADAMTS3 COX18 ALB AFM RASSF6 CXCL8
ANKRD17 AFP CXCL6
LOC728040
Position on chr4 (Mb)

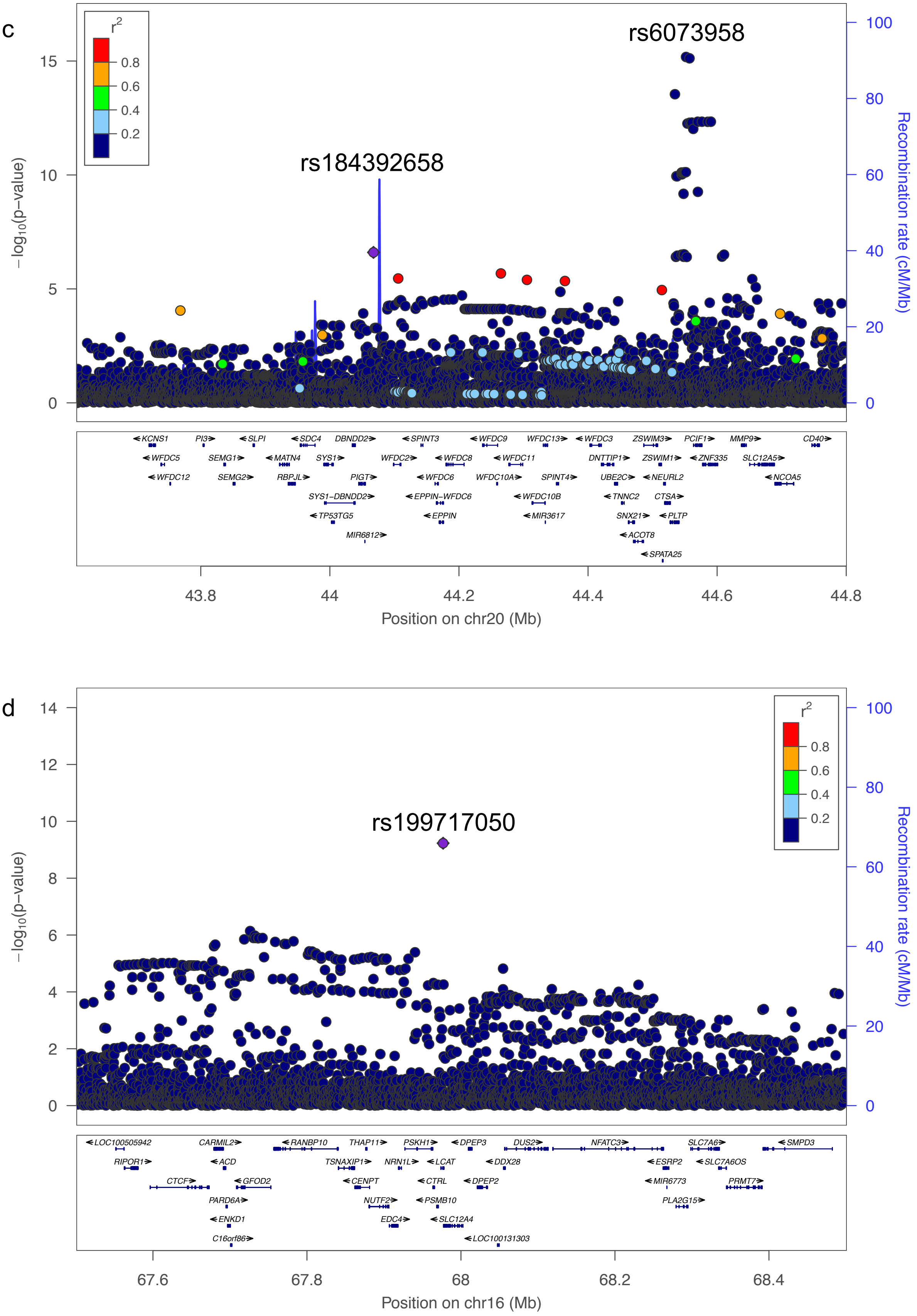
c
rs6073958
rs184392658
r2
0.8
0.6
0.4
0.2
−log10(p−value)
Recombination rate (cM/Mb)
KCNS1
PI3
SLPI
SDC4
DBNDD2
SPINT3
WFDC9
WFDC13
WFDC3
ZSWIM3
PCIF1
MMP9
CD40
WFDC5
SEMG1
MATN4
SYS1
WFDC2
WFDC8
WFDC11
DNTTIP1
ZSWIM1
ZNF335
SLC12A5
WFDC12
SEMG2
RBPJL
PIGT
WFDC6
WFDC10A
SPINT4
UBE2C
NEURL2
NCOA5
SYS1−DBNDD2
EPPIN−WFDC6
WFDC10B
TNNC2
CTSA
TP53TG5
EPPIN
MIR3617
SNX21
PLTP
MIR6812
ACOT8
SPATA25
43.8
44
44.2
44.4
44.6
44.8
Position on chr20 (Mb)
d
rs199717050
r2
0.8
0.6
0.4
0.2
−log10(p−value)
Recombination rate (cM/Mb)
LOC100505942
CARMIL2
RANBP10
THAP11
PSKH1
DPEP3
DUS2
NFATC3
SLC7A6
SMPD3
RIPOR1
ACD
TSNAXIP1
NRN1L
LCAT
DDX28
ESRP2
SLC7A6OS
CTCF
GFOD2
CENPT
CTRL
DPEP2
MIR6773
PRMT7
PARD6A
NUTF2
PSMB10
PLA2G15
ENKD1
EDC4
SLC12A4
C16orf86
LOC100131303
67.6
67.8
68
68.2
68.4
Position on chr16 (Mb)

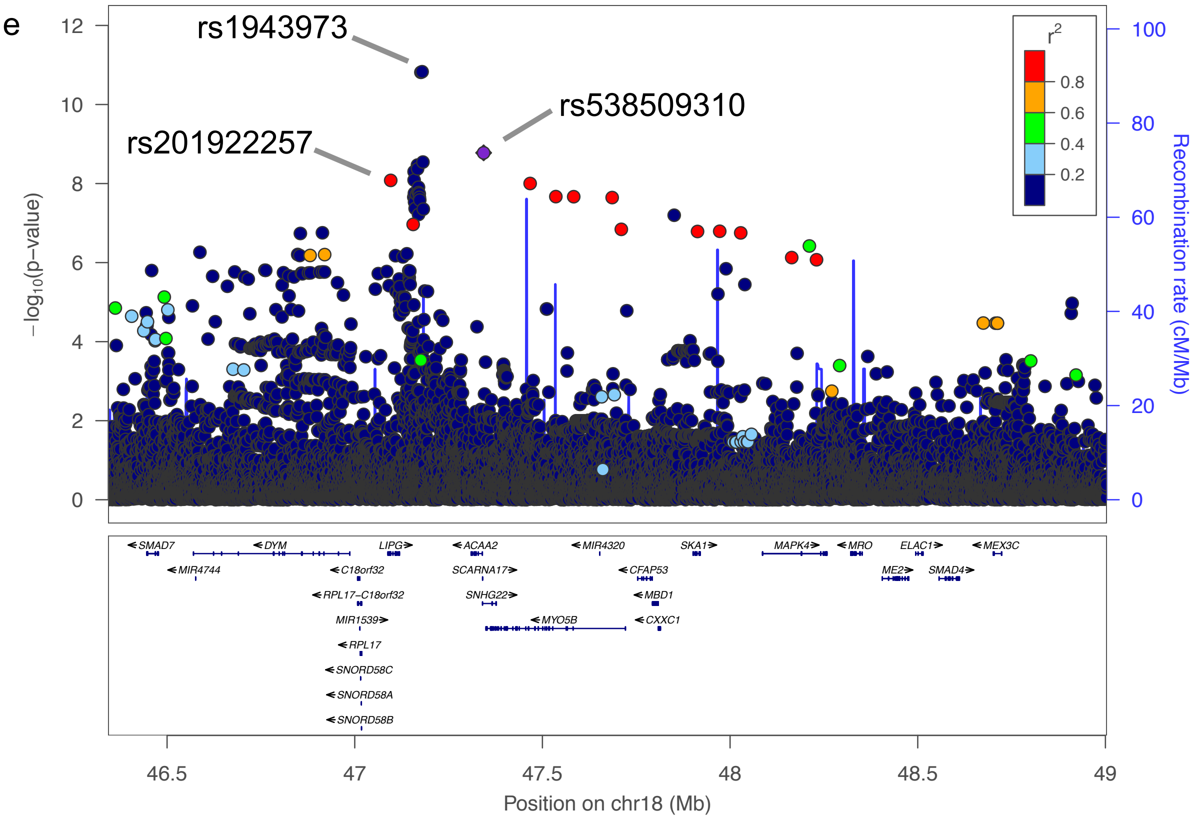

S5 Fig. Unconditional plots of the five lipid and lipoprotein subclass-associated loci. Each circle represents a single variant. The color is based on LD (r^2) between each variant and the reference variant (purple diamond); X-axis, genomic (GRCh37/hg19) position in Mb; Left y-axis, p-value of variant-trait association in $-\log_{10}$; Right y-axis, local estimates of genomic recombination rate in cM/Mb, represented by blue vertical lines.
