# Supplementary material for: Common, low-frequency, and rare genetic variants associated with lipoprotein subclasses and triglyceride measures in Finnish men from the METSIM study
Source: PLoS Genet. 2017 Oct 30;13(10):e1007079. doi: 10.1371/journal.pgen.1007079 (PMC5679656; doi:10.1371/journal.pgen.1007079)
Supplement: S6 Fig — The Pvalue is shown in -log10 and in the direction (+ or −) of the effect (Beta). The red line denotes the significance cutoff of P≤5E-8. The red asterisk indicates the most significantly associated trait. CAD, coronary artery disease. (PDF) [file pgen.1007079.s006.pdf]

S6 Fig. Association of all 72 lipoprotein/lipid traits with the variants in Table 1. The *P*value is shown in -log10 and in the direction (+ or −) of the effect (Beta). The red line denotes the significance cutoff of P≤5E-8. The red asterisk indicates the most significantly associated trait. CAD, coronary artery disease.

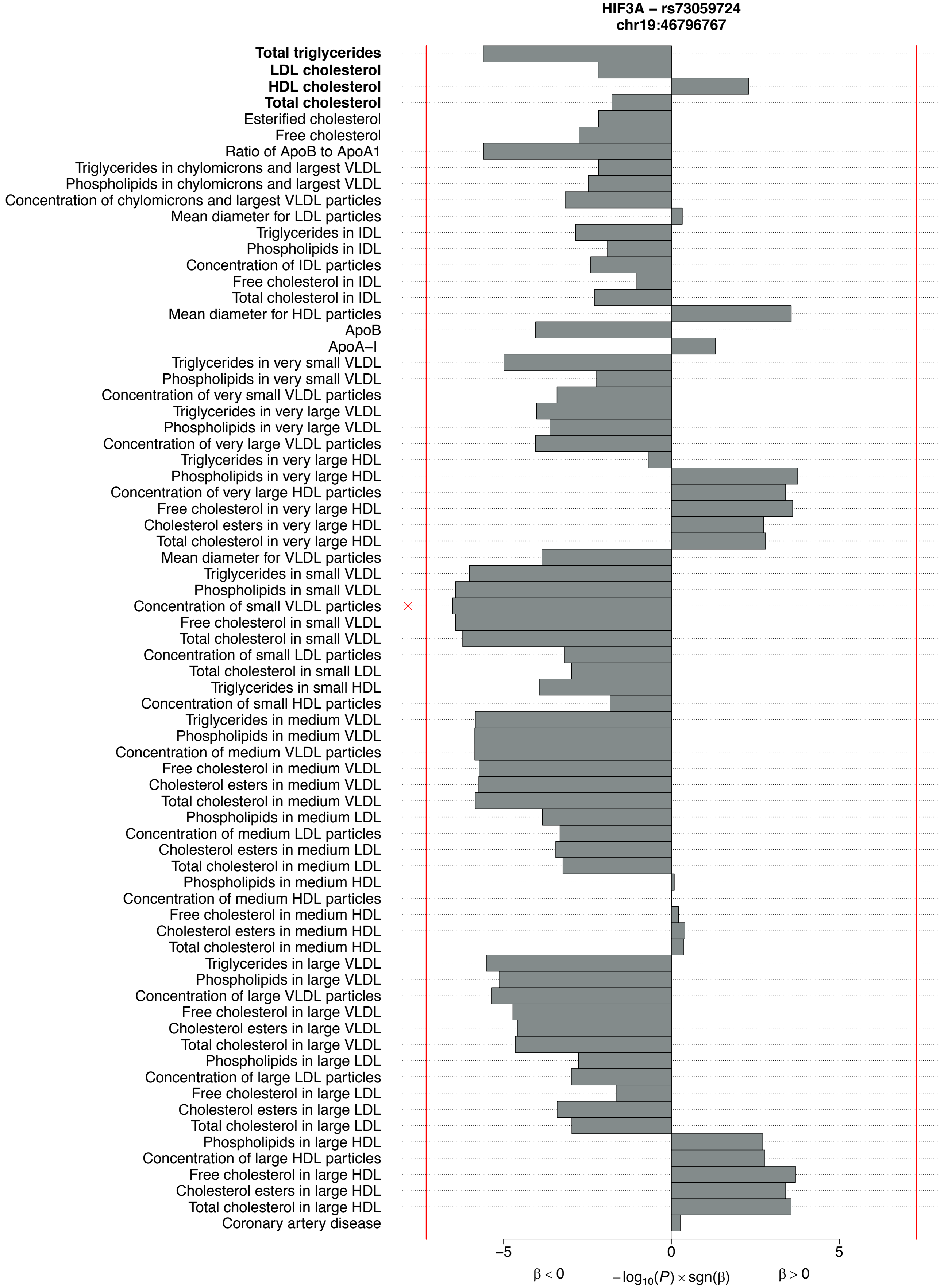

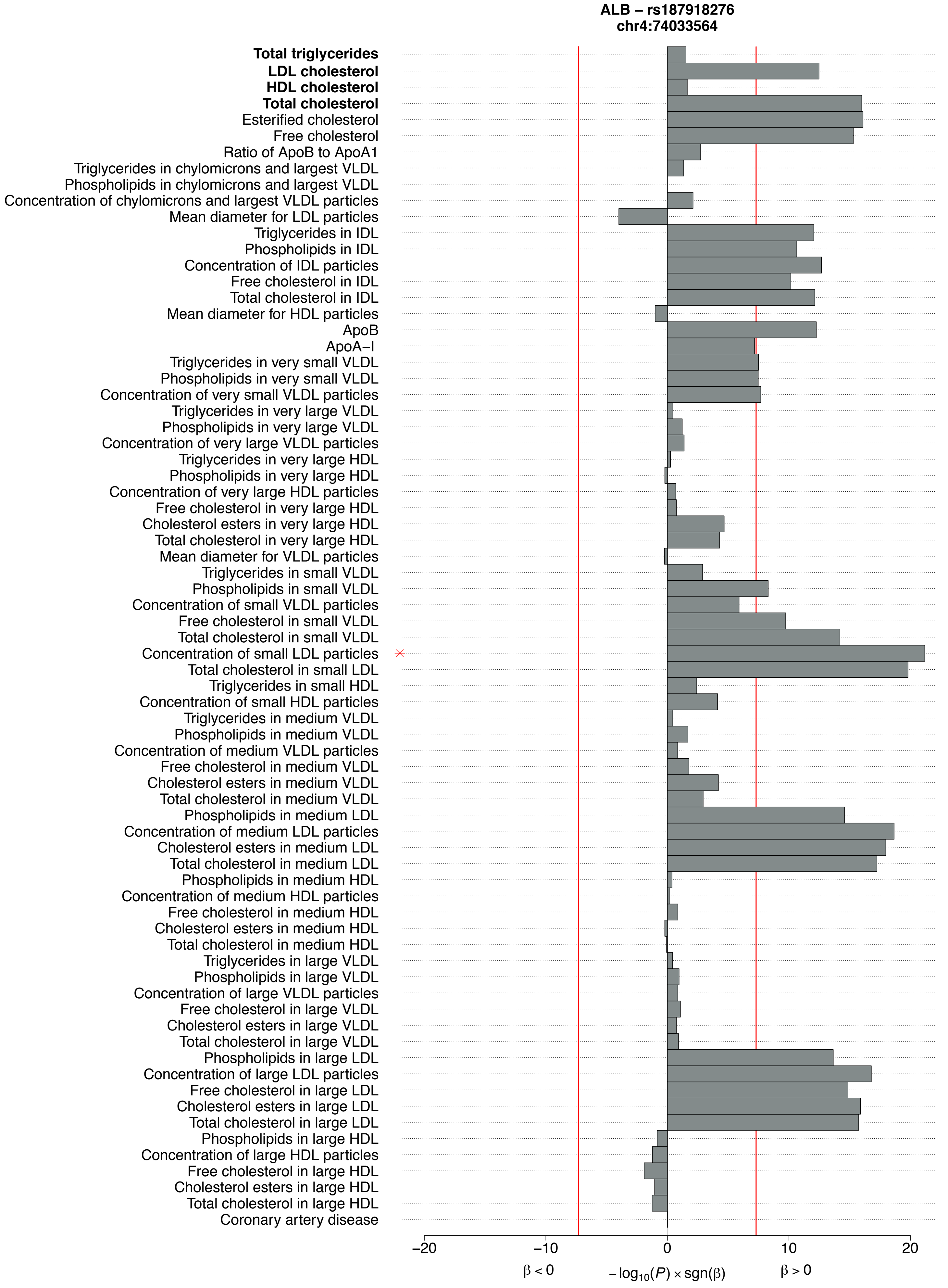
ALB – rs187918276
chr4:74033564
Total triglycerides
LDL cholesterol
HDL cholesterol
Total cholesterol
Esterified cholesterol
Free cholesterol
Ratio of ApoB to ApoA1
Triglycerides in chylomicrons and largest VLDL
Phospholipids in chylomicrons and largest VLDL
Concentration of chylomicrons and largest VLDL particles
Mean diameter for LDL particles
Triglycerides in IDL
Phospholipids in IDL
Concentration of IDL particles
Free cholesterol in IDL
Total cholesterol in IDL
Mean diameter for HDL particles
ApoB
ApoA−I
Triglycerides in very small VLDL
Phospholipids in very small VLDL
Concentration of very small VLDL particles
Triglycerides in very large VLDL
Phospholipids in very large VLDL
Concentration of very large VLDL particles
Triglycerides in very large HDL
Phospholipids in very large HDL
Concentration of very large HDL particles
Free cholesterol in very large HDL
Cholesterol esters in very large HDL
Total cholesterol in very large HDL
Mean diameter for VLDL particles
Triglycerides in small VLDL
Phospholipids in small VLDL
Concentration of small VLDL particles
Free cholesterol in small VLDL
Total cholesterol in small VLDL
Concentration of small LDL particles
Total cholesterol in small LDL
Triglycerides in small HDL
Concentration of small HDL particles
Triglycerides in medium VLDL
Phospholipids in medium VLDL
Concentration of medium VLDL particles
Free cholesterol in medium VLDL
Cholesterol esters in medium VLDL
Total cholesterol in medium VLDL
Phospholipids in medium LDL
Concentration of medium LDL particles
Cholesterol esters in medium LDL
Total cholesterol in medium LDL
Phospholipids in medium HDL
Concentration of medium HDL particles
Free cholesterol in medium HDL
Cholesterol esters in medium HDL
Total cholesterol in medium HDL
Triglycerides in large VLDL
Phospholipids in large VLDL
Concentration of large VLDL particles
Free cholesterol in large VLDL
Cholesterol esters in large VLDL
Total cholesterol in large VLDL
Phospholipids in large LDL
Concentration of large LDL particles
Free cholesterol in large LDL
Cholesterol esters in large LDL
Total cholesterol in large LDL
Phospholipids in large HDL
Concentration of large HDL particles
Free cholesterol in large HDL
Cholesterol esters in large HDL
Total cholesterol in large HDL
Coronary artery disease
−20
−10
0
10
20
β < 0
− log10(P) × sgn(β)
β > 0

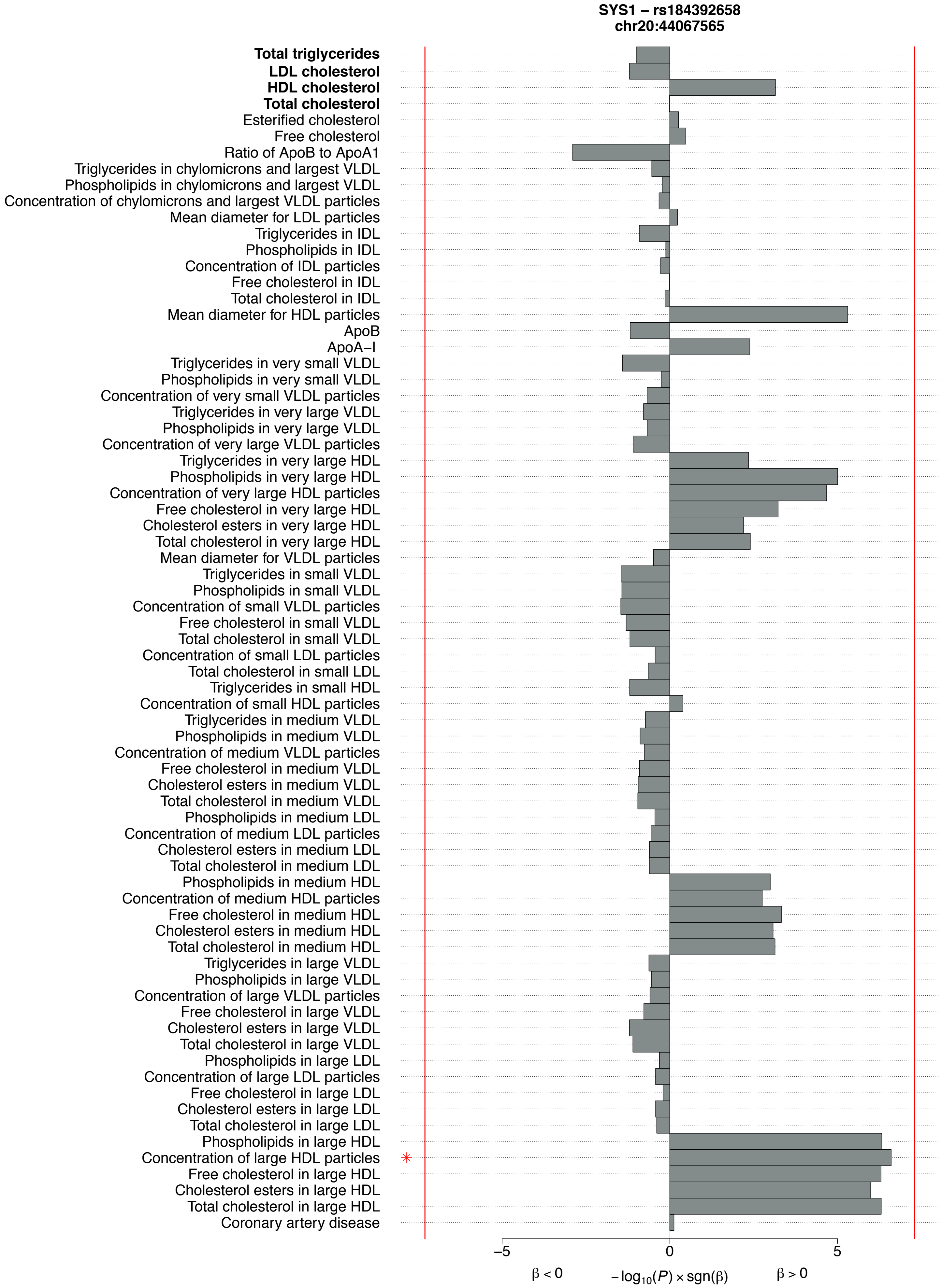
SYS1 – rs184392658
chr20:44067565
Total triglycerides
LDL cholesterol
HDL cholesterol
Total cholesterol
Esterified cholesterol
Free cholesterol
Ratio of ApoB to ApoA1
Triglycerides in chylomicrons and largest VLDL
Phospholipids in chylomicrons and largest VLDL
Concentration of chylomicrons and largest VLDL particles
Mean diameter for LDL particles
Triglycerides in IDL
Phospholipids in IDL
Concentration of IDL particles
Free cholesterol in IDL
Total cholesterol in IDL
Mean diameter for HDL particles
ApoB
ApoA–I
Triglycerides in very small VLDL
Phospholipids in very small VLDL
Concentration of very small VLDL particles
Triglycerides in very large VLDL
Phospholipids in very large VLDL
Concentration of very large VLDL particles
Triglycerides in very large HDL
Phospholipids in very large HDL
Concentration of very large HDL particles
Free cholesterol in very large HDL
Cholesterol esters in very large HDL
Total cholesterol in very large HDL
Mean diameter for VLDL particles
Triglycerides in small VLDL
Phospholipids in small VLDL
Concentration of small VLDL particles
Free cholesterol in small VLDL
Total cholesterol in small VLDL
Concentration of small LDL particles
Total cholesterol in small LDL
Triglycerides in small HDL
Concentration of small HDL particles
Triglycerides in medium VLDL
Phospholipids in medium VLDL
Concentration of medium VLDL particles
Free cholesterol in medium VLDL
Cholesterol esters in medium VLDL
Total cholesterol in medium VLDL
Phospholipids in medium LDL
Concentration of medium LDL particles
Cholesterol esters in medium LDL
Total cholesterol in medium LDL
Phospholipids in medium HDL
Concentration of medium HDL particles
Free cholesterol in medium HDL
Cholesterol esters in medium HDL
Total cholesterol in medium HDL
Triglycerides in large VLDL
Phospholipids in large VLDL
Concentration of large VLDL particles
Free cholesterol in large VLDL
Cholesterol esters in large VLDL
Total cholesterol in large VLDL
Phospholipids in large LDL
Concentration of large LDL particles
Free cholesterol in large LDL
Cholesterol esters in large LDL
Total cholesterol in large LDL
Phospholipids in large HDL
Concentration of large HDL particles
Free cholesterol in large HDL
Cholesterol esters in large HDL
Total cholesterol in large HDL
Coronary artery disease
–5
0
5
β < 0
$-\log_{10}(P) \times \mathrm{sgn}(\beta)$
β > 0

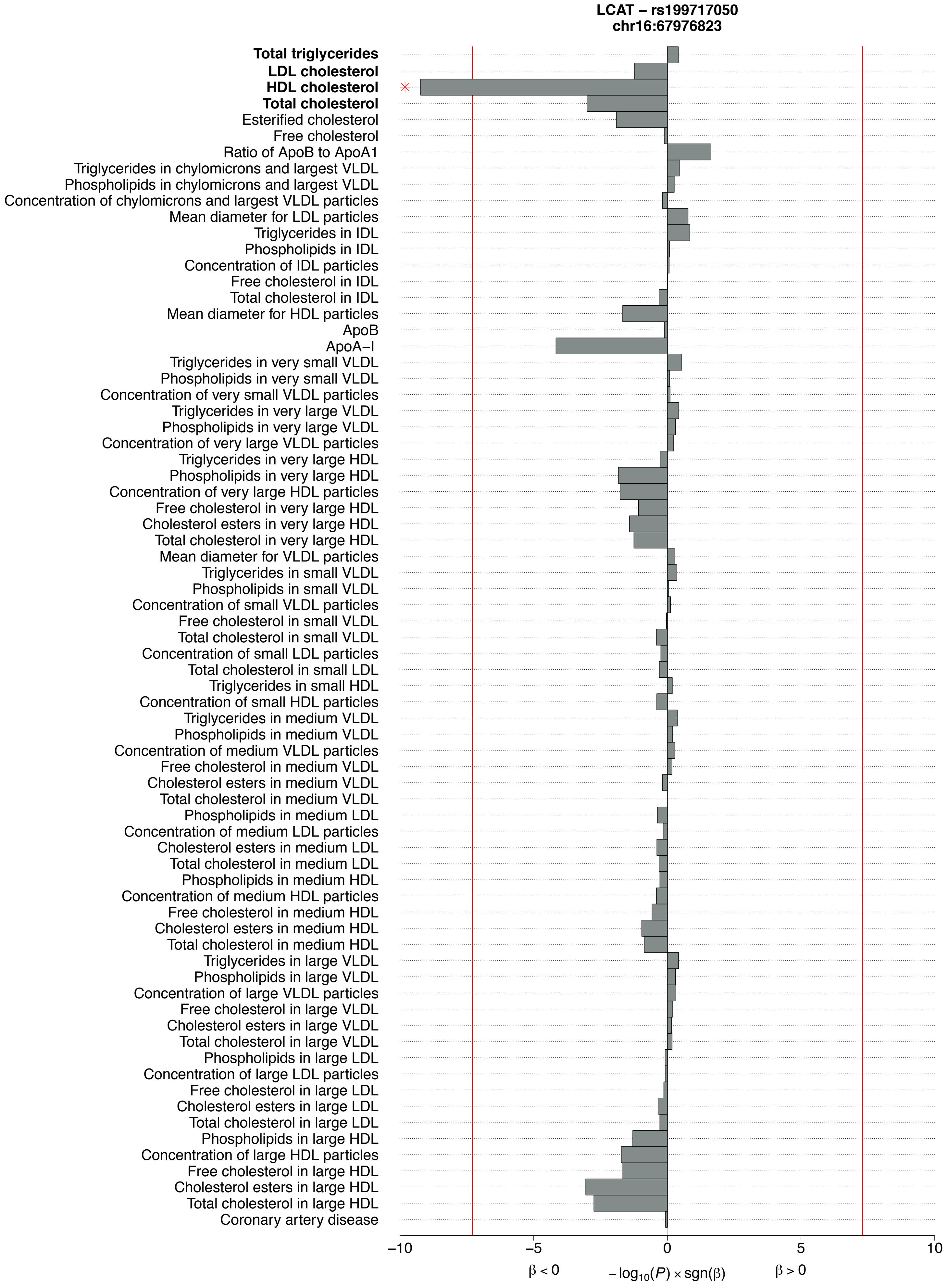
LCAT – rs199717050
chr16:67976823
Total triglycerides
LDL cholesterol
HDL cholesterol
Total cholesterol
Esterified cholesterol
Free cholesterol
Ratio of ApoB to ApoA1
Triglycerides in chylomicrons and largest VLDL
Phospholipids in chylomicrons and largest VLDL
Concentration of chylomicrons and largest VLDL particles
Mean diameter for LDL particles
Triglycerides in IDL
Phospholipids in IDL
Concentration of IDL particles
Free cholesterol in IDL
Total cholesterol in IDL
Mean diameter for HDL particles
ApoB
ApoA–I
Triglycerides in very small VLDL
Phospholipids in very small VLDL
Concentration of very small VLDL particles
Triglycerides in very large VLDL
Phospholipids in very large VLDL
Concentration of very large VLDL particles
Triglycerides in very large HDL
Phospholipids in very large HDL
Concentration of very large HDL particles
Free cholesterol in very large HDL
Cholesterol esters in very large HDL
Total cholesterol in very large HDL
Mean diameter for VLDL particles
Triglycerides in small VLDL
Phospholipids in small VLDL
Concentration of small VLDL particles
Free cholesterol in small VLDL
Total cholesterol in small VLDL
Concentration of small LDL particles
Total cholesterol in small LDL
Triglycerides in small HDL
Concentration of small HDL particles
Triglycerides in medium VLDL
Phospholipids in medium VLDL
Concentration of medium VLDL particles
Free cholesterol in medium VLDL
Cholesterol esters in medium VLDL
Total cholesterol in medium VLDL
Phospholipids in medium LDL
Concentration of medium LDL particles
Cholesterol esters in medium LDL
Total cholesterol in medium LDL
Phospholipids in medium HDL
Concentration of medium HDL particles
Free cholesterol in medium HDL
Cholesterol esters in medium HDL
Total cholesterol in medium HDL
Triglycerides in large VLDL
Phospholipids in large VLDL
Concentration of large VLDL particles
Free cholesterol in large VLDL
Cholesterol esters in large VLDL
Total cholesterol in large VLDL
Phospholipids in large LDL
Concentration of large LDL particles
Free cholesterol in large LDL
Cholesterol esters in large LDL
Total cholesterol in large LDL
Phospholipids in large HDL
Concentration of large HDL particles
Free cholesterol in large HDL
Cholesterol esters in large HDL
Total cholesterol in large HDL
Coronary artery disease
−10
−5
0
5
10
β < 0
−log10(P) × sgn(β)
β > 0

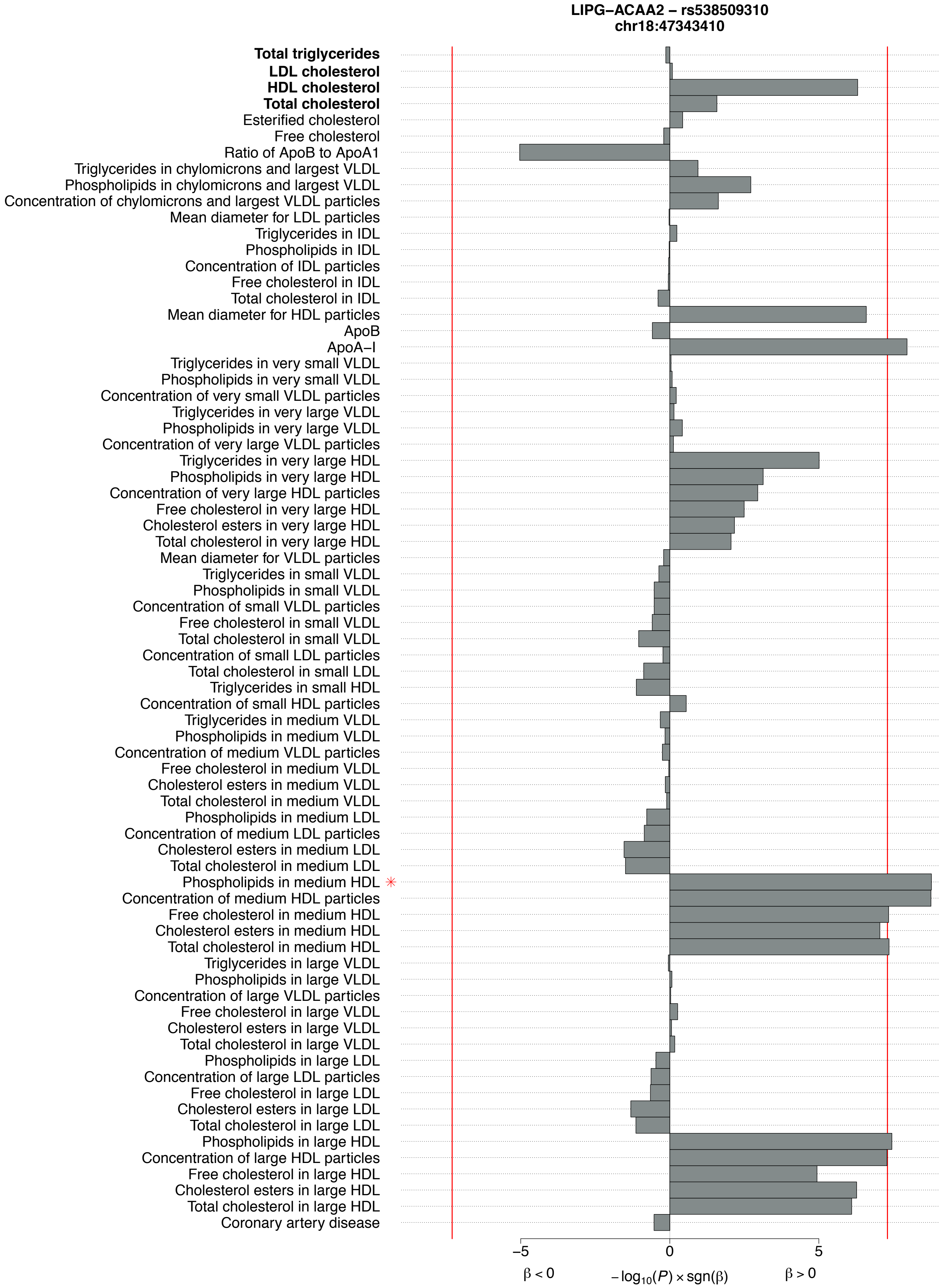

LIPG−ACAA2 − rs538509310
chr18:47343410
Total triglycerides
LDL cholesterol
HDL cholesterol
Total cholesterol
Esterified cholesterol
Free cholesterol
Ratio of ApoB to ApoA1
Triglycerides in chylomicrons and largest VLDL
Phospholipids in chylomicrons and largest VLDL
Concentration of chylomicrons and largest VLDL particles
Mean diameter for LDL particles
Triglycerides in IDL
Phospholipids in IDL
Concentration of IDL particles
Free cholesterol in IDL
Total cholesterol in IDL
Mean diameter for HDL particles
ApoB
ApoA−I
Triglycerides in very small VLDL
Phospholipids in very small VLDL
Concentration of very small VLDL particles
Triglycerides in very large VLDL
Phospholipids in very large VLDL
Concentration of very large VLDL particles
Triglycerides in very large HDL
Phospholipids in very large HDL
Concentration of very large HDL particles
Free cholesterol in very large HDL
Cholesterol esters in very large HDL
Total cholesterol in very large HDL
Mean diameter for VLDL particles
Triglycerides in small VLDL
Phospholipids in small VLDL
Concentration of small VLDL particles
Free cholesterol in small VLDL
Total cholesterol in small VLDL
Concentration of small LDL particles
Total cholesterol in small LDL
Triglycerides in small HDL
Concentration of small HDL particles
Triglycerides in medium VLDL
Phospholipids in medium VLDL
Concentration of medium VLDL particles
Free cholesterol in medium VLDL
Cholesterol esters in medium VLDL
Total cholesterol in medium VLDL
Phospholipids in medium LDL
Concentration of medium LDL particles
Cholesterol esters in medium LDL
Total cholesterol in medium LDL
Phospholipids in medium HDL *
Concentration of medium HDL particles
Free cholesterol in medium HDL
Cholesterol esters in medium HDL
Total cholesterol in medium HDL
Triglycerides in large VLDL
Phospholipids in large VLDL
Concentration of large VLDL particles
Free cholesterol in large VLDL
Cholesterol esters in large VLDL
Total cholesterol in large VLDL
Phospholipids in large LDL
Concentration of large LDL particles
Free cholesterol in large LDL
Cholesterol esters in large LDL
Total cholesterol in large LDL
Phospholipids in large HDL
Concentration of large HDL particles
Free cholesterol in large HDL
Cholesterol esters in large HDL
Total cholesterol in large HDL
Coronary artery disease
−5
0
5
β < 0
$-\log_{10}(P) \times \mathrm{sgn}(\beta)$
β > 0
