# Supplementary material for: Common, low-frequency, and rare genetic variants associated with lipoprotein subclasses and triglyceride measures in Finnish men from the METSIM study
Source: PLoS Genet. 2017 Oct 30;13(10):e1007079. doi: 10.1371/journal.pgen.1007079 (PMC5679656; doi:10.1371/journal.pgen.1007079)
Supplement: S7 Fig — The Pvalue is shown in -log10 and in the direction (+ or −) of the effect (Beta). The red line denotes the significance cutoff of P≤5E-8. The red asterisk indicates the most significantly associated trait. CAD, coronary artery disease. (PDF) [file pgen.1007079.s007.pdf]

S7 Fig. Association of all 72 lipoprotein/lipid traits with the variants in Table 2. The *P* value is shown in -log10 and in the direction (+ or −) of the effect (Beta). The red line denotes the significance cutoff of P≤5E-8. The red asterisk indicates the most significantly associated trait. CAD, coronary artery disease.

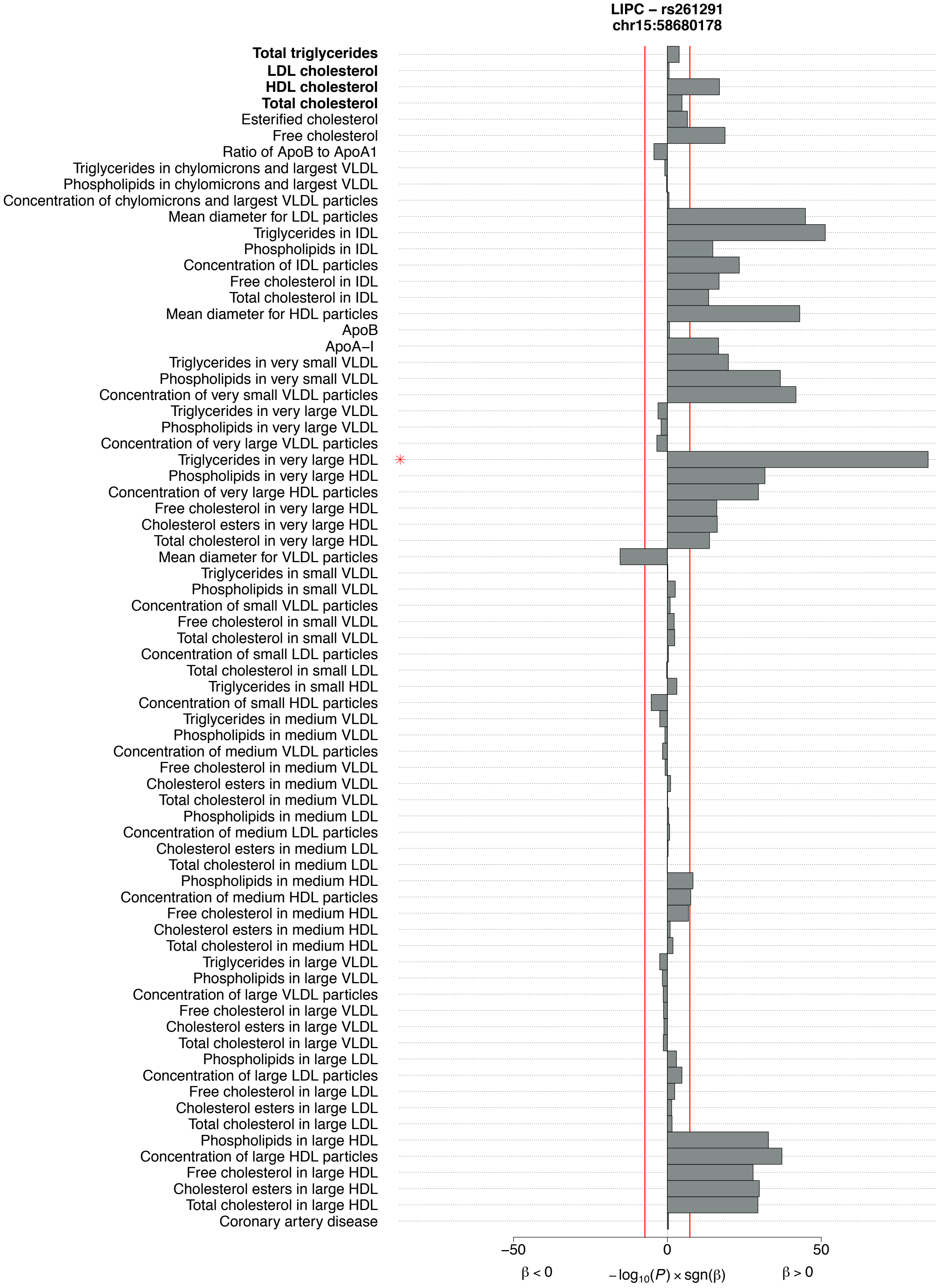

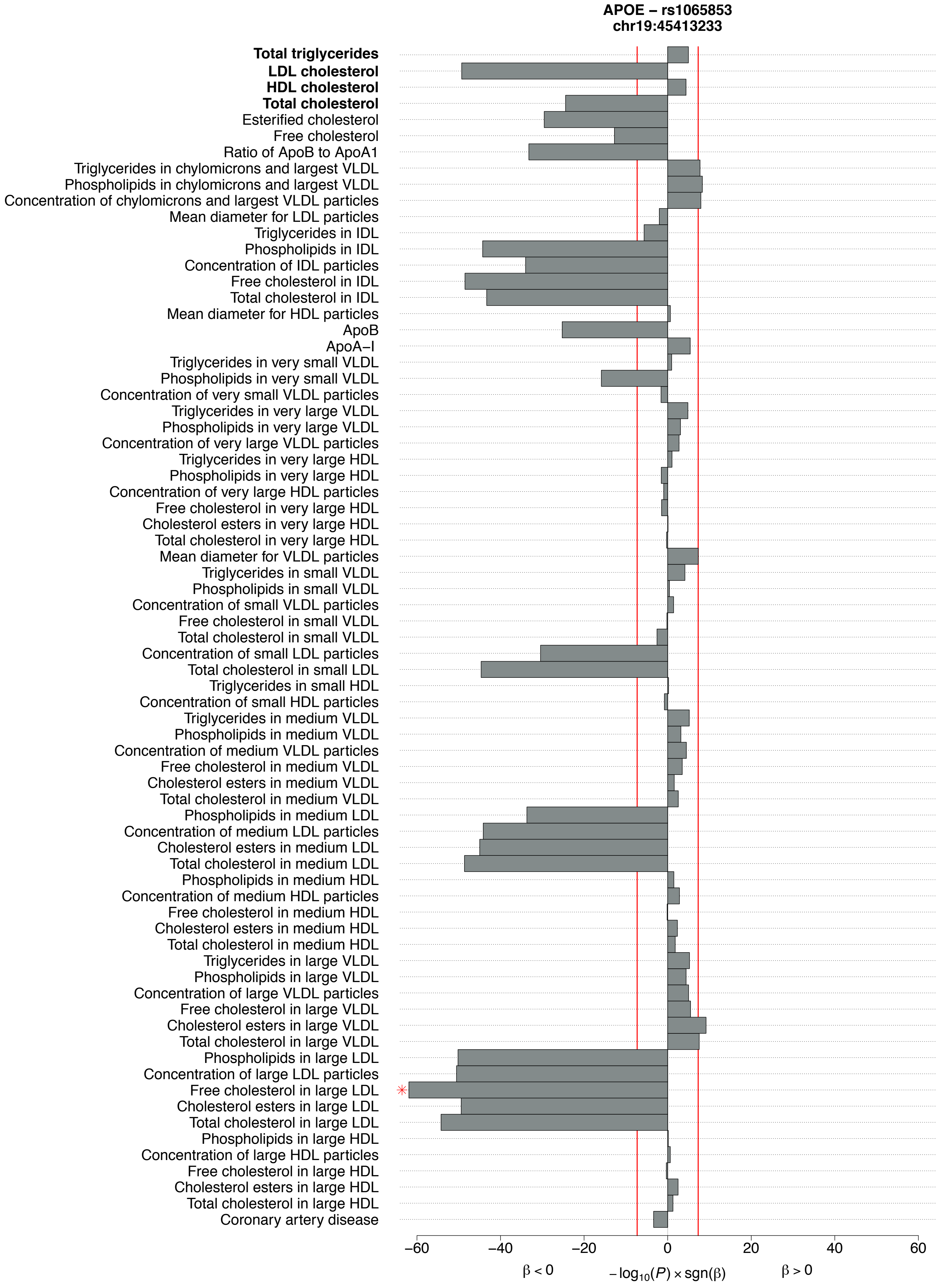
APOE – rs1065853
chr19:45413233
Total triglycerides
LDL cholesterol
HDL cholesterol
Total cholesterol
Esterified cholesterol
Free cholesterol
Ratio of ApoB to ApoA1
Triglycerides in chylomicrons and largest VLDL
Phospholipids in chylomicrons and largest VLDL
Concentration of chylomicrons and largest VLDL particles
Mean diameter for LDL particles
Triglycerides in IDL
Phospholipids in IDL
Concentration of IDL particles
Free cholesterol in IDL
Total cholesterol in IDL
Mean diameter for HDL particles
ApoB
ApoA−I
Triglycerides in very small VLDL
Phospholipids in very small VLDL
Concentration of very small VLDL particles
Triglycerides in very large VLDL
Phospholipids in very large VLDL
Concentration of very large VLDL particles
Triglycerides in very large HDL
Phospholipids in very large HDL
Concentration of very large HDL particles
Free cholesterol in very large HDL
Cholesterol esters in very large HDL
Total cholesterol in very large HDL
Mean diameter for VLDL particles
Triglycerides in small VLDL
Phospholipids in small VLDL
Concentration of small VLDL particles
Free cholesterol in small VLDL
Total cholesterol in small VLDL
Concentration of small LDL particles
Total cholesterol in small LDL
Triglycerides in small HDL
Concentration of small HDL particles
Triglycerides in medium VLDL
Phospholipids in medium VLDL
Concentration of medium VLDL particles
Free cholesterol in medium VLDL
Cholesterol esters in medium VLDL
Total cholesterol in medium VLDL
Phospholipids in medium LDL
Concentration of medium LDL particles
Cholesterol esters in medium LDL
Total cholesterol in medium LDL
Phospholipids in medium HDL
Concentration of medium HDL particles
Free cholesterol in medium HDL
Cholesterol esters in medium HDL
Total cholesterol in medium HDL
Triglycerides in large VLDL
Phospholipids in large VLDL
Concentration of large VLDL particles
Free cholesterol in large VLDL
Cholesterol esters in large VLDL
Total cholesterol in large VLDL
Phospholipids in large LDL
Concentration of large LDL particles
Free cholesterol in large LDL
Cholesterol esters in large LDL
Total cholesterol in large LDL
Phospholipids in large HDL
Concentration of large HDL particles
Free cholesterol in large HDL
Cholesterol esters in large HDL
Total cholesterol in large HDL
Coronary artery disease
−60
−40
−20
0
20
40
60
β < 0
−log10(P) × sgn(β)
β > 0

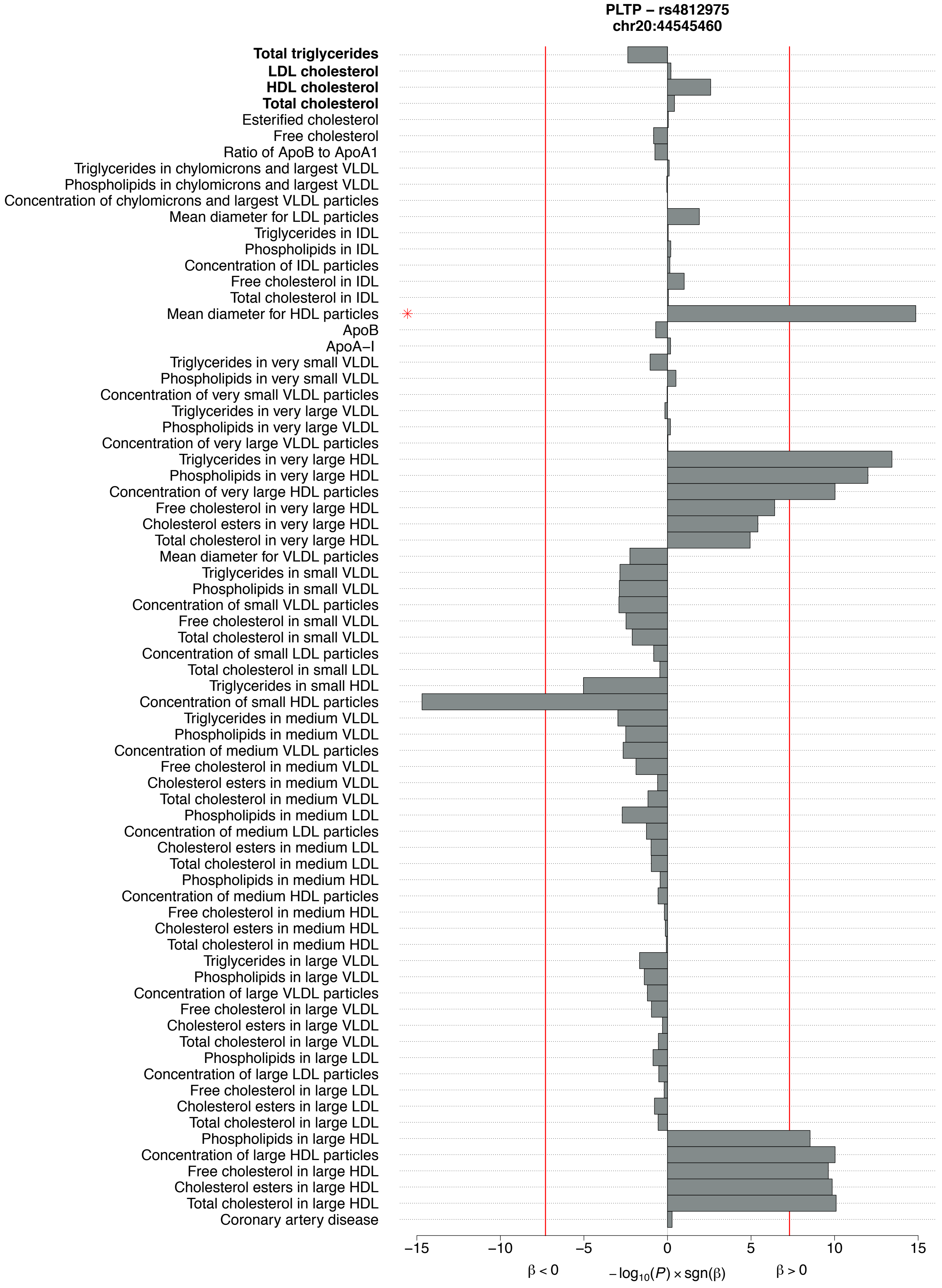

PLTP – rs4812975
chr20:44545460
Total triglycerides
LDL cholesterol
HDL cholesterol
Total cholesterol
Esterified cholesterol
Free cholesterol
Ratio of ApoB to ApoA1
Triglycerides in chylomicrons and largest VLDL
Phospholipids in chylomicrons and largest VLDL
Concentration of chylomicrons and largest VLDL particles
Mean diameter for LDL particles
Triglycerides in IDL
Phospholipids in IDL
Concentration of IDL particles
Free cholesterol in IDL
Total cholesterol in IDL
Mean diameter for HDL particles
ApoB
ApoA–I
Triglycerides in very small VLDL
Phospholipids in very small VLDL
Concentration of very small VLDL particles
Triglycerides in very large VLDL
Phospholipids in very large VLDL
Concentration of very large VLDL particles
Triglycerides in very large HDL
Phospholipids in very large HDL
Concentration of very large HDL particles
Free cholesterol in very large HDL
Cholesterol esters in very large HDL
Total cholesterol in very large HDL
Mean diameter for VLDL particles
Triglycerides in small VLDL
Phospholipids in small VLDL
Concentration of small VLDL particles
Free cholesterol in small VLDL
Total cholesterol in small VLDL
Concentration of small LDL particles
Total cholesterol in small LDL
Triglycerides in small HDL
Concentration of small HDL particles
Triglycerides in medium VLDL
Phospholipids in medium VLDL
Concentration of medium VLDL particles
Free cholesterol in medium VLDL
Cholesterol esters in medium VLDL
Total cholesterol in medium VLDL
Phospholipids in medium LDL
Concentration of medium LDL particles
Cholesterol esters in medium LDL
Total cholesterol in medium LDL
Phospholipids in medium HDL
Concentration of medium HDL particles
Free cholesterol in medium HDL
Cholesterol esters in medium HDL
Total cholesterol in medium HDL
Triglycerides in large VLDL
Phospholipids in large VLDL
Concentration of large VLDL particles
Free cholesterol in large VLDL
Cholesterol esters in large VLDL
Total cholesterol in large VLDL
Phospholipids in large LDL
Concentration of large LDL particles
Free cholesterol in large LDL
Cholesterol esters in large LDL
Total cholesterol in large LDL
Phospholipids in large HDL
Concentration of large HDL particles
Free cholesterol in large HDL
Cholesterol esters in large HDL
Total cholesterol in large HDL
Coronary artery disease
−15
−10
−5
0
5
10
15
β < 0
− log10(P) × sgn(β)
β > 0

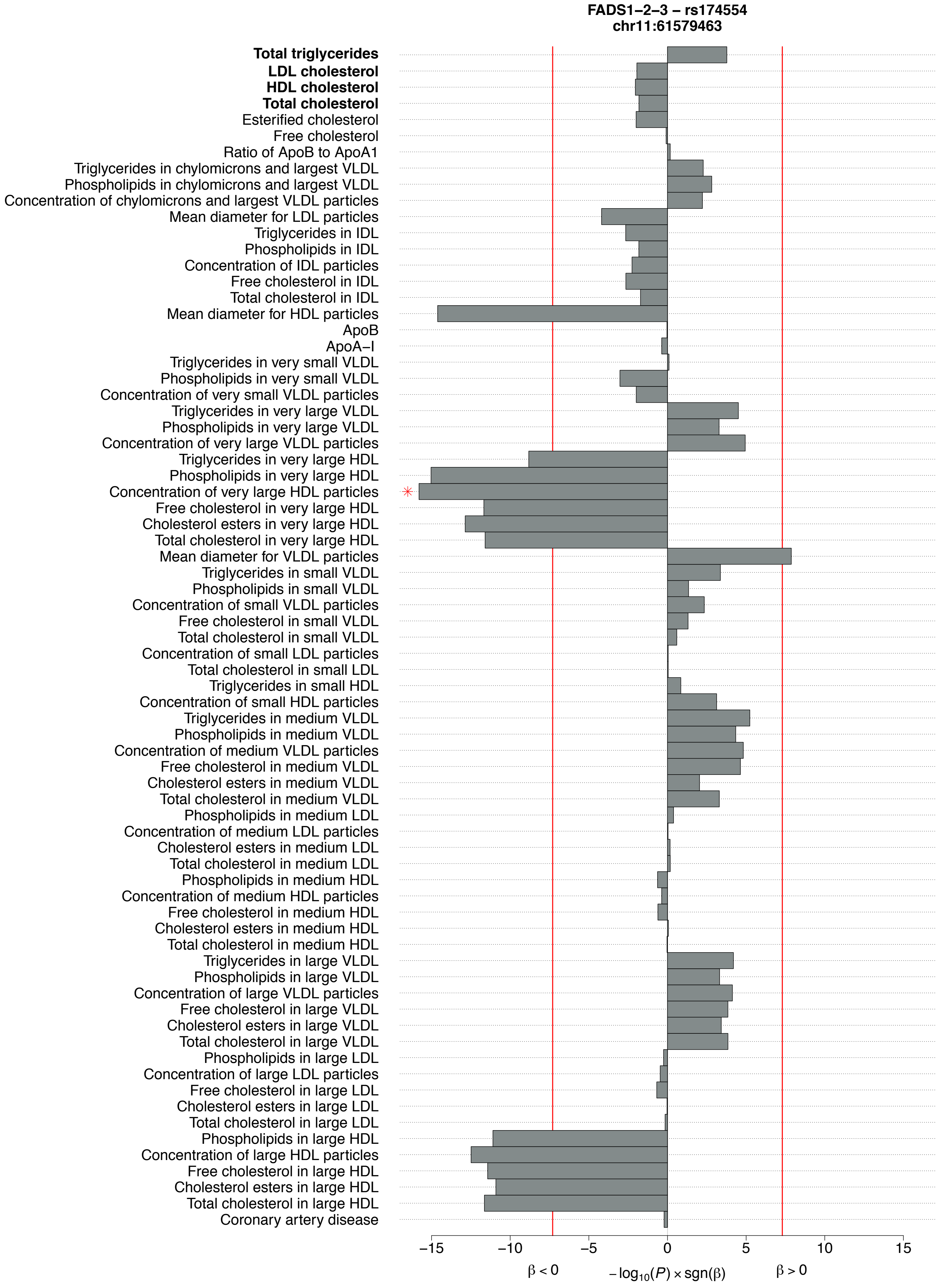
FADS1–2–3 – rs174554
chr11:61579463
Total triglycerides
LDL cholesterol
HDL cholesterol
Total cholesterol
Esterified cholesterol
Free cholesterol
Ratio of ApoB to ApoA1
Triglycerides in chylomicrons and largest VLDL
Phospholipids in chylomicrons and largest VLDL
Concentration of chylomicrons and largest VLDL particles
Mean diameter for LDL particles
Triglycerides in IDL
Phospholipids in IDL
Concentration of IDL particles
Free cholesterol in IDL
Total cholesterol in IDL
Mean diameter for HDL particles
ApoB
ApoA–I
Triglycerides in very small VLDL
Phospholipids in very small VLDL
Concentration of very small VLDL particles
Triglycerides in very large VLDL
Phospholipids in very large VLDL
Concentration of very large VLDL particles
Triglycerides in very large HDL
Phospholipids in very large HDL
Concentration of very large HDL particles
Free cholesterol in very large HDL
Cholesterol esters in very large HDL
Total cholesterol in very large HDL
Mean diameter for VLDL particles
Triglycerides in small VLDL
Phospholipids in small VLDL
Concentration of small VLDL particles
Free cholesterol in small VLDL
Total cholesterol in small VLDL
Concentration of small LDL particles
Total cholesterol in small LDL
Triglycerides in small HDL
Concentration of small HDL particles
Triglycerides in medium VLDL
Phospholipids in medium VLDL
Concentration of medium VLDL particles
Free cholesterol in medium VLDL
Cholesterol esters in medium VLDL
Total cholesterol in medium VLDL
Phospholipids in medium LDL
Concentration of medium LDL particles
Cholesterol esters in medium LDL
Total cholesterol in medium LDL
Phospholipids in medium HDL
Concentration of medium HDL particles
Free cholesterol in medium HDL
Cholesterol esters in medium HDL
Total cholesterol in medium HDL
Triglycerides in large VLDL
Phospholipids in large VLDL
Concentration of large VLDL particles
Free cholesterol in large VLDL
Cholesterol esters in large VLDL
Total cholesterol in large VLDL
Phospholipids in large LDL
Concentration of large LDL particles
Free cholesterol in large LDL
Cholesterol esters in large LDL
Total cholesterol in large LDL
Phospholipids in large HDL
Concentration of large HDL particles
Free cholesterol in large HDL
Cholesterol esters in large HDL
Total cholesterol in large HDL
Coronary artery disease
–15
–10
–5
0
5
10
15
β < 0
$-\log_{10}(P) \times \text{sgn}(\beta)$
β > 0

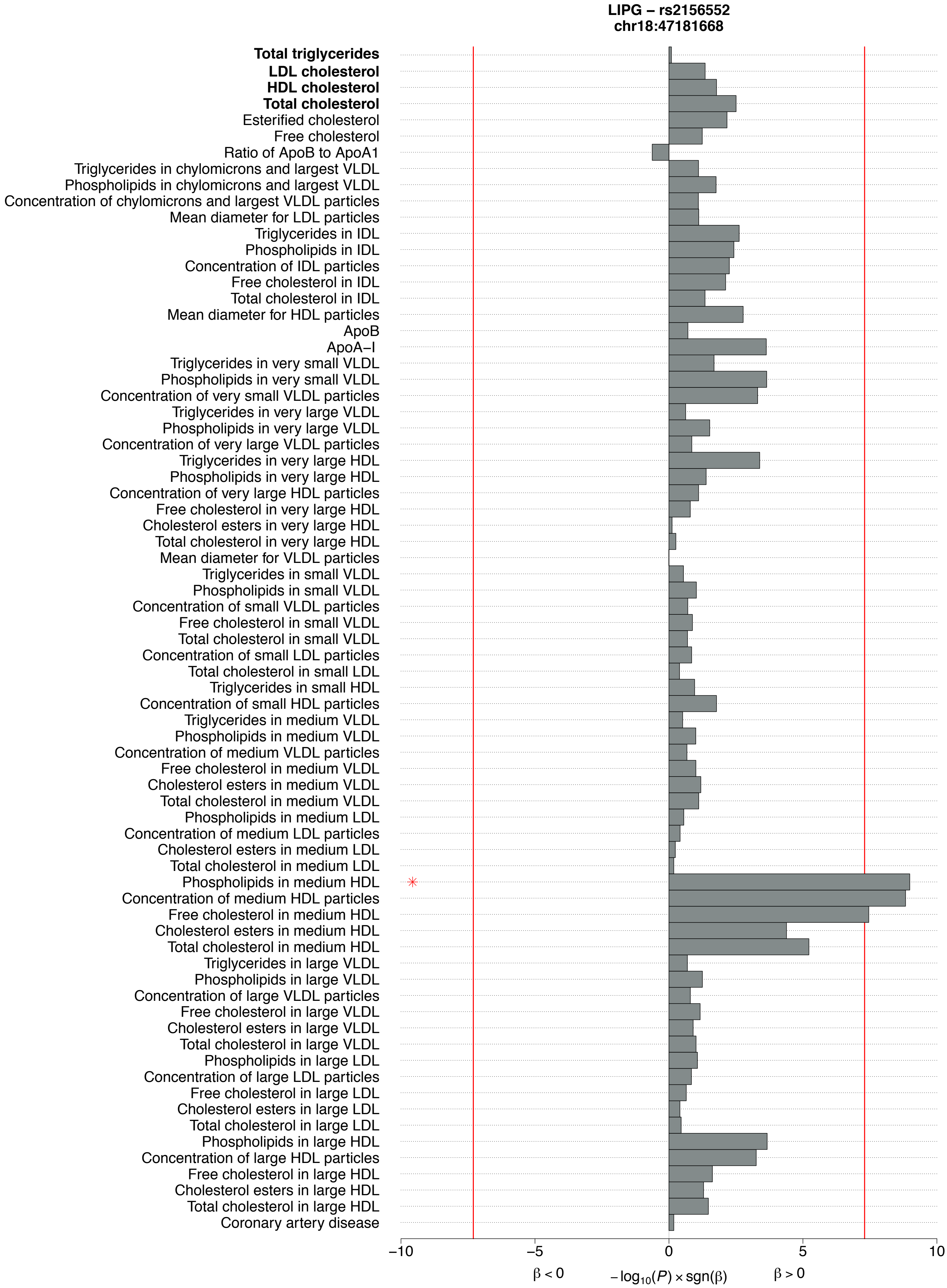
LIPG – rs2156552
chr18:47181668
Total triglycerides
LDL cholesterol
HDL cholesterol
Total cholesterol
Esterified cholesterol
Free cholesterol
Ratio of ApoB to ApoA1
Triglycerides in chylomicrons and largest VLDL
Phospholipids in chylomicrons and largest VLDL
Concentration of chylomicrons and largest VLDL particles
Mean diameter for LDL particles
Triglycerides in IDL
Phospholipids in IDL
Concentration of IDL particles
Free cholesterol in IDL
Total cholesterol in IDL
Mean diameter for HDL particles
ApoB
ApoA–I
Triglycerides in very small VLDL
Phospholipids in very small VLDL
Concentration of very small VLDL particles
Triglycerides in very large VLDL
Phospholipids in very large VLDL
Concentration of very large VLDL particles
Triglycerides in very large HDL
Phospholipids in very large HDL
Concentration of very large HDL particles
Free cholesterol in very large HDL
Cholesterol esters in very large HDL
Total cholesterol in very large HDL
Mean diameter for VLDL particles
Triglycerides in small VLDL
Phospholipids in small VLDL
Concentration of small VLDL particles
Free cholesterol in small VLDL
Total cholesterol in small VLDL
Concentration of small LDL particles
Total cholesterol in small LDL
Triglycerides in small HDL
Concentration of small HDL particles
Triglycerides in medium VLDL
Phospholipids in medium VLDL
Concentration of medium VLDL particles
Free cholesterol in medium VLDL
Cholesterol esters in medium VLDL
Total cholesterol in medium VLDL
Phospholipids in medium LDL
Concentration of medium LDL particles
Cholesterol esters in medium LDL
Total cholesterol in medium LDL
Phospholipids in medium HDL
Concentration of medium HDL particles
Free cholesterol in medium HDL
Cholesterol esters in medium HDL
Total cholesterol in medium HDL
Triglycerides in large VLDL
Phospholipids in large VLDL
Concentration of large VLDL particles
Free cholesterol in large VLDL
Cholesterol esters in large VLDL
Total cholesterol in large VLDL
Phospholipids in large LDL
Concentration of large LDL particles
Free cholesterol in large LDL
Cholesterol esters in large LDL
Total cholesterol in large LDL
Phospholipids in large HDL
Concentration of large HDL particles
Free cholesterol in large HDL
Cholesterol esters in large HDL
Total cholesterol in large HDL
Coronary artery disease
−10
−5
0
5
10
β < 0
− log10(P) × sgn(β)
β > 0

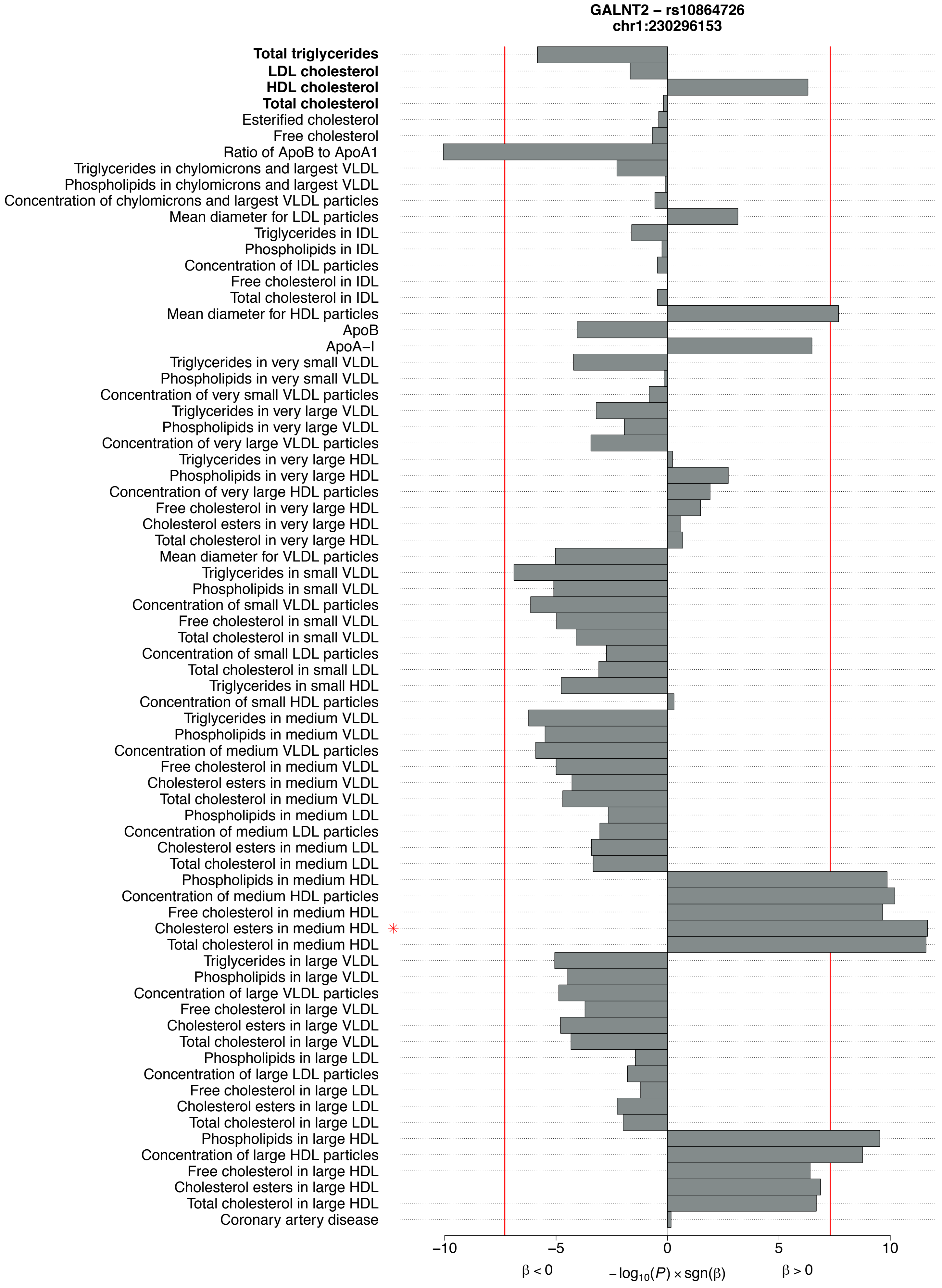
GALNT2 – rs10864726
chr1:230296153
Total triglycerides
LDL cholesterol
HDL cholesterol
Total cholesterol
Esterified cholesterol
Free cholesterol
Ratio of ApoB to ApoA1
Triglycerides in chylomicrons and largest VLDL
Phospholipids in chylomicrons and largest VLDL
Concentration of chylomicrons and largest VLDL particles
Mean diameter for LDL particles
Triglycerides in IDL
Phospholipids in IDL
Concentration of IDL particles
Free cholesterol in IDL
Total cholesterol in IDL
Mean diameter for HDL particles
ApoB
ApoA–I
Triglycerides in very small VLDL
Phospholipids in very small VLDL
Concentration of very small VLDL particles
Triglycerides in very large VLDL
Phospholipids in very large VLDL
Concentration of very large VLDL particles
Triglycerides in very large HDL
Phospholipids in very large HDL
Concentration of very large HDL particles
Free cholesterol in very large HDL
Cholesterol esters in very large HDL
Total cholesterol in very large HDL
Mean diameter for VLDL particles
Triglycerides in small VLDL
Phospholipids in small VLDL
Concentration of small VLDL particles
Free cholesterol in small VLDL
Total cholesterol in small VLDL
Concentration of small LDL particles
Total cholesterol in small LDL
Triglycerides in small HDL
Concentration of small HDL particles
Triglycerides in medium VLDL
Phospholipids in medium VLDL
Concentration of medium VLDL particles
Free cholesterol in medium VLDL
Cholesterol esters in medium VLDL
Total cholesterol in medium VLDL
Phospholipids in medium LDL
Concentration of medium LDL particles
Cholesterol esters in medium LDL
Total cholesterol in medium LDL
Phospholipids in medium HDL
Concentration of medium HDL particles
Free cholesterol in medium HDL
Cholesterol esters in medium HDL
Total cholesterol in medium HDL
Triglycerides in large VLDL
Phospholipids in large VLDL
Concentration of large VLDL particles
Free cholesterol in large VLDL
Cholesterol esters in large VLDL
Total cholesterol in large VLDL
Phospholipids in large LDL
Concentration of large LDL particles
Free cholesterol in large LDL
Cholesterol esters in large LDL
Total cholesterol in large LDL
Phospholipids in large HDL
Concentration of large HDL particles
Free cholesterol in large HDL
Cholesterol esters in large HDL
Total cholesterol in large HDL
Coronary artery disease
−10
−5
0
5
10
β < 0
−log10(P) × sgn(β)
β > 0

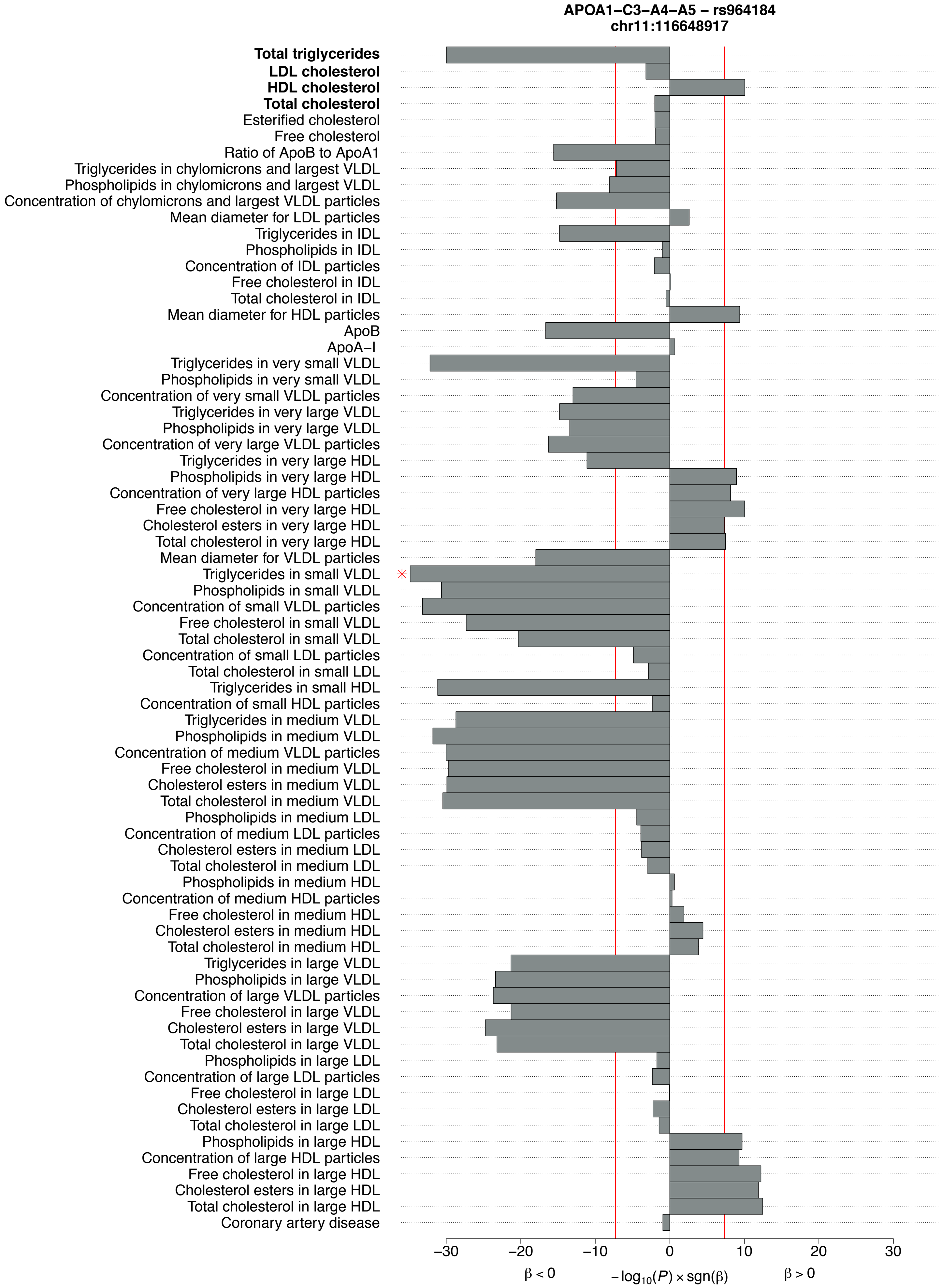
APOA1–C3–A4–A5 – rs964184
chr11:116648917
Total triglycerides
LDL cholesterol
HDL cholesterol
Total cholesterol
Esterified cholesterol
Free cholesterol
Ratio of ApoB to ApoA1
Triglycerides in chylomicrons and largest VLDL
Phospholipids in chylomicrons and largest VLDL
Concentration of chylomicrons and largest VLDL particles
Mean diameter for LDL particles
Triglycerides in IDL
Phospholipids in IDL
Concentration of IDL particles
Free cholesterol in IDL
Total cholesterol in IDL
Mean diameter for HDL particles
ApoB
ApoA–I
Triglycerides in very small VLDL
Phospholipids in very small VLDL
Concentration of very small VLDL particles
Triglycerides in very large VLDL
Phospholipids in very large VLDL
Concentration of very large VLDL particles
Triglycerides in very large HDL
Phospholipids in very large HDL
Concentration of very large HDL particles
Free cholesterol in very large HDL
Cholesterol esters in very large HDL
Total cholesterol in very large HDL
Mean diameter for VLDL particles
Triglycerides in small VLDL
Phospholipids in small VLDL
Concentration of small VLDL particles
Free cholesterol in small VLDL
Total cholesterol in small VLDL
Concentration of small LDL particles
Total cholesterol in small LDL
Triglycerides in small HDL
Concentration of small HDL particles
Triglycerides in medium VLDL
Phospholipids in medium VLDL
Concentration of medium VLDL particles
Free cholesterol in medium VLDL
Cholesterol esters in medium VLDL
Total cholesterol in medium VLDL
Phospholipids in medium LDL
Concentration of medium LDL particles
Cholesterol esters in medium LDL
Total cholesterol in medium LDL
Phospholipids in medium HDL
Concentration of medium HDL particles
Free cholesterol in medium HDL
Cholesterol esters in medium HDL
Total cholesterol in medium HDL
Triglycerides in large VLDL
Phospholipids in large VLDL
Concentration of large VLDL particles
Free cholesterol in large VLDL
Cholesterol esters in large VLDL
Total cholesterol in large VLDL
Phospholipids in large LDL
Concentration of large LDL particles
Free cholesterol in large LDL
Cholesterol esters in large LDL
Total cholesterol in large LDL
Phospholipids in large HDL
Concentration of large HDL particles
Free cholesterol in large HDL
Cholesterol esters in large HDL
Total cholesterol in large HDL
Coronary artery disease
−30
−20
−10
0
10
20
30
β < 0
$-\log_{10}(P) \times \mathrm{sgn}(\beta)$
β > 0

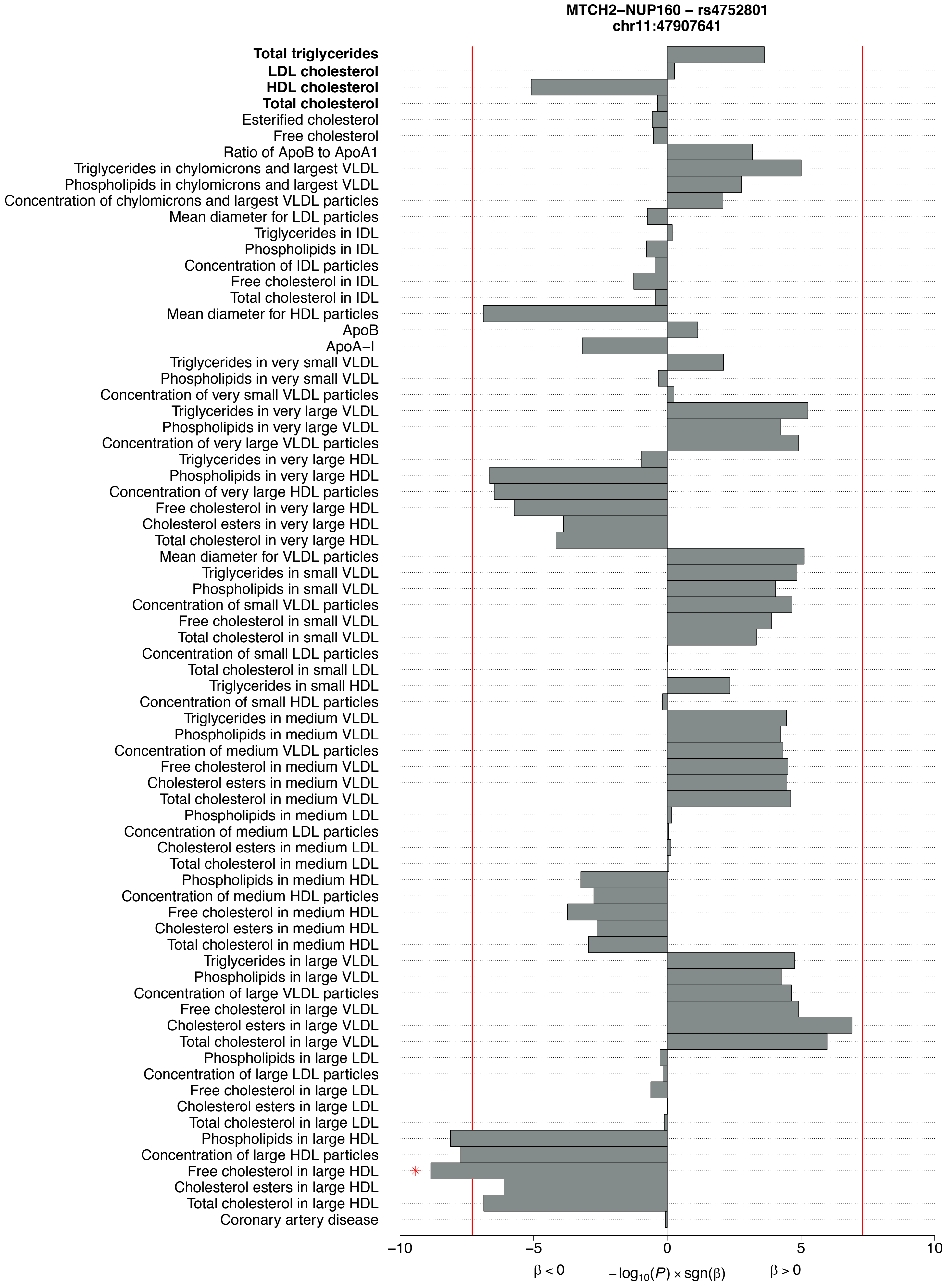
MTCH2–NUP160 – rs4752801
chr11:47907641
Total triglycerides
LDL cholesterol
HDL cholesterol
Total cholesterol
Esterified cholesterol
Free cholesterol
Ratio of ApoB to ApoA1
Triglycerides in chylomicrons and largest VLDL
Phospholipids in chylomicrons and largest VLDL
Concentration of chylomicrons and largest VLDL particles
Mean diameter for LDL particles
Triglycerides in IDL
Phospholipids in IDL
Concentration of IDL particles
Free cholesterol in IDL
Total cholesterol in IDL
Mean diameter for HDL particles
ApoB
ApoA–I
Triglycerides in very small VLDL
Phospholipids in very small VLDL
Concentration of very small VLDL particles
Triglycerides in very large VLDL
Phospholipids in very large VLDL
Concentration of very large VLDL particles
Triglycerides in very large HDL
Phospholipids in very large HDL
Concentration of very large HDL particles
Free cholesterol in very large HDL
Cholesterol esters in very large HDL
Total cholesterol in very large HDL
Mean diameter for VLDL particles
Triglycerides in small VLDL
Phospholipids in small VLDL
Concentration of small VLDL particles
Free cholesterol in small VLDL
Total cholesterol in small VLDL
Concentration of small LDL particles
Total cholesterol in small LDL
Triglycerides in small HDL
Concentration of small HDL particles
Triglycerides in medium VLDL
Phospholipids in medium VLDL
Concentration of medium VLDL particles
Free cholesterol in medium VLDL
Cholesterol esters in medium VLDL
Total cholesterol in medium VLDL
Phospholipids in medium LDL
Concentration of medium LDL particles
Cholesterol esters in medium LDL
Total cholesterol in medium LDL
Phospholipids in medium HDL
Concentration of medium HDL particles
Free cholesterol in medium HDL
Cholesterol esters in medium HDL
Total cholesterol in medium HDL
Triglycerides in large VLDL
Phospholipids in large VLDL
Concentration of large VLDL particles
Free cholesterol in large VLDL
Cholesterol esters in large VLDL
Total cholesterol in large VLDL
Phospholipids in large LDL
Concentration of large LDL particles
Free cholesterol in large LDL
Cholesterol esters in large LDL
Total cholesterol in large LDL
Phospholipids in large HDL
Concentration of large HDL particles
Free cholesterol in large HDL
Cholesterol esters in large HDL
Total cholesterol in large HDL
Coronary artery disease
−10
−5
0
5
10
β < 0
−log10(P) × sgn(β)
β > 0

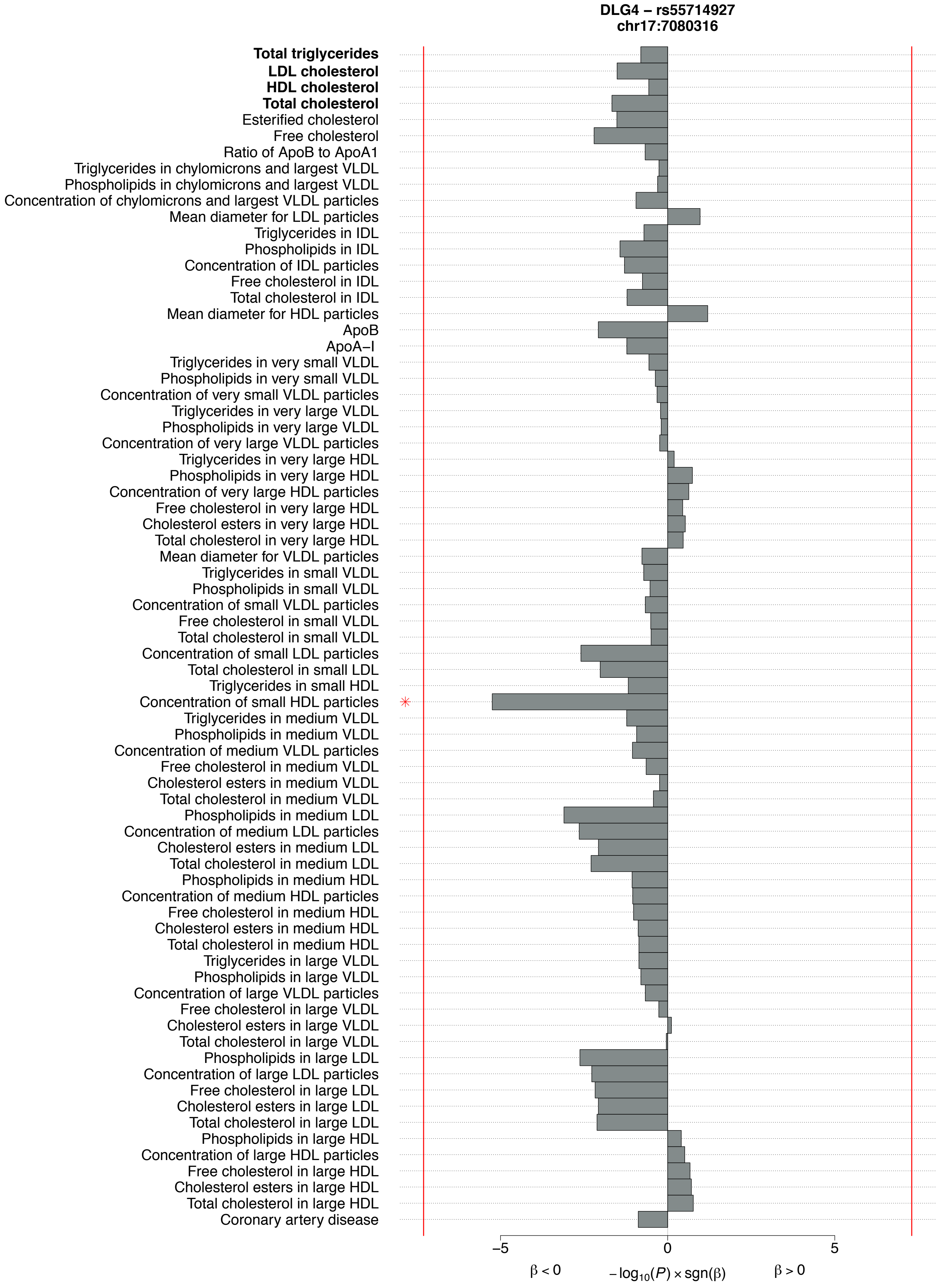

DLG4 – rs55714927
chr17:7080316
Total triglycerides
LDL cholesterol
HDL cholesterol
Total cholesterol
Esterified cholesterol
Free cholesterol
Ratio of ApoB to ApoA1
Triglycerides in chylomicrons and largest VLDL
Phospholipids in chylomicrons and largest VLDL
Concentration of chylomicrons and largest VLDL particles
Mean diameter for LDL particles
Triglycerides in IDL
Phospholipids in IDL
Concentration of IDL particles
Free cholesterol in IDL
Total cholesterol in IDL
Mean diameter for HDL particles
ApoB
ApoA–I
Triglycerides in very small VLDL
Phospholipids in very small VLDL
Concentration of very small VLDL particles
Triglycerides in very large VLDL
Phospholipids in very large VLDL
Concentration of very large VLDL particles
Triglycerides in very large HDL
Phospholipids in very large HDL
Concentration of very large HDL particles
Free cholesterol in very large HDL
Cholesterol esters in very large HDL
Total cholesterol in very large HDL
Mean diameter for VLDL particles
Triglycerides in small VLDL
Phospholipids in small VLDL
Concentration of small VLDL particles
Free cholesterol in small VLDL
Total cholesterol in small VLDL
Concentration of small LDL particles
Total cholesterol in small LDL
Triglycerides in small HDL
Concentration of small HDL particles
Triglycerides in medium VLDL
Phospholipids in medium VLDL
Concentration of medium VLDL particles
Free cholesterol in medium VLDL
Cholesterol esters in medium VLDL
Total cholesterol in medium VLDL
Phospholipids in medium LDL
Concentration of medium LDL particles
Cholesterol esters in medium LDL
Total cholesterol in medium LDL
Phospholipids in medium HDL
Concentration of medium HDL particles
Free cholesterol in medium HDL
Cholesterol esters in medium HDL
Total cholesterol in medium HDL
Triglycerides in large VLDL
Phospholipids in large VLDL
Concentration of large VLDL particles
Free cholesterol in large VLDL
Cholesterol esters in large VLDL
Total cholesterol in large VLDL
Phospholipids in large LDL
Concentration of large LDL particles
Free cholesterol in large LDL
Cholesterol esters in large LDL
Total cholesterol in large LDL
Phospholipids in large HDL
Concentration of large HDL particles
Free cholesterol in large HDL
Cholesterol esters in large HDL
Total cholesterol in large HDL
Coronary artery disease
−5
0
5
β < 0
−log10(P) × sgn(β)
β > 0

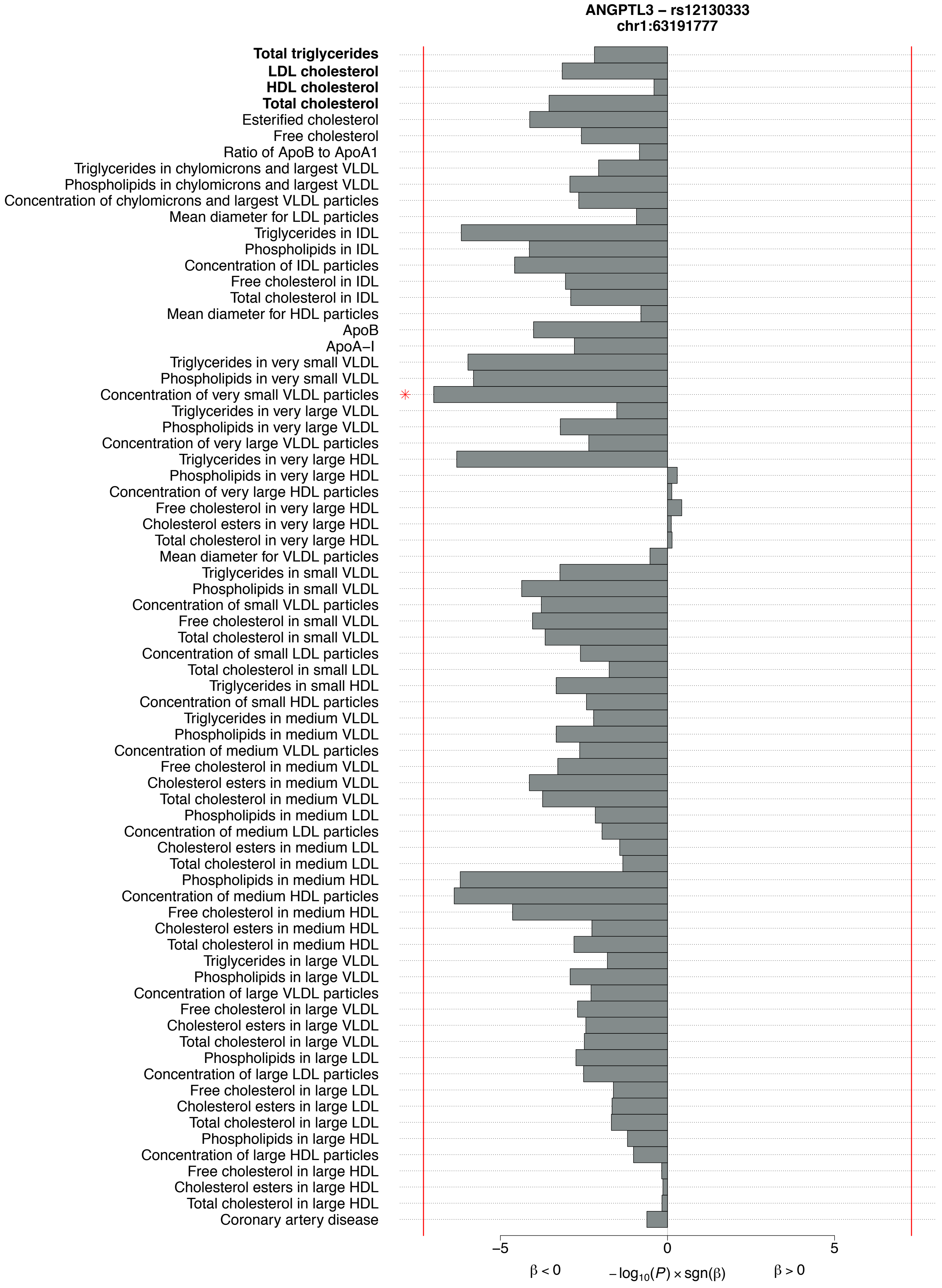
ANGPTL3 – rs12130333
chr1:63191777
Total triglycerides
LDL cholesterol
HDL cholesterol
Total cholesterol
Esterified cholesterol
Free cholesterol
Ratio of ApoB to ApoA1
Triglycerides in chylomicrons and largest VLDL
Phospholipids in chylomicrons and largest VLDL
Concentration of chylomicrons and largest VLDL particles
Mean diameter for LDL particles
Triglycerides in IDL
Phospholipids in IDL
Concentration of IDL particles
Free cholesterol in IDL
Total cholesterol in IDL
Mean diameter for HDL particles
ApoB
ApoA–I
Triglycerides in very small VLDL
Phospholipids in very small VLDL
Concentration of very small VLDL particles
Triglycerides in very large VLDL
Phospholipids in very large VLDL
Concentration of very large VLDL particles
Triglycerides in very large HDL
Phospholipids in very large HDL
Concentration of very large HDL particles
Free cholesterol in very large HDL
Cholesterol esters in very large HDL
Total cholesterol in very large HDL
Mean diameter for VLDL particles
Triglycerides in small VLDL
Phospholipids in small VLDL
Concentration of small VLDL particles
Free cholesterol in small VLDL
Total cholesterol in small VLDL
Concentration of small LDL particles
Total cholesterol in small LDL
Triglycerides in small HDL
Concentration of small HDL particles
Triglycerides in medium VLDL
Phospholipids in medium VLDL
Concentration of medium VLDL particles
Free cholesterol in medium VLDL
Cholesterol esters in medium VLDL
Total cholesterol in medium VLDL
Phospholipids in medium LDL
Concentration of medium LDL particles
Cholesterol esters in medium LDL
Total cholesterol in medium LDL
Phospholipids in medium HDL
Concentration of medium HDL particles
Free cholesterol in medium HDL
Cholesterol esters in medium HDL
Total cholesterol in medium HDL
Triglycerides in large VLDL
Phospholipids in large VLDL
Concentration of large VLDL particles
Free cholesterol in large VLDL
Cholesterol esters in large VLDL
Total cholesterol in large VLDL
Phospholipids in large LDL
Concentration of large LDL particles
Free cholesterol in large LDL
Cholesterol esters in large LDL
Total cholesterol in large LDL
Phospholipids in large HDL
Concentration of large HDL particles
Free cholesterol in large HDL
Cholesterol esters in large HDL
Total cholesterol in large HDL
Coronary artery disease
–5
0
5
β < 0
$-\log_{10}(P) \times \mathrm{sgn}(\beta)$
β > 0

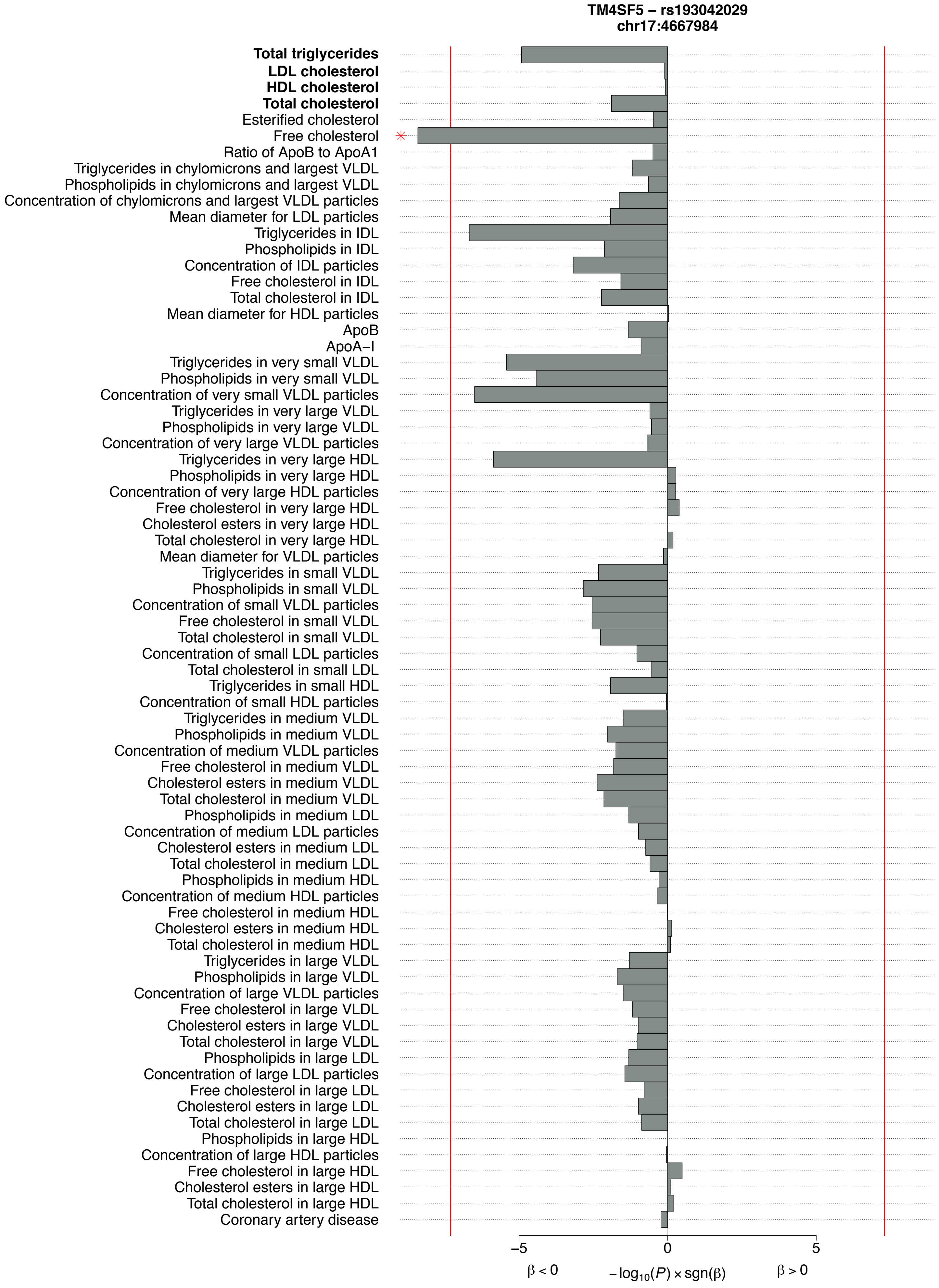

TM4SF5 – rs193042029
chr17:4667984
Total triglycerides
LDL cholesterol
HDL cholesterol
Total cholesterol
Esterified cholesterol
Free cholesterol
Ratio of ApoB to ApoA1
Triglycerides in chylomicrons and largest VLDL
Phospholipids in chylomicrons and largest VLDL
Concentration of chylomicrons and largest VLDL particles
Mean diameter for LDL particles
Triglycerides in IDL
Phospholipids in IDL
Concentration of IDL particles
Free cholesterol in IDL
Total cholesterol in IDL
Mean diameter for HDL particles
ApoB
ApoA–I
Triglycerides in very small VLDL
Phospholipids in very small VLDL
Concentration of very small VLDL particles
Triglycerides in very large VLDL
Phospholipids in very large VLDL
Concentration of very large VLDL particles
Triglycerides in very large HDL
Phospholipids in very large HDL
Concentration of very large HDL particles
Free cholesterol in very large HDL
Cholesterol esters in very large HDL
Total cholesterol in very large HDL
Mean diameter for VLDL particles
Triglycerides in small VLDL
Phospholipids in small VLDL
Concentration of small VLDL particles
Free cholesterol in small VLDL
Total cholesterol in small VLDL
Concentration of small LDL particles
Total cholesterol in small LDL
Triglycerides in small HDL
Concentration of small HDL particles
Triglycerides in medium VLDL
Phospholipids in medium VLDL
Concentration of medium VLDL particles
Free cholesterol in medium VLDL
Cholesterol esters in medium VLDL
Total cholesterol in medium VLDL
Phospholipids in medium LDL
Concentration of medium LDL particles
Cholesterol esters in medium LDL
Total cholesterol in medium LDL
Phospholipids in medium HDL
Concentration of medium HDL particles
Free cholesterol in medium HDL
Cholesterol esters in medium HDL
Total cholesterol in medium HDL
Triglycerides in large VLDL
Phospholipids in large VLDL
Concentration of large VLDL particles
Free cholesterol in large VLDL
Cholesterol esters in large VLDL
Total cholesterol in large VLDL
Phospholipids in large LDL
Concentration of large LDL particles
Free cholesterol in large LDL
Cholesterol esters in large LDL
Total cholesterol in large LDL
Phospholipids in large HDL
Concentration of large HDL particles
Free cholesterol in large HDL
Cholesterol esters in large HDL
Total cholesterol in large HDL
Coronary artery disease
–5
0
5
β < 0
– log10(P) × sgn(β)
β > 0

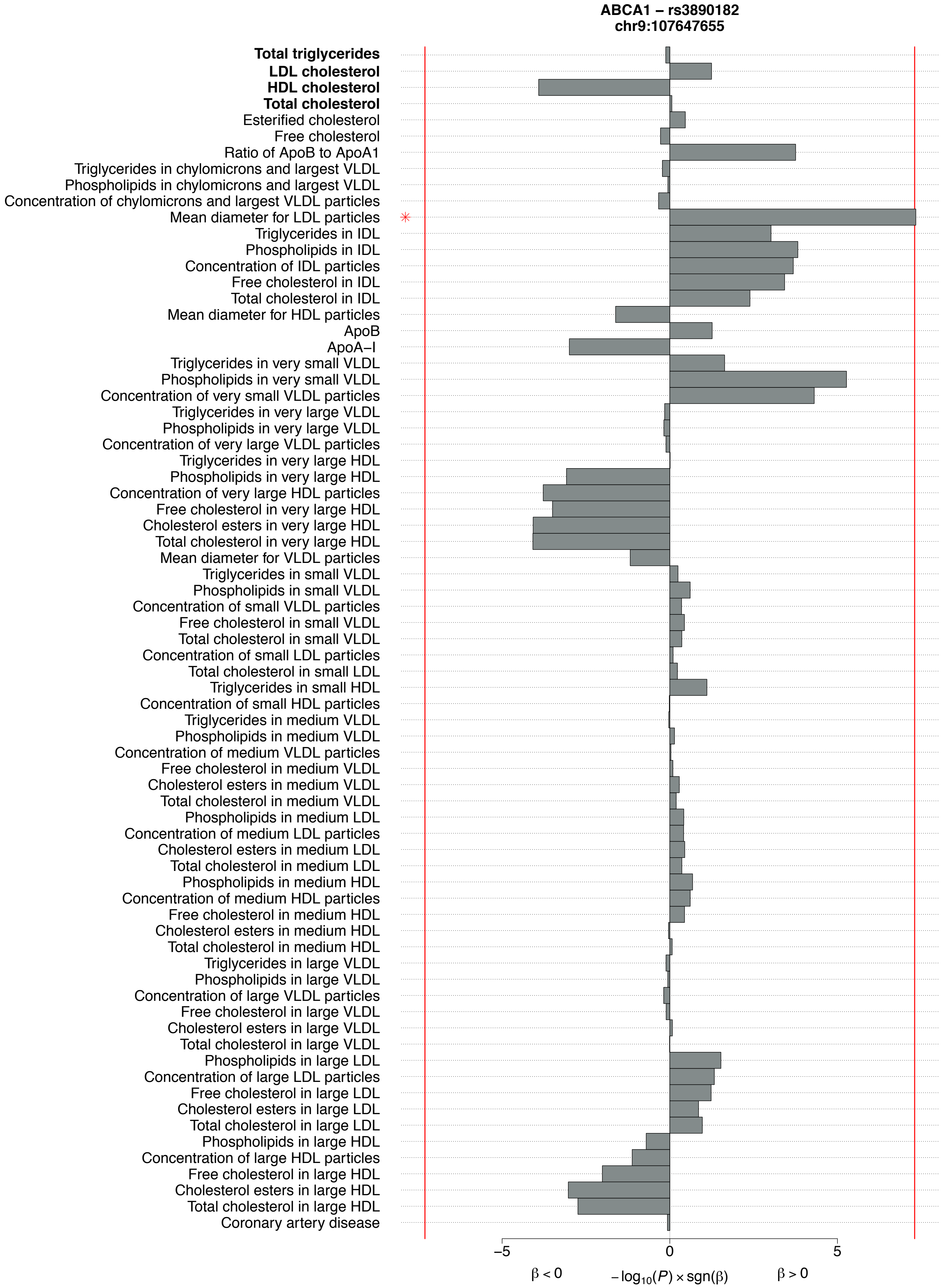
ABCA1 – rs3890182
chr9:107647655
Total triglycerides
LDL cholesterol
HDL cholesterol
Total cholesterol
Esterified cholesterol
Free cholesterol
Ratio of ApoB to ApoA1
Triglycerides in chylomicrons and largest VLDL
Phospholipids in chylomicrons and largest VLDL
Concentration of chylomicrons and largest VLDL particles
Mean diameter for LDL particles
Triglycerides in IDL
Phospholipids in IDL
Concentration of IDL particles
Free cholesterol in IDL
Total cholesterol in IDL
Mean diameter for HDL particles
ApoB
ApoA–I
Triglycerides in very small VLDL
Phospholipids in very small VLDL
Concentration of very small VLDL particles
Triglycerides in very large VLDL
Phospholipids in very large VLDL
Concentration of very large VLDL particles
Triglycerides in very large HDL
Phospholipids in very large HDL
Concentration of very large HDL particles
Free cholesterol in very large HDL
Cholesterol esters in very large HDL
Total cholesterol in very large HDL
Mean diameter for VLDL particles
Triglycerides in small VLDL
Phospholipids in small VLDL
Concentration of small VLDL particles
Free cholesterol in small VLDL
Total cholesterol in small VLDL
Concentration of small LDL particles
Total cholesterol in small LDL
Triglycerides in small HDL
Concentration of small HDL particles
Triglycerides in medium VLDL
Phospholipids in medium VLDL
Concentration of medium VLDL particles
Free cholesterol in medium VLDL
Cholesterol esters in medium VLDL
Total cholesterol in medium VLDL
Phospholipids in medium LDL
Concentration of medium LDL particles
Cholesterol esters in medium LDL
Total cholesterol in medium LDL
Phospholipids in medium HDL
Concentration of medium HDL particles
Free cholesterol in medium HDL
Cholesterol esters in medium HDL
Total cholesterol in medium HDL
Triglycerides in large VLDL
Phospholipids in large VLDL
Concentration of large VLDL particles
Free cholesterol in large VLDL
Cholesterol esters in large VLDL
Total cholesterol in large VLDL
Phospholipids in large LDL
Concentration of large LDL particles
Free cholesterol in large LDL
Cholesterol esters in large LDL
Total cholesterol in large LDL
Phospholipids in large HDL
Concentration of large HDL particles
Free cholesterol in large HDL
Cholesterol esters in large HDL
Total cholesterol in large HDL
Coronary artery disease
–5
0
5
β < 0
– log10(P) × sgn(β)
β > 0

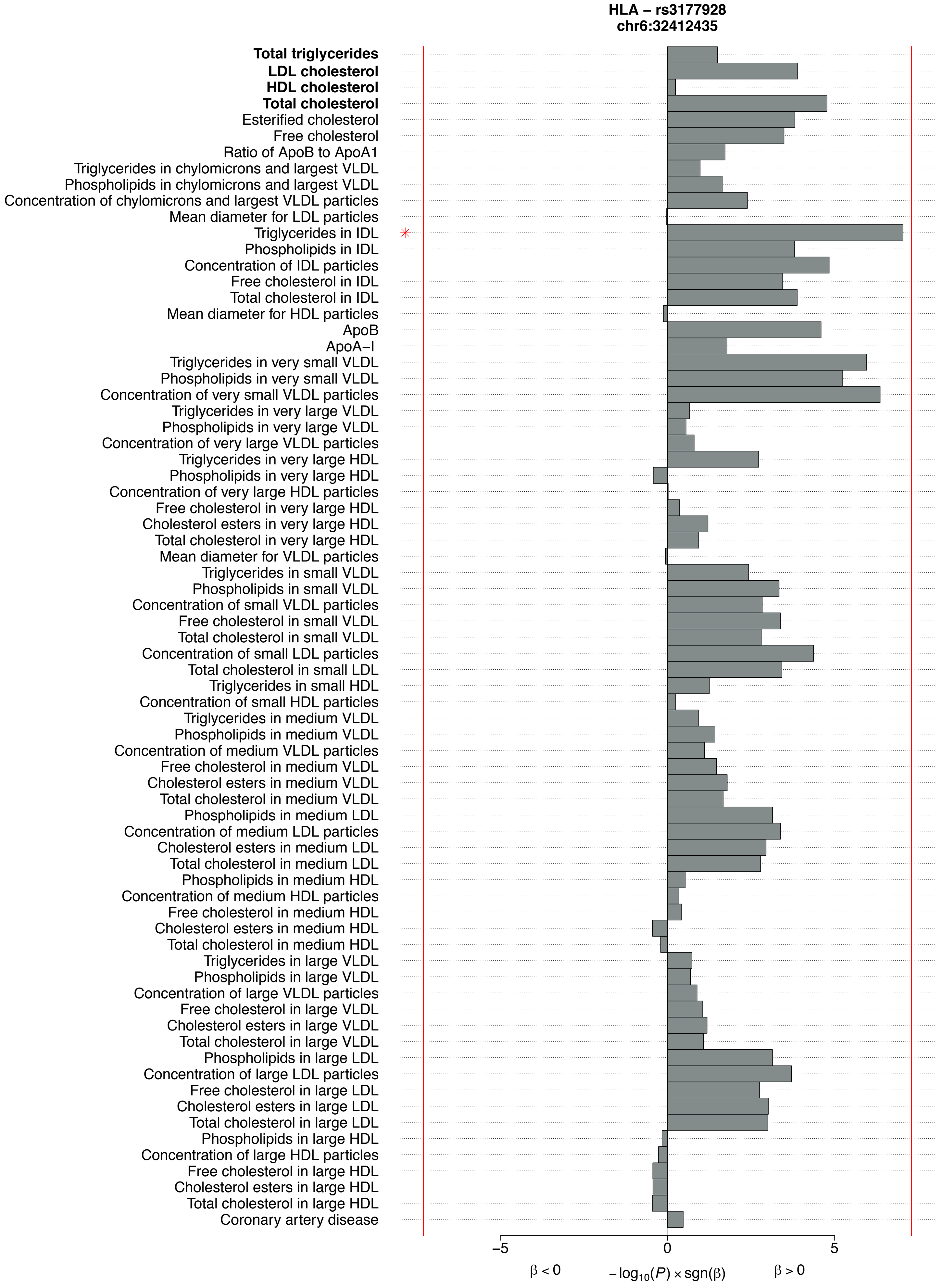
HLA – rs3177928
chr6:32412435
Total triglycerides
LDL cholesterol
HDL cholesterol
Total cholesterol
Esterified cholesterol
Free cholesterol
Ratio of ApoB to ApoA1
Triglycerides in chylomicrons and largest VLDL
Phospholipids in chylomicrons and largest VLDL
Concentration of chylomicrons and largest VLDL particles
Mean diameter for LDL particles
Triglycerides in IDL
Phospholipids in IDL
Concentration of IDL particles
Free cholesterol in IDL
Total cholesterol in IDL
Mean diameter for HDL particles
ApoB
ApoA–I
Triglycerides in very small VLDL
Phospholipids in very small VLDL
Concentration of very small VLDL particles
Triglycerides in very large VLDL
Phospholipids in very large VLDL
Concentration of very large VLDL particles
Triglycerides in very large HDL
Phospholipids in very large HDL
Concentration of very large HDL particles
Free cholesterol in very large HDL
Cholesterol esters in very large HDL
Total cholesterol in very large HDL
Mean diameter for VLDL particles
Triglycerides in small VLDL
Phospholipids in small VLDL
Concentration of small VLDL particles
Free cholesterol in small VLDL
Total cholesterol in small VLDL
Concentration of small LDL particles
Total cholesterol in small LDL
Triglycerides in small HDL
Concentration of small HDL particles
Triglycerides in medium VLDL
Phospholipids in medium VLDL
Concentration of medium VLDL particles
Free cholesterol in medium VLDL
Cholesterol esters in medium VLDL
Total cholesterol in medium VLDL
Phospholipids in medium LDL
Concentration of medium LDL particles
Cholesterol esters in medium LDL
Total cholesterol in medium LDL
Phospholipids in medium HDL
Concentration of medium HDL particles
Free cholesterol in medium HDL
Cholesterol esters in medium HDL
Total cholesterol in medium HDL
Triglycerides in large VLDL
Phospholipids in large VLDL
Concentration of large VLDL particles
Free cholesterol in large VLDL
Cholesterol esters in large VLDL
Total cholesterol in large VLDL
Phospholipids in large LDL
Concentration of large LDL particles
Free cholesterol in large LDL
Cholesterol esters in large LDL
Total cholesterol in large LDL
Phospholipids in large HDL
Concentration of large HDL particles
Free cholesterol in large HDL
Cholesterol esters in large HDL
Total cholesterol in large HDL
Coronary artery disease
–5
0
5
β < 0
$-\log_{10}(P) \times \mathrm{sgn}(\beta)$
β > 0

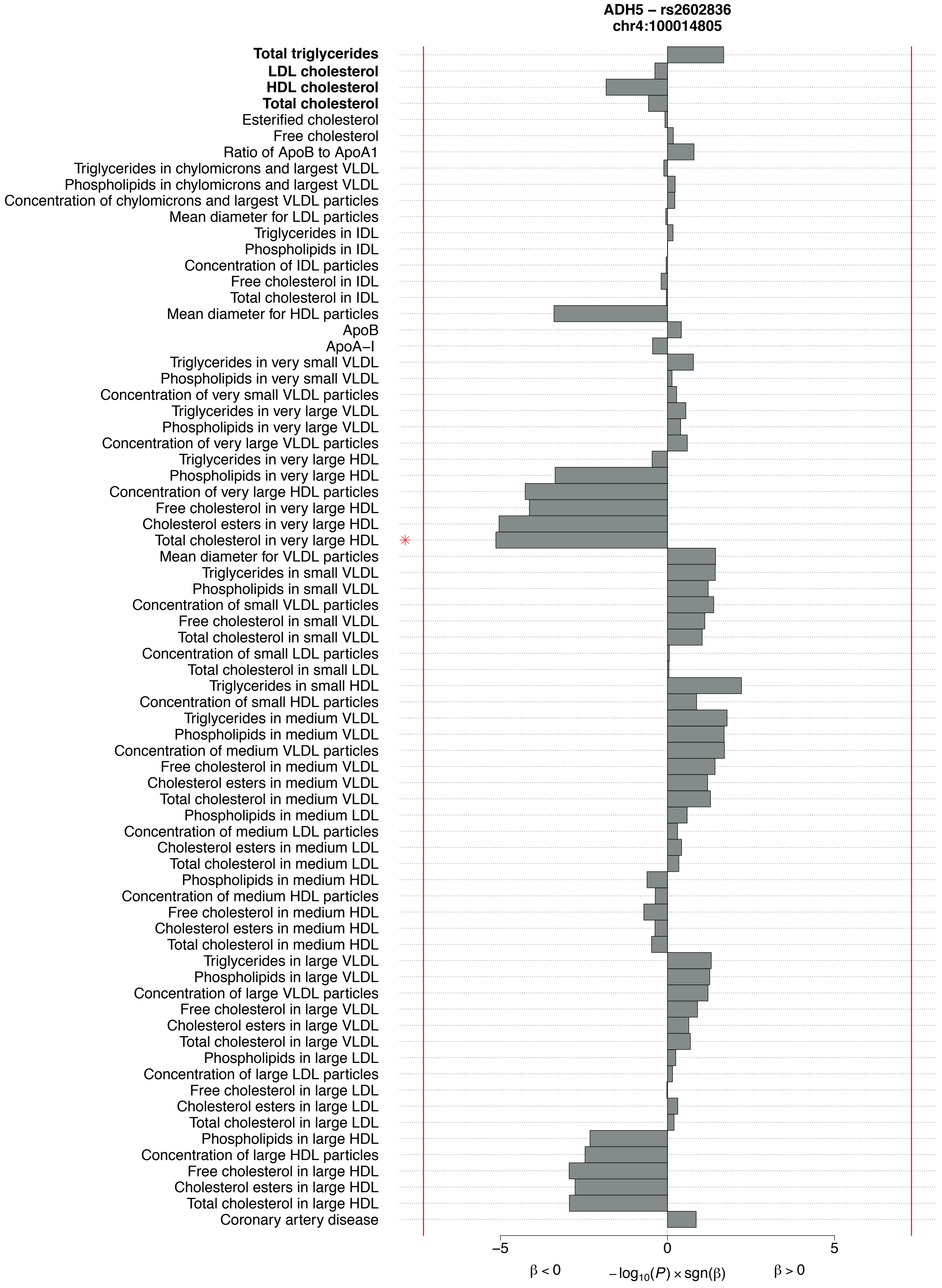
ADH5 – rs2602836
chr4:100014805
Total triglycerides
LDL cholesterol
HDL cholesterol
Total cholesterol
Esterified cholesterol
Free cholesterol
Ratio of ApoB to ApoA1
Triglycerides in chylomicrons and largest VLDL
Phospholipids in chylomicrons and largest VLDL
Concentration of chylomicrons and largest VLDL particles
Mean diameter for LDL particles
Triglycerides in IDL
Phospholipids in IDL
Concentration of IDL particles
Free cholesterol in IDL
Total cholesterol in IDL
Mean diameter for HDL particles
ApoB
ApoA−I
Triglycerides in very small VLDL
Phospholipids in very small VLDL
Concentration of very small VLDL particles
Triglycerides in very large VLDL
Phospholipids in very large VLDL
Concentration of very large VLDL particles
Triglycerides in very large HDL
Phospholipids in very large HDL
Concentration of very large HDL particles
Free cholesterol in very large HDL
Cholesterol esters in very large HDL
Total cholesterol in very large HDL
Mean diameter for VLDL particles
Triglycerides in small VLDL
Phospholipids in small VLDL
Concentration of small VLDL particles
Free cholesterol in small VLDL
Total cholesterol in small VLDL
Concentration of small LDL particles
Total cholesterol in small LDL
Triglycerides in small HDL
Concentration of small HDL particles
Triglycerides in medium VLDL
Phospholipids in medium VLDL
Concentration of medium VLDL particles
Free cholesterol in medium VLDL
Cholesterol esters in medium VLDL
Total cholesterol in medium VLDL
Phospholipids in medium LDL
Concentration of medium LDL particles
Cholesterol esters in medium LDL
Total cholesterol in medium LDL
Phospholipids in medium HDL
Concentration of medium HDL particles
Free cholesterol in medium HDL
Cholesterol esters in medium HDL
Total cholesterol in medium HDL
Triglycerides in large VLDL
Phospholipids in large VLDL
Concentration of large VLDL particles
Free cholesterol in large VLDL
Cholesterol esters in large VLDL
Total cholesterol in large VLDL
Phospholipids in large LDL
Concentration of large LDL particles
Free cholesterol in large LDL
Cholesterol esters in large LDL
Total cholesterol in large LDL
Phospholipids in large HDL
Concentration of large HDL particles
Free cholesterol in large HDL
Cholesterol esters in large HDL
Total cholesterol in large HDL
Coronary artery disease
−5
0
5
β < 0
$-\log_{10}(P) \times \mathrm{sgn}(\beta)$
β > 0

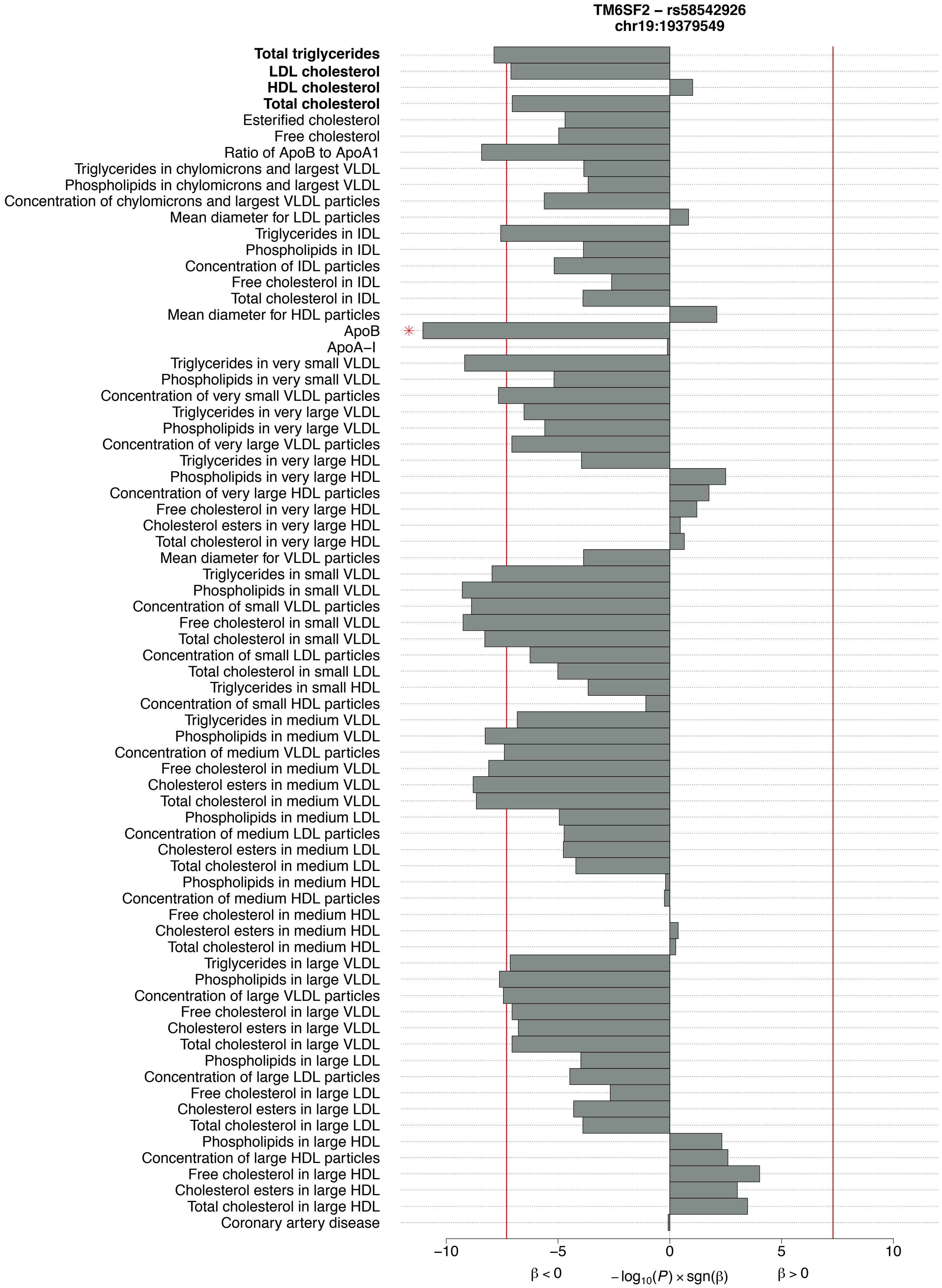
TM6SF2 – rs58542926
chr19:19379549
Total triglycerides
LDL cholesterol
HDL cholesterol
Total cholesterol
Esterified cholesterol
Free cholesterol
Ratio of ApoB to ApoA1
Triglycerides in chylomicrons and largest VLDL
Phospholipids in chylomicrons and largest VLDL
Concentration of chylomicrons and largest VLDL particles
Mean diameter for LDL particles
Triglycerides in IDL
Phospholipids in IDL
Concentration of IDL particles
Free cholesterol in IDL
Total cholesterol in IDL
Mean diameter for HDL particles
ApoB
ApoA–I
Triglycerides in very small VLDL
Phospholipids in very small VLDL
Concentration of very small VLDL particles
Triglycerides in very large VLDL
Phospholipids in very large VLDL
Concentration of very large VLDL particles
Triglycerides in very large HDL
Phospholipids in very large HDL
Concentration of very large HDL particles
Free cholesterol in very large HDL
Cholesterol esters in very large HDL
Total cholesterol in very large HDL
Mean diameter for VLDL particles
Triglycerides in small VLDL
Phospholipids in small VLDL
Concentration of small VLDL particles
Free cholesterol in small VLDL
Total cholesterol in small VLDL
Concentration of small LDL particles
Total cholesterol in small LDL
Triglycerides in small HDL
Concentration of small HDL particles
Triglycerides in medium VLDL
Phospholipids in medium VLDL
Concentration of medium VLDL particles
Free cholesterol in medium VLDL
Cholesterol esters in medium VLDL
Total cholesterol in medium VLDL
Phospholipids in medium LDL
Concentration of medium LDL particles
Cholesterol esters in medium LDL
Total cholesterol in medium LDL
Phospholipids in medium HDL
Concentration of medium HDL particles
Free cholesterol in medium HDL
Cholesterol esters in medium HDL
Total cholesterol in medium HDL
Triglycerides in large VLDL
Phospholipids in large VLDL
Concentration of large VLDL particles
Free cholesterol in large VLDL
Cholesterol esters in large VLDL
Total cholesterol in large VLDL
Phospholipids in large LDL
Concentration of large LDL particles
Free cholesterol in large LDL
Cholesterol esters in large LDL
Total cholesterol in large LDL
Phospholipids in large HDL
Concentration of large HDL particles
Free cholesterol in large HDL
Cholesterol esters in large HDL
Total cholesterol in large HDL
Coronary artery disease
−10
−5
0
5
10
β < 0
$-\log_{10}(P) \times \mathrm{sgn}(\beta)$
β > 0

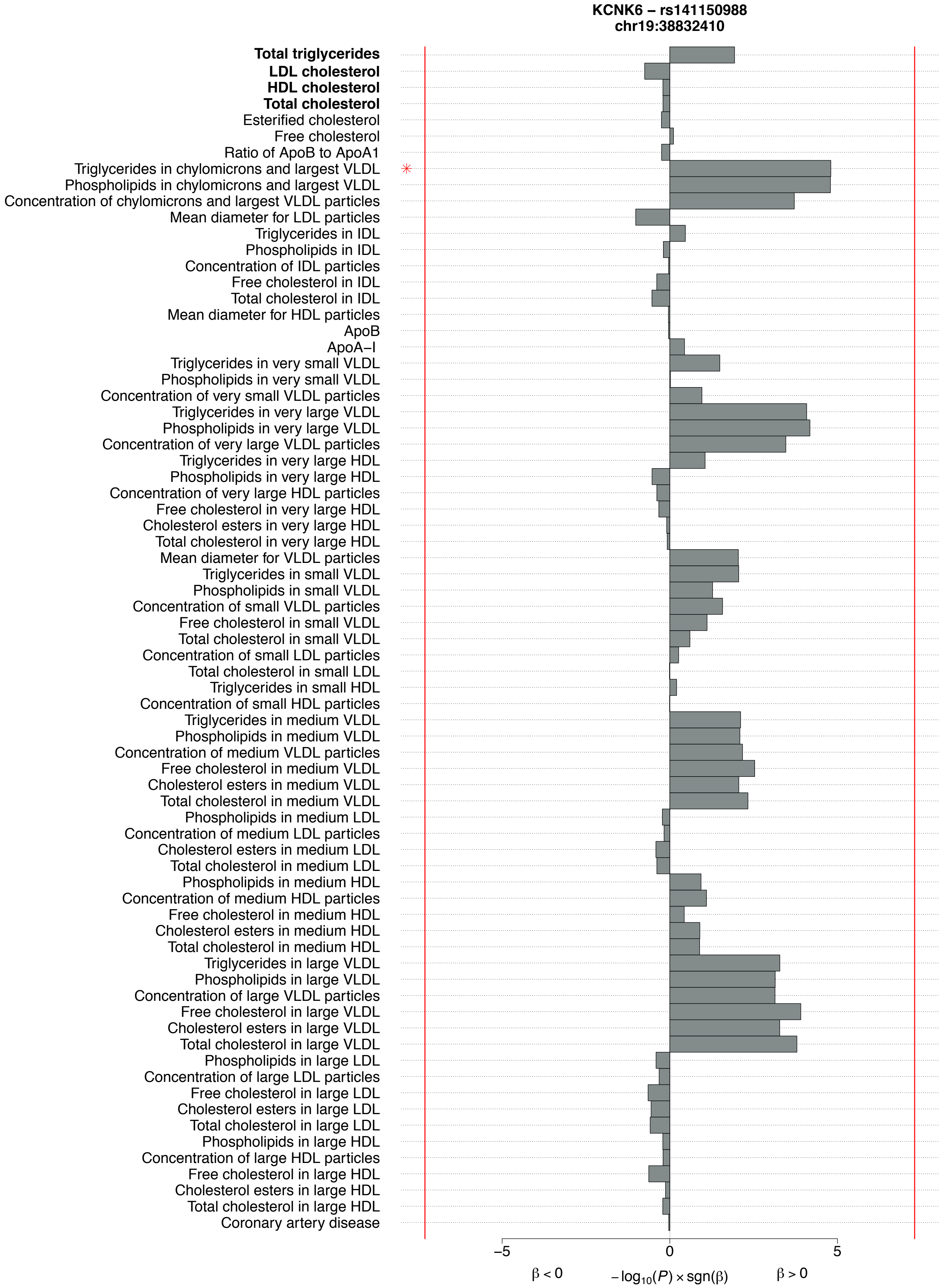
KCNK6 – rs141150988
chr19:38832410
Total triglycerides
LDL cholesterol
HDL cholesterol
Total cholesterol
Esterified cholesterol
Free cholesterol
Ratio of ApoB to ApoA1
Triglycerides in chylomicrons and largest VLDL
Phospholipids in chylomicrons and largest VLDL
Concentration of chylomicrons and largest VLDL particles
Mean diameter for LDL particles
Triglycerides in IDL
Phospholipids in IDL
Concentration of IDL particles
Free cholesterol in IDL
Total cholesterol in IDL
Mean diameter for HDL particles
ApoB
ApoA–I
Triglycerides in very small VLDL
Phospholipids in very small VLDL
Concentration of very small VLDL particles
Triglycerides in very large VLDL
Phospholipids in very large VLDL
Concentration of very large VLDL particles
Triglycerides in very large HDL
Phospholipids in very large HDL
Concentration of very large HDL particles
Free cholesterol in very large HDL
Cholesterol esters in very large HDL
Total cholesterol in very large HDL
Mean diameter for VLDL particles
Triglycerides in small VLDL
Phospholipids in small VLDL
Concentration of small VLDL particles
Free cholesterol in small VLDL
Total cholesterol in small VLDL
Concentration of small LDL particles
Total cholesterol in small LDL
Triglycerides in small HDL
Concentration of small HDL particles
Triglycerides in medium VLDL
Phospholipids in medium VLDL
Concentration of medium VLDL particles
Free cholesterol in medium VLDL
Cholesterol esters in medium VLDL
Total cholesterol in medium VLDL
Phospholipids in medium LDL
Concentration of medium LDL particles
Cholesterol esters in medium LDL
Total cholesterol in medium LDL
Phospholipids in medium HDL
Concentration of medium HDL particles
Free cholesterol in medium HDL
Cholesterol esters in medium HDL
Total cholesterol in medium HDL
Triglycerides in large VLDL
Phospholipids in large VLDL
Concentration of large VLDL particles
Free cholesterol in large VLDL
Cholesterol esters in large VLDL
Total cholesterol in large VLDL
Phospholipids in large LDL
Concentration of large LDL particles
Free cholesterol in large LDL
Cholesterol esters in large LDL
Total cholesterol in large LDL
Phospholipids in large HDL
Concentration of large HDL particles
Free cholesterol in large HDL
Cholesterol esters in large HDL
Total cholesterol in large HDL
Coronary artery disease
–5
0
5
β < 0
$-\log_{10}(P) \times \mathrm{sgn}(\beta)$
β > 0

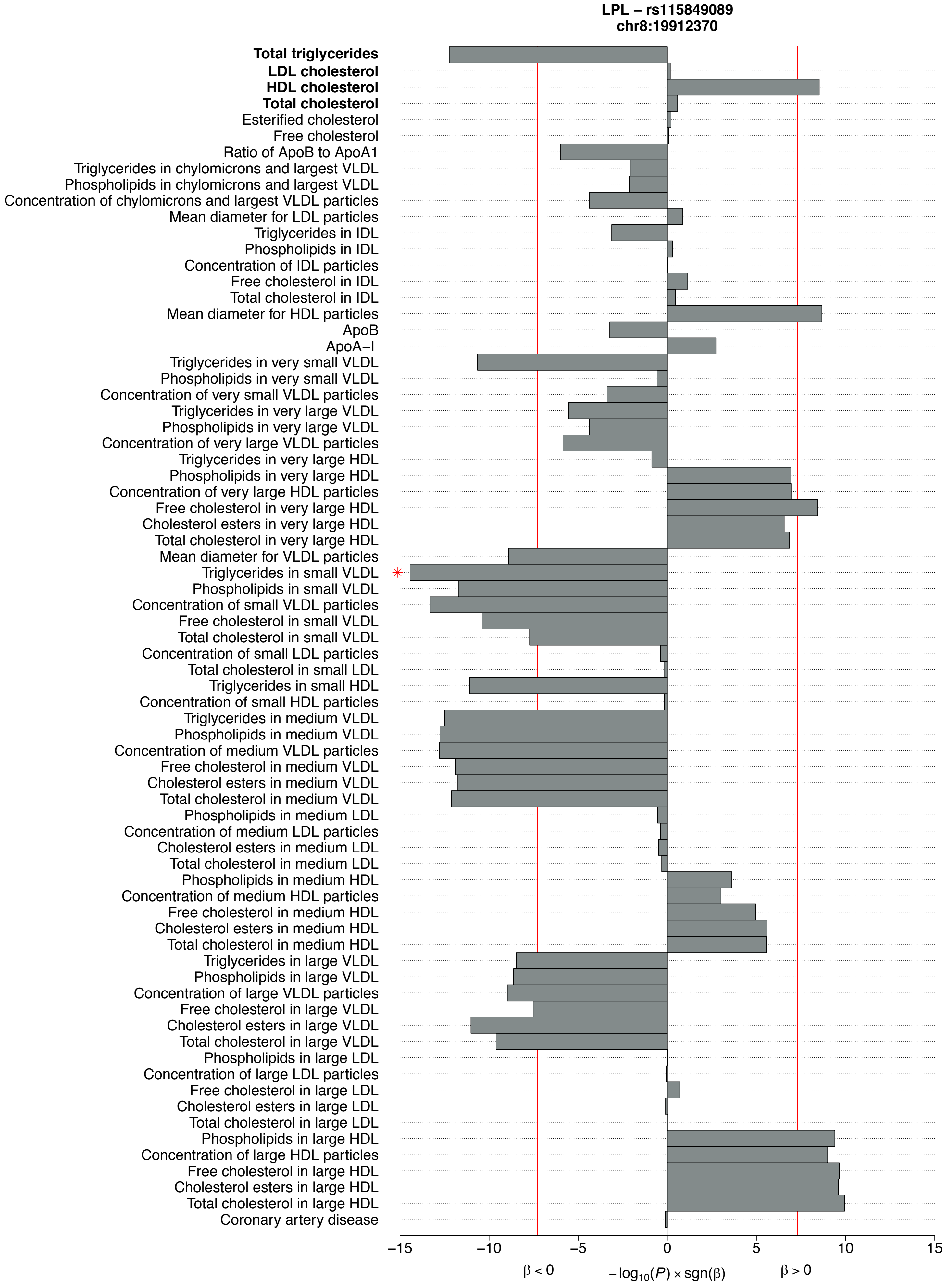
LPL – rs115849089
chr8:19912370
Total triglycerides
LDL cholesterol
HDL cholesterol
Total cholesterol
Esterified cholesterol
Free cholesterol
Ratio of ApoB to ApoA1
Triglycerides in chylomicrons and largest VLDL
Phospholipids in chylomicrons and largest VLDL
Concentration of chylomicrons and largest VLDL particles
Mean diameter for LDL particles
Triglycerides in IDL
Phospholipids in IDL
Concentration of IDL particles
Free cholesterol in IDL
Total cholesterol in IDL
Mean diameter for HDL particles
ApoB
ApoA–I
Triglycerides in very small VLDL
Phospholipids in very small VLDL
Concentration of very small VLDL particles
Triglycerides in very large VLDL
Phospholipids in very large VLDL
Concentration of very large VLDL particles
Triglycerides in very large HDL
Phospholipids in very large HDL
Concentration of very large HDL particles
Free cholesterol in very large HDL
Cholesterol esters in very large HDL
Total cholesterol in very large HDL
Mean diameter for VLDL particles
Triglycerides in small VLDL
Phospholipids in small VLDL
Concentration of small VLDL particles
Free cholesterol in small VLDL
Total cholesterol in small VLDL
Concentration of small LDL particles
Total cholesterol in small LDL
Triglycerides in small HDL
Concentration of small HDL particles
Triglycerides in medium VLDL
Phospholipids in medium VLDL
Concentration of medium VLDL particles
Free cholesterol in medium VLDL
Cholesterol esters in medium VLDL
Total cholesterol in medium VLDL
Phospholipids in medium LDL
Concentration of medium LDL particles
Cholesterol esters in medium LDL
Total cholesterol in medium LDL
Phospholipids in medium HDL
Concentration of medium HDL particles
Free cholesterol in medium HDL
Cholesterol esters in medium HDL
Total cholesterol in medium HDL
Triglycerides in large VLDL
Phospholipids in large VLDL
Concentration of large VLDL particles
Free cholesterol in large VLDL
Cholesterol esters in large VLDL
Total cholesterol in large VLDL
Phospholipids in large LDL
Concentration of large LDL particles
Free cholesterol in large LDL
Cholesterol esters in large LDL
Total cholesterol in large LDL
Phospholipids in large HDL
Concentration of large HDL particles
Free cholesterol in large HDL
Cholesterol esters in large HDL
Total cholesterol in large HDL
Coronary artery disease
–15
–10
–5
0
5
10
15
β < 0
– log10(P) × sgn(β)
β > 0

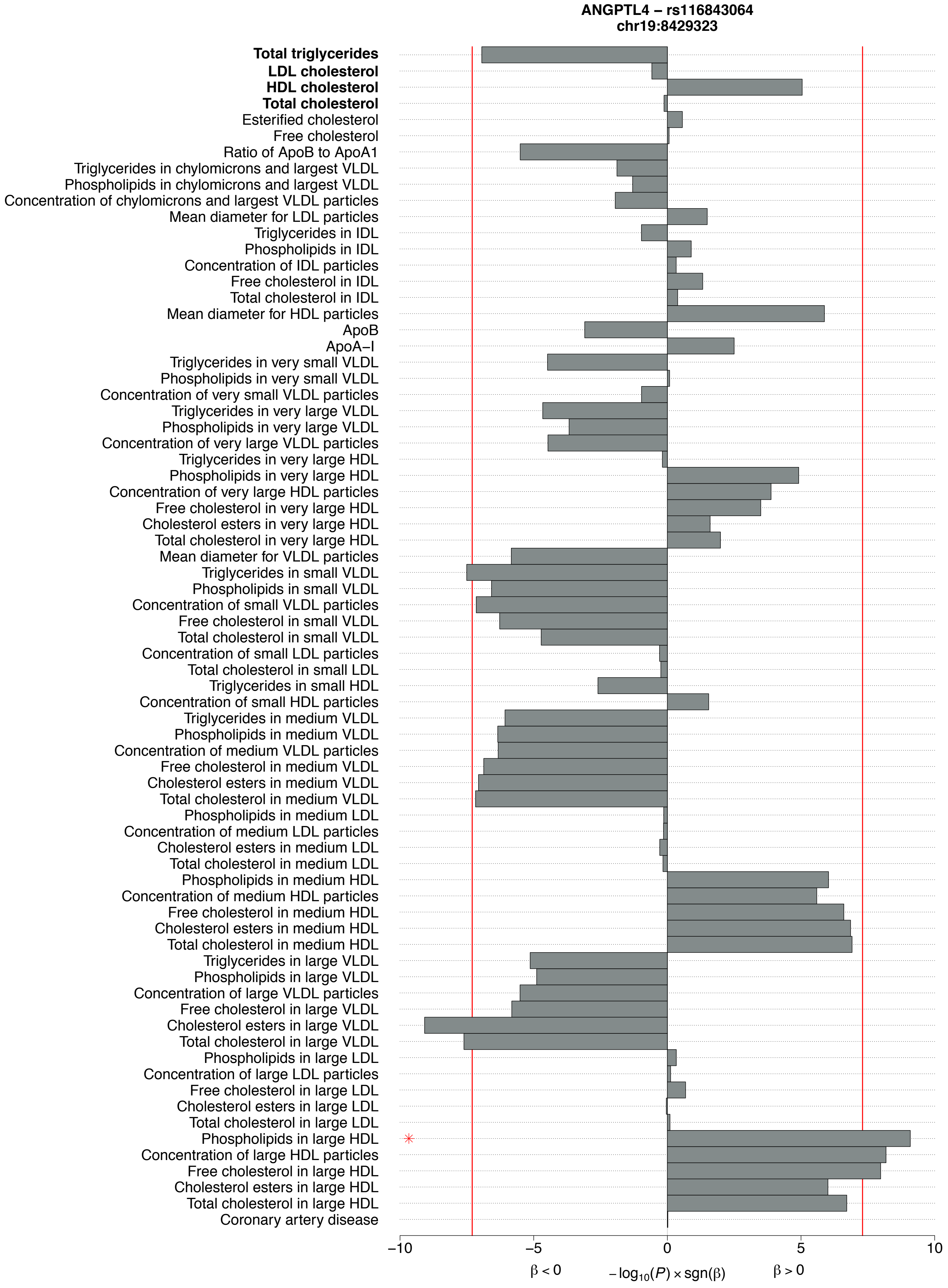
ANGPTL4 – rs116843064
chr19:8429323
Total triglycerides
LDL cholesterol
HDL cholesterol
Total cholesterol
Esterified cholesterol
Free cholesterol
Ratio of ApoB to ApoA1
Triglycerides in chylomicrons and largest VLDL
Phospholipids in chylomicrons and largest VLDL
Concentration of chylomicrons and largest VLDL particles
Mean diameter for LDL particles
Triglycerides in IDL
Phospholipids in IDL
Concentration of IDL particles
Free cholesterol in IDL
Total cholesterol in IDL
Mean diameter for HDL particles
ApoB
ApoA−I
Triglycerides in very small VLDL
Phospholipids in very small VLDL
Concentration of very small VLDL particles
Triglycerides in very large VLDL
Phospholipids in very large VLDL
Concentration of very large VLDL particles
Triglycerides in very large HDL
Phospholipids in very large HDL
Concentration of very large HDL particles
Free cholesterol in very large HDL
Cholesterol esters in very large HDL
Total cholesterol in very large HDL
Mean diameter for VLDL particles
Triglycerides in small VLDL
Phospholipids in small VLDL
Concentration of small VLDL particles
Free cholesterol in small VLDL
Total cholesterol in small VLDL
Concentration of small LDL particles
Total cholesterol in small LDL
Triglycerides in small HDL
Concentration of small HDL particles
Triglycerides in medium VLDL
Phospholipids in medium VLDL
Concentration of medium VLDL particles
Free cholesterol in medium VLDL
Cholesterol esters in medium VLDL
Total cholesterol in medium VLDL
Phospholipids in medium LDL
Concentration of medium LDL particles
Cholesterol esters in medium LDL
Total cholesterol in medium LDL
Phospholipids in medium HDL
Concentration of medium HDL particles
Free cholesterol in medium HDL
Cholesterol esters in medium HDL
Total cholesterol in medium HDL
Triglycerides in large VLDL
Phospholipids in large VLDL
Concentration of large VLDL particles
Free cholesterol in large VLDL
Cholesterol esters in large VLDL
Total cholesterol in large VLDL
Phospholipids in large LDL
Concentration of large LDL particles
Free cholesterol in large LDL
Cholesterol esters in large LDL
Total cholesterol in large LDL
Phospholipids in large HDL
Concentration of large HDL particles
Free cholesterol in large HDL
Cholesterol esters in large HDL
Total cholesterol in large HDL
Coronary artery disease
−10
−5
0
5
10
β < 0
−log10(P) × sgn(β)
β > 0

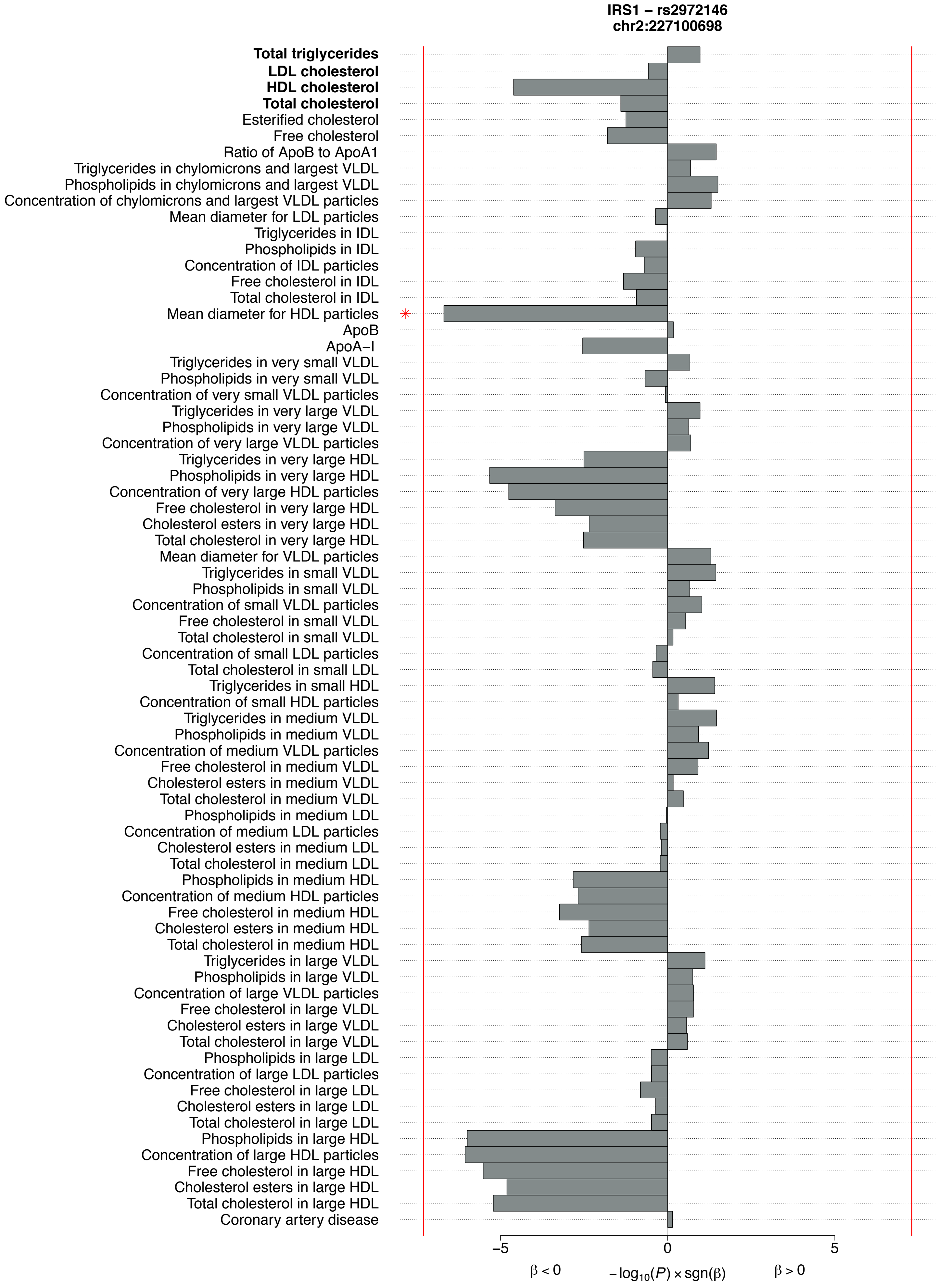
IRS1 – rs2972146
chr2:227100698
Total triglycerides
LDL cholesterol
HDL cholesterol
Total cholesterol
Esterified cholesterol
Free cholesterol
Ratio of ApoB to ApoA1
Triglycerides in chylomicrons and largest VLDL
Phospholipids in chylomicrons and largest VLDL
Concentration of chylomicrons and largest VLDL particles
Mean diameter for LDL particles
Triglycerides in IDL
Phospholipids in IDL
Concentration of IDL particles
Free cholesterol in IDL
Total cholesterol in IDL
Mean diameter for HDL particles
ApoB
ApoA−I
Triglycerides in very small VLDL
Phospholipids in very small VLDL
Concentration of very small VLDL particles
Triglycerides in very large VLDL
Phospholipids in very large VLDL
Concentration of very large VLDL particles
Triglycerides in very large HDL
Phospholipids in very large HDL
Concentration of very large HDL particles
Free cholesterol in very large HDL
Cholesterol esters in very large HDL
Total cholesterol in very large HDL
Mean diameter for VLDL particles
Triglycerides in small VLDL
Phospholipids in small VLDL
Concentration of small VLDL particles
Free cholesterol in small VLDL
Total cholesterol in small VLDL
Concentration of small LDL particles
Total cholesterol in small LDL
Triglycerides in small HDL
Concentration of small HDL particles
Triglycerides in medium VLDL
Phospholipids in medium VLDL
Concentration of medium VLDL particles
Free cholesterol in medium VLDL
Cholesterol esters in medium VLDL
Total cholesterol in medium VLDL
Phospholipids in medium LDL
Concentration of medium LDL particles
Cholesterol esters in medium LDL
Total cholesterol in medium LDL
Phospholipids in medium HDL
Concentration of medium HDL particles
Free cholesterol in medium HDL
Cholesterol esters in medium HDL
Total cholesterol in medium HDL
Triglycerides in large VLDL
Phospholipids in large VLDL
Concentration of large VLDL particles
Free cholesterol in large VLDL
Cholesterol esters in large VLDL
Total cholesterol in large VLDL
Phospholipids in large LDL
Concentration of large LDL particles
Free cholesterol in large LDL
Cholesterol esters in large LDL
Total cholesterol in large LDL
Phospholipids in large HDL
Concentration of large HDL particles
Free cholesterol in large HDL
Cholesterol esters in large HDL
Total cholesterol in large HDL
Coronary artery disease
−5
0
5
β < 0
− log10(P) × sgn(β)
β > 0

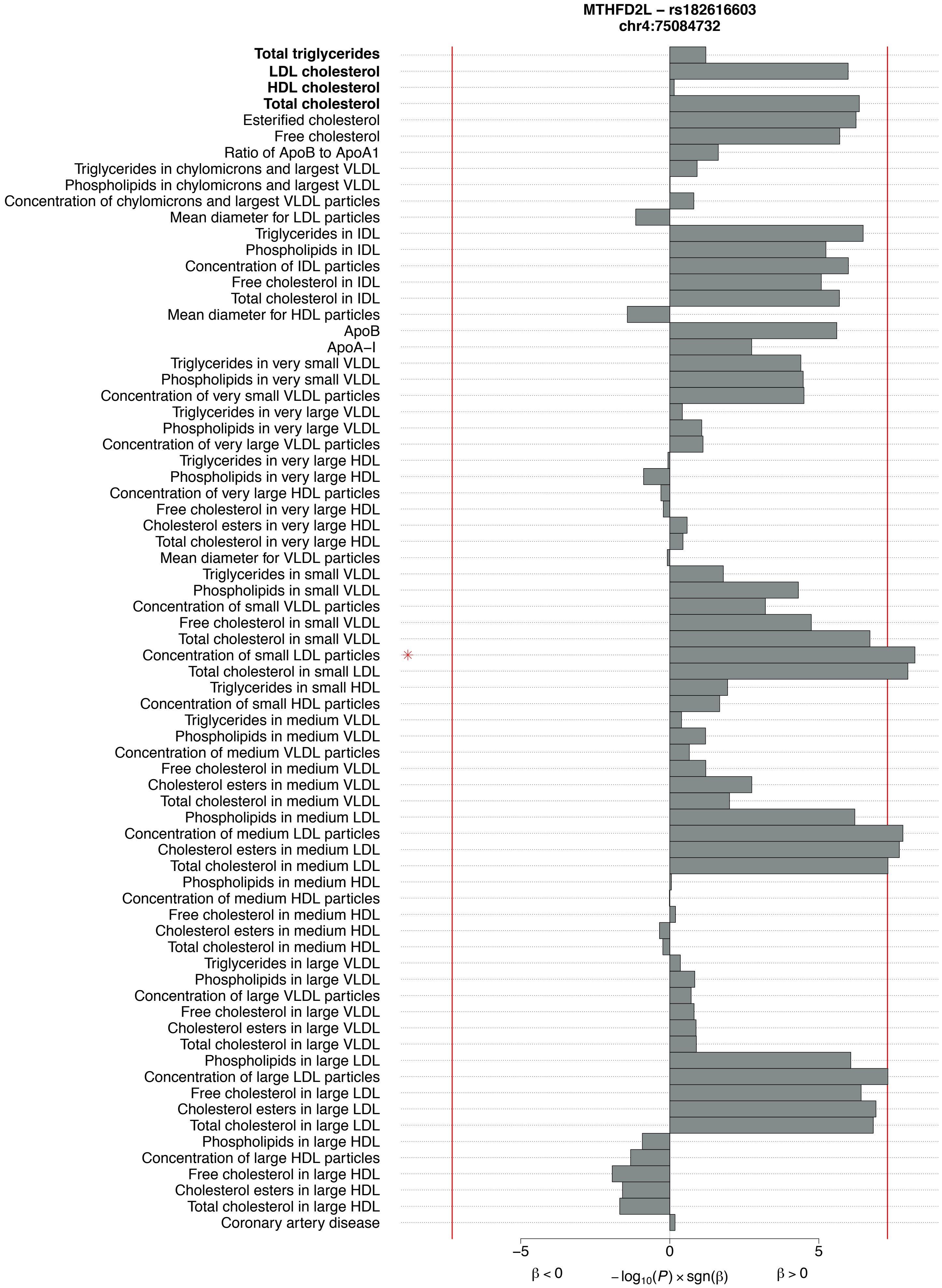
MTHFD2L – rs182616603
chr4:75084732
Total triglycerides
LDL cholesterol
HDL cholesterol
Total cholesterol
Esterified cholesterol
Free cholesterol
Ratio of ApoB to ApoA1
Triglycerides in chylomicrons and largest VLDL
Phospholipids in chylomicrons and largest VLDL
Concentration of chylomicrons and largest VLDL particles
Mean diameter for LDL particles
Triglycerides in IDL
Phospholipids in IDL
Concentration of IDL particles
Free cholesterol in IDL
Total cholesterol in IDL
Mean diameter for HDL particles
ApoB
ApoA–I
Triglycerides in very small VLDL
Phospholipids in very small VLDL
Concentration of very small VLDL particles
Triglycerides in very large VLDL
Phospholipids in very large VLDL
Concentration of very large VLDL particles
Triglycerides in very large HDL
Phospholipids in very large HDL
Concentration of very large HDL particles
Free cholesterol in very large HDL
Cholesterol esters in very large HDL
Total cholesterol in very large HDL
Mean diameter for VLDL particles
Triglycerides in small VLDL
Phospholipids in small VLDL
Concentration of small VLDL particles
Free cholesterol in small VLDL
Total cholesterol in small VLDL
Concentration of small LDL particles
Total cholesterol in small LDL
Triglycerides in small HDL
Concentration of small HDL particles
Triglycerides in medium VLDL
Phospholipids in medium VLDL
Concentration of medium VLDL particles
Free cholesterol in medium VLDL
Cholesterol esters in medium VLDL
Total cholesterol in medium VLDL
Phospholipids in medium LDL
Concentration of medium LDL particles
Cholesterol esters in medium LDL
Total cholesterol in medium LDL
Phospholipids in medium HDL
Concentration of medium HDL particles
Free cholesterol in medium HDL
Cholesterol esters in medium HDL
Total cholesterol in medium HDL
Triglycerides in large VLDL
Phospholipids in large VLDL
Concentration of large VLDL particles
Free cholesterol in large VLDL
Cholesterol esters in large VLDL
Total cholesterol in large VLDL
Phospholipids in large LDL
Concentration of large LDL particles
Free cholesterol in large LDL
Cholesterol esters in large LDL
Total cholesterol in large LDL
Phospholipids in large HDL
Concentration of large HDL particles
Free cholesterol in large HDL
Cholesterol esters in large HDL
Total cholesterol in large HDL
Coronary artery disease
–5
0
5
β < 0
$-\log_{10}(P) \times \mathrm{sgn}(\beta)$
β > 0

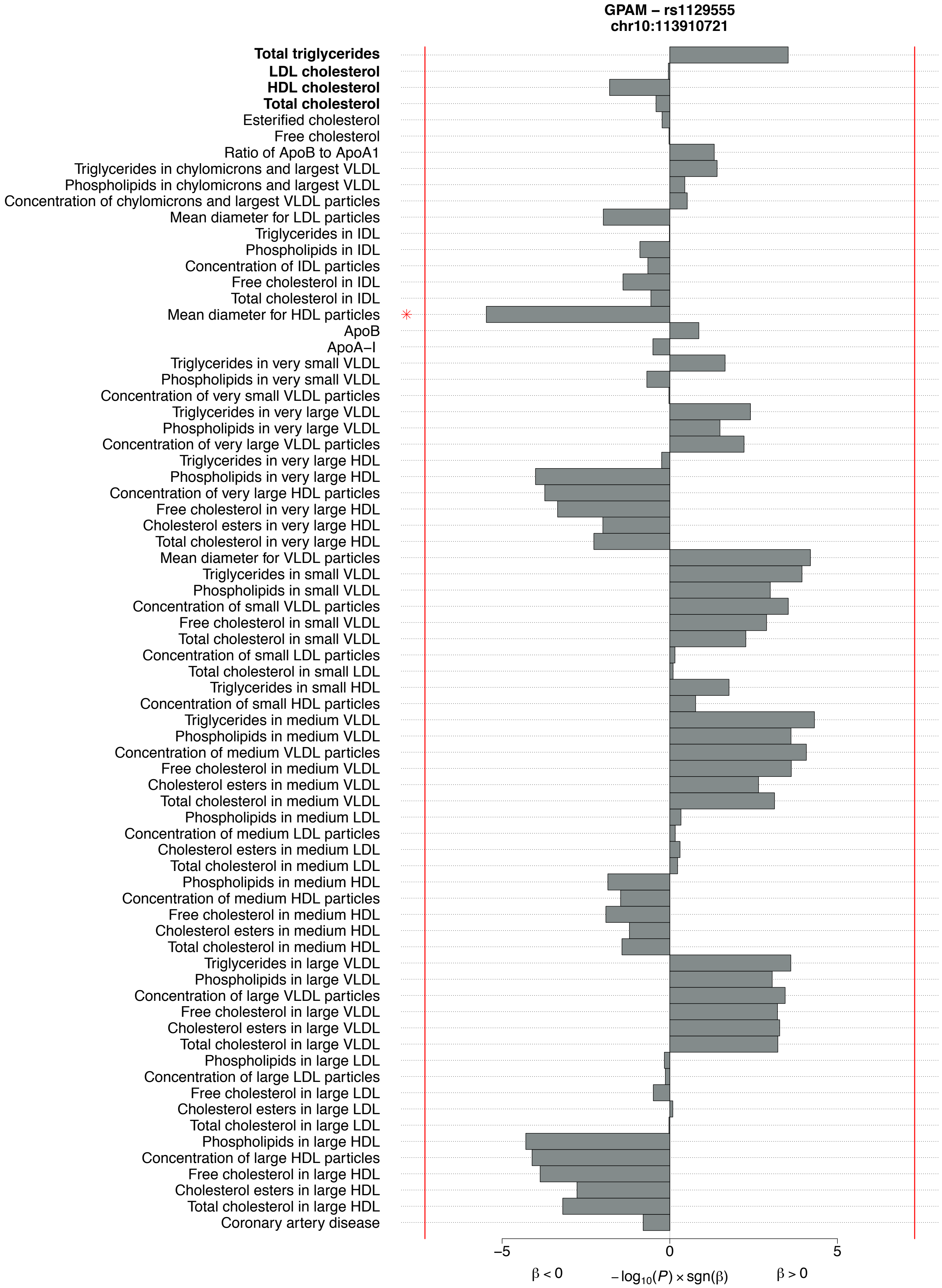
GPAM – rs1129555
chr10:113910721
Total triglycerides
LDL cholesterol
HDL cholesterol
Total cholesterol
Esterified cholesterol
Free cholesterol
Ratio of ApoB to ApoA1
Triglycerides in chylomicrons and largest VLDL
Phospholipids in chylomicrons and largest VLDL
Concentration of chylomicrons and largest VLDL particles
Mean diameter for LDL particles
Triglycerides in IDL
Phospholipids in IDL
Concentration of IDL particles
Free cholesterol in IDL
Total cholesterol in IDL
Mean diameter for HDL particles
ApoB
ApoA–I
Triglycerides in very small VLDL
Phospholipids in very small VLDL
Concentration of very small VLDL particles
Triglycerides in very large VLDL
Phospholipids in very large VLDL
Concentration of very large VLDL particles
Triglycerides in very large HDL
Phospholipids in very large HDL
Concentration of very large HDL particles
Free cholesterol in very large HDL
Cholesterol esters in very large HDL
Total cholesterol in very large HDL
Mean diameter for VLDL particles
Triglycerides in small VLDL
Phospholipids in small VLDL
Concentration of small VLDL particles
Free cholesterol in small VLDL
Total cholesterol in small VLDL
Concentration of small LDL particles
Total cholesterol in small LDL
Triglycerides in small HDL
Concentration of small HDL particles
Triglycerides in medium VLDL
Phospholipids in medium VLDL
Concentration of medium VLDL particles
Free cholesterol in medium VLDL
Cholesterol esters in medium VLDL
Total cholesterol in medium VLDL
Phospholipids in medium LDL
Concentration of medium LDL particles
Cholesterol esters in medium LDL
Total cholesterol in medium LDL
Phospholipids in medium HDL
Concentration of medium HDL particles
Free cholesterol in medium HDL
Cholesterol esters in medium HDL
Total cholesterol in medium HDL
Triglycerides in large VLDL
Phospholipids in large VLDL
Concentration of large VLDL particles
Free cholesterol in large VLDL
Cholesterol esters in large VLDL
Total cholesterol in large VLDL
Phospholipids in large LDL
Concentration of large LDL particles
Free cholesterol in large LDL
Cholesterol esters in large LDL
Total cholesterol in large LDL
Phospholipids in large HDL
Concentration of large HDL particles
Free cholesterol in large HDL
Cholesterol esters in large HDL
Total cholesterol in large HDL
Coronary artery disease
–5
0
5
β < 0
$-\log_{10}(P) \times \mathrm{sgn}(\beta)$
β > 0

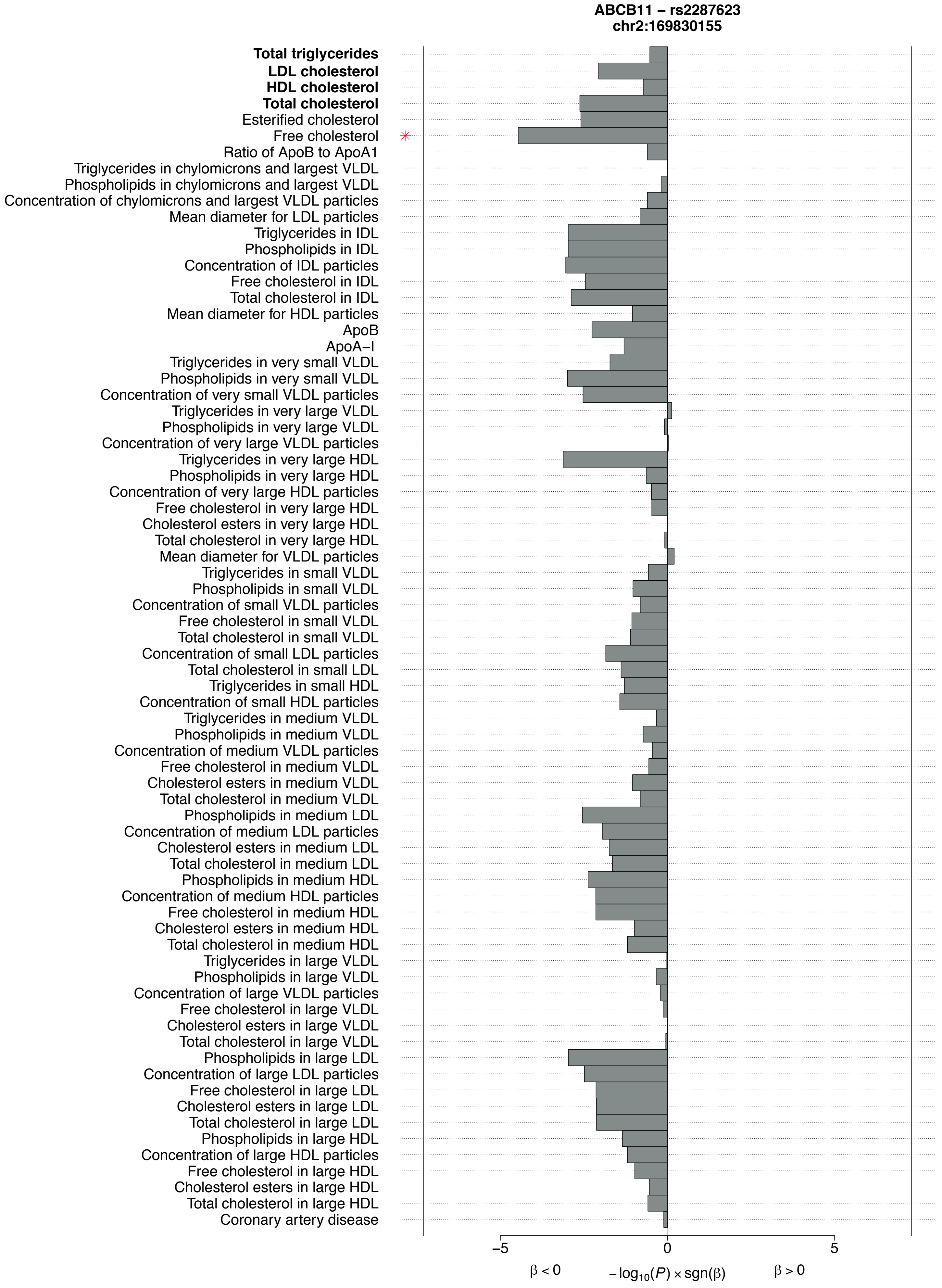
ABCB11 – rs2287623
chr2:169830155
Total triglycerides
LDL cholesterol
HDL cholesterol
Total cholesterol
Esterified cholesterol
Free cholesterol
Ratio of ApoB to ApoA1
Triglycerides in chylomicrons and largest VLDL
Phospholipids in chylomicrons and largest VLDL
Concentration of chylomicrons and largest VLDL particles
Mean diameter for LDL particles
Triglycerides in IDL
Phospholipids in IDL
Concentration of IDL particles
Free cholesterol in IDL
Total cholesterol in IDL
Mean diameter for HDL particles
ApoB
ApoA−I
Triglycerides in very small VLDL
Phospholipids in very small VLDL
Concentration of very small VLDL particles
Triglycerides in very large VLDL
Phospholipids in very large VLDL
Concentration of very large VLDL particles
Triglycerides in very large HDL
Phospholipids in very large HDL
Concentration of very large HDL particles
Free cholesterol in very large HDL
Cholesterol esters in very large HDL
Total cholesterol in very large HDL
Mean diameter for VLDL particles
Triglycerides in small VLDL
Phospholipids in small VLDL
Concentration of small VLDL particles
Free cholesterol in small VLDL
Total cholesterol in small VLDL
Concentration of small LDL particles
Total cholesterol in small LDL
Triglycerides in small HDL
Concentration of small HDL particles
Triglycerides in medium VLDL
Phospholipids in medium VLDL
Concentration of medium VLDL particles
Free cholesterol in medium VLDL
Cholesterol esters in medium VLDL
Total cholesterol in medium VLDL
Phospholipids in medium LDL
Concentration of medium LDL particles
Cholesterol esters in medium LDL
Total cholesterol in medium LDL
Phospholipids in medium HDL
Concentration of medium HDL particles
Free cholesterol in medium HDL
Cholesterol esters in medium HDL
Total cholesterol in medium HDL
Triglycerides in large VLDL
Phospholipids in large VLDL
Concentration of large VLDL particles
Free cholesterol in large VLDL
Cholesterol esters in large VLDL
Total cholesterol in large VLDL
Phospholipids in large LDL
Concentration of large LDL particles
Free cholesterol in large LDL
Cholesterol esters in large LDL
Total cholesterol in large LDL
Phospholipids in large HDL
Concentration of large HDL particles
Free cholesterol in large HDL
Cholesterol esters in large HDL
Total cholesterol in large HDL
Coronary artery disease
−5
0
5
β < 0
− log10(P) × sgn(β)
β > 0

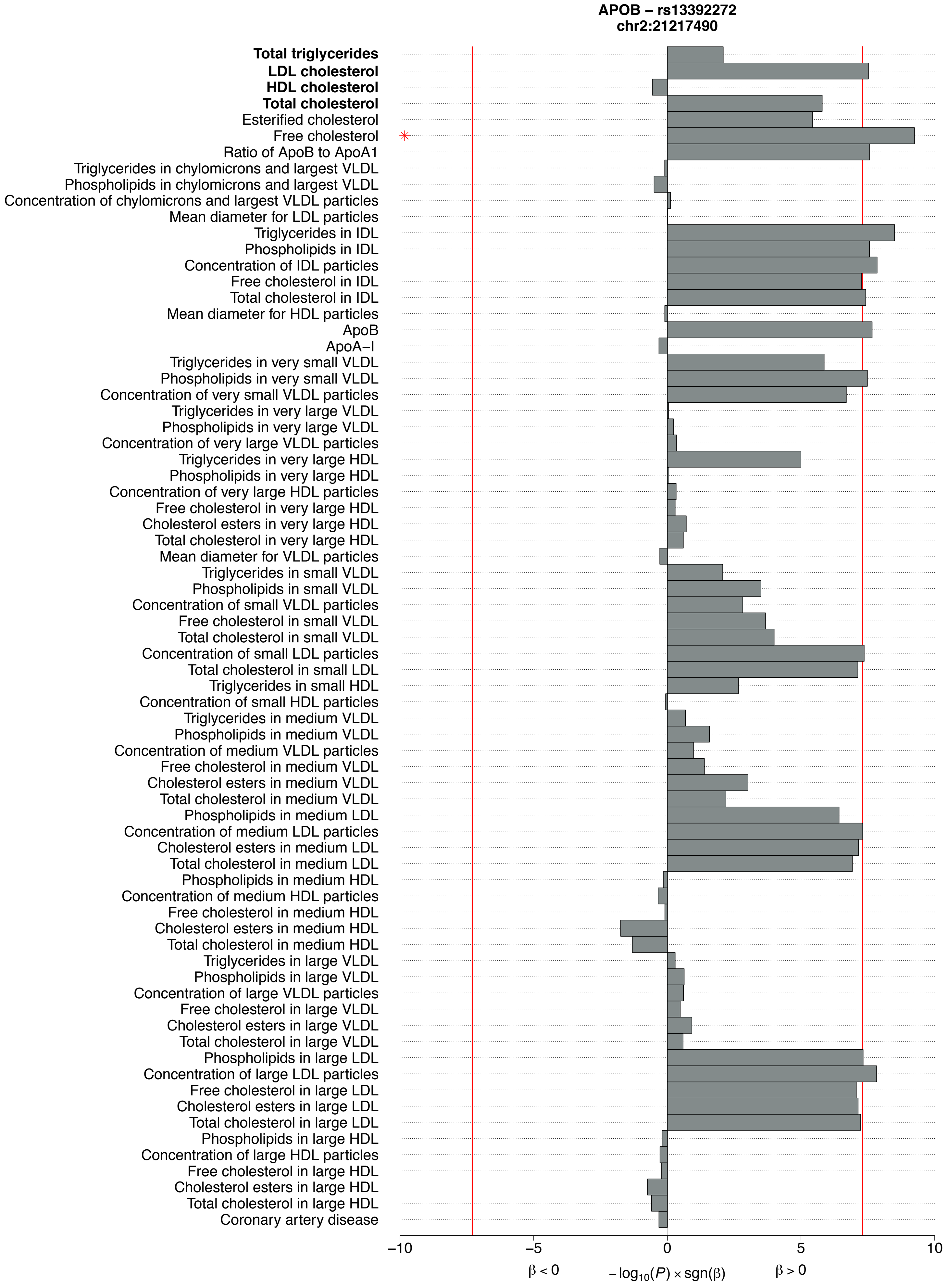
APOB – rs13392272
chr2:21217490
Total triglycerides
LDL cholesterol
HDL cholesterol
Total cholesterol
Esterified cholesterol
Free cholesterol
Ratio of ApoB to ApoA1
Triglycerides in chylomicrons and largest VLDL
Phospholipids in chylomicrons and largest VLDL
Concentration of chylomicrons and largest VLDL particles
Mean diameter for LDL particles
Triglycerides in IDL
Phospholipids in IDL
Concentration of IDL particles
Free cholesterol in IDL
Total cholesterol in IDL
Mean diameter for HDL particles
ApoB
ApoA–I
Triglycerides in very small VLDL
Phospholipids in very small VLDL
Concentration of very small VLDL particles
Triglycerides in very large VLDL
Phospholipids in very large VLDL
Concentration of very large VLDL particles
Triglycerides in very large HDL
Phospholipids in very large HDL
Concentration of very large HDL particles
Free cholesterol in very large HDL
Cholesterol esters in very large HDL
Total cholesterol in very large HDL
Mean diameter for VLDL particles
Triglycerides in small VLDL
Phospholipids in small VLDL
Concentration of small VLDL particles
Free cholesterol in small VLDL
Total cholesterol in small VLDL
Concentration of small LDL particles
Total cholesterol in small LDL
Triglycerides in small HDL
Concentration of small HDL particles
Triglycerides in medium VLDL
Phospholipids in medium VLDL
Concentration of medium VLDL particles
Free cholesterol in medium VLDL
Cholesterol esters in medium VLDL
Total cholesterol in medium VLDL
Phospholipids in medium LDL
Concentration of medium LDL particles
Cholesterol esters in medium LDL
Total cholesterol in medium LDL
Phospholipids in medium HDL
Concentration of medium HDL particles
Free cholesterol in medium HDL
Cholesterol esters in medium HDL
Total cholesterol in medium HDL
Triglycerides in large VLDL
Phospholipids in large VLDL
Concentration of large VLDL particles
Free cholesterol in large VLDL
Cholesterol esters in large VLDL
Total cholesterol in large VLDL
Phospholipids in large LDL
Concentration of large LDL particles
Free cholesterol in large LDL
Cholesterol esters in large LDL
Total cholesterol in large LDL
Phospholipids in large HDL
Concentration of large HDL particles
Free cholesterol in large HDL
Cholesterol esters in large HDL
Total cholesterol in large HDL
Coronary artery disease
−10
−5
0
5
10
β < 0
− log10(P) × sgn(β)
β > 0

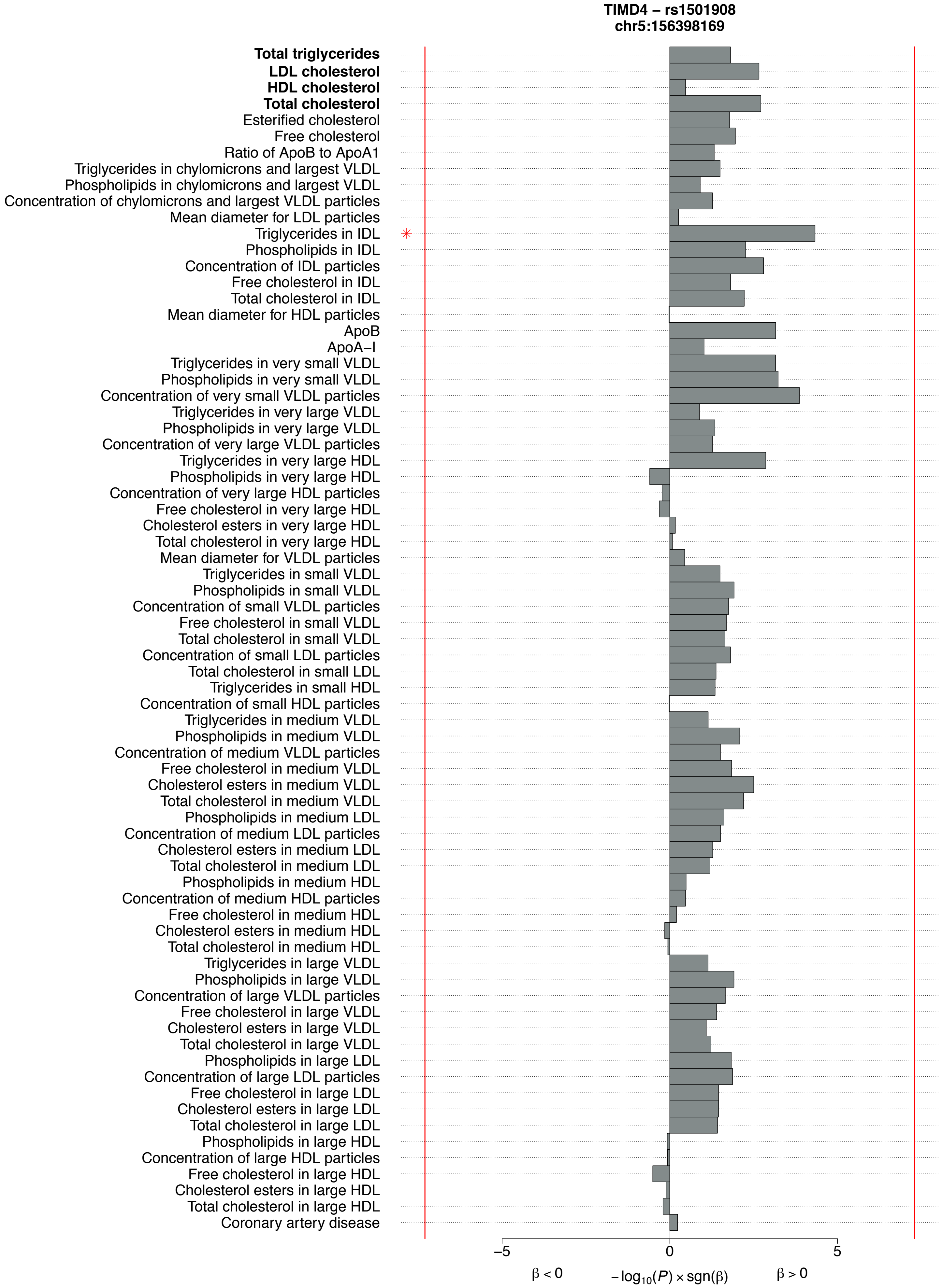
TIMD4 – rs1501908
chr5:156398169
Total triglycerides
LDL cholesterol
HDL cholesterol
Total cholesterol
Esterified cholesterol
Free cholesterol
Ratio of ApoB to ApoA1
Triglycerides in chylomicrons and largest VLDL
Phospholipids in chylomicrons and largest VLDL
Concentration of chylomicrons and largest VLDL particles
Mean diameter for LDL particles
Triglycerides in IDL
Phospholipids in IDL
Concentration of IDL particles
Free cholesterol in IDL
Total cholesterol in IDL
Mean diameter for HDL particles
ApoB
ApoA–I
Triglycerides in very small VLDL
Phospholipids in very small VLDL
Concentration of very small VLDL particles
Triglycerides in very large VLDL
Phospholipids in very large VLDL
Concentration of very large VLDL particles
Triglycerides in very large HDL
Phospholipids in very large HDL
Concentration of very large HDL particles
Free cholesterol in very large HDL
Cholesterol esters in very large HDL
Total cholesterol in very large HDL
Mean diameter for VLDL particles
Triglycerides in small VLDL
Phospholipids in small VLDL
Concentration of small VLDL particles
Free cholesterol in small VLDL
Total cholesterol in small VLDL
Concentration of small LDL particles
Total cholesterol in small LDL
Triglycerides in small HDL
Concentration of small HDL particles
Triglycerides in medium VLDL
Phospholipids in medium VLDL
Concentration of medium VLDL particles
Free cholesterol in medium VLDL
Cholesterol esters in medium VLDL
Total cholesterol in medium VLDL
Phospholipids in medium LDL
Concentration of medium LDL particles
Cholesterol esters in medium LDL
Total cholesterol in medium LDL
Phospholipids in medium HDL
Concentration of medium HDL particles
Free cholesterol in medium HDL
Cholesterol esters in medium HDL
Total cholesterol in medium HDL
Triglycerides in large VLDL
Phospholipids in large VLDL
Concentration of large VLDL particles
Free cholesterol in large VLDL
Cholesterol esters in large VLDL
Total cholesterol in large VLDL
Phospholipids in large LDL
Concentration of large LDL particles
Free cholesterol in large LDL
Cholesterol esters in large LDL
Total cholesterol in large LDL
Phospholipids in large HDL
Concentration of large HDL particles
Free cholesterol in large HDL
Cholesterol esters in large HDL
Total cholesterol in large HDL
Coronary artery disease
–5
0
5
β < 0
– log10(P) × sgn(β)
β > 0

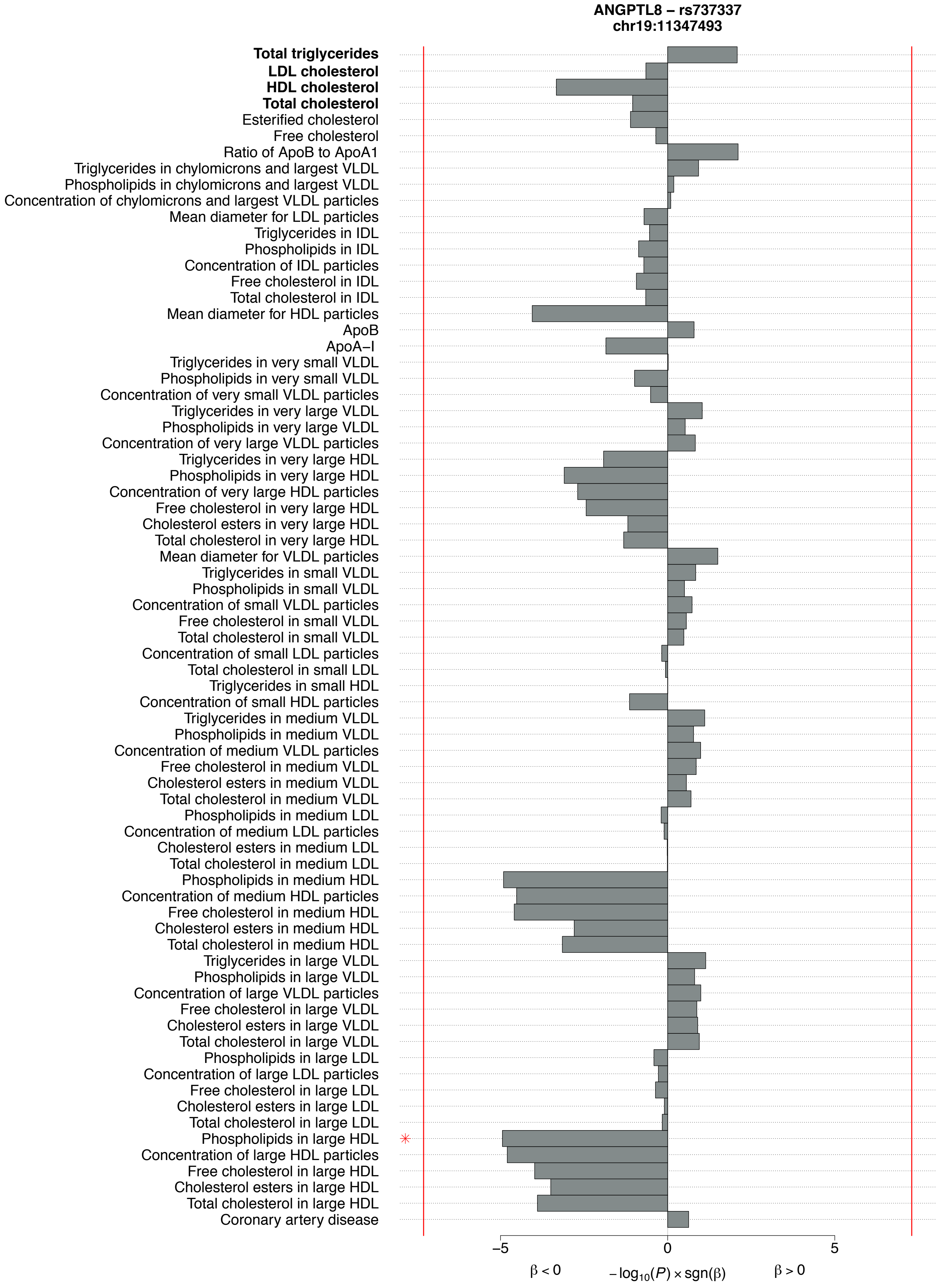

ANGPTL8 – rs737337
chr19:11347493
Total triglycerides
LDL cholesterol
HDL cholesterol
Total cholesterol
Esterified cholesterol
Free cholesterol
Ratio of ApoB to ApoA1
Triglycerides in chylomicrons and largest VLDL
Phospholipids in chylomicrons and largest VLDL
Concentration of chylomicrons and largest VLDL particles
Mean diameter for LDL particles
Triglycerides in IDL
Phospholipids in IDL
Concentration of IDL particles
Free cholesterol in IDL
Total cholesterol in IDL
Mean diameter for HDL particles
ApoB
ApoA−I
Triglycerides in very small VLDL
Phospholipids in very small VLDL
Concentration of very small VLDL particles
Triglycerides in very large VLDL
Phospholipids in very large VLDL
Concentration of very large VLDL particles
Triglycerides in very large HDL
Phospholipids in very large HDL
Concentration of very large HDL particles
Free cholesterol in very large HDL
Cholesterol esters in very large HDL
Total cholesterol in very large HDL
Mean diameter for VLDL particles
Triglycerides in small VLDL
Phospholipids in small VLDL
Concentration of small VLDL particles
Free cholesterol in small VLDL
Total cholesterol in small VLDL
Concentration of small LDL particles
Total cholesterol in small LDL
Triglycerides in small HDL
Concentration of small HDL particles
Triglycerides in medium VLDL
Phospholipids in medium VLDL
Concentration of medium VLDL particles
Free cholesterol in medium VLDL
Cholesterol esters in medium VLDL
Total cholesterol in medium VLDL
Phospholipids in medium LDL
Concentration of medium LDL particles
Cholesterol esters in medium LDL
Total cholesterol in medium LDL
Phospholipids in medium HDL
Concentration of medium HDL particles
Free cholesterol in medium HDL
Cholesterol esters in medium HDL
Total cholesterol in medium HDL
Triglycerides in large VLDL
Phospholipids in large VLDL
Concentration of large VLDL particles
Free cholesterol in large VLDL
Cholesterol esters in large VLDL
Total cholesterol in large VLDL
Phospholipids in large LDL
Concentration of large LDL particles
Free cholesterol in large LDL
Cholesterol esters in large LDL
Total cholesterol in large LDL
Phospholipids in large HDL
Concentration of large HDL particles
Free cholesterol in large HDL
Cholesterol esters in large HDL
Total cholesterol in large HDL
Coronary artery disease
−5
0
5
β < 0
−log10(P) × sgn(β)
β > 0

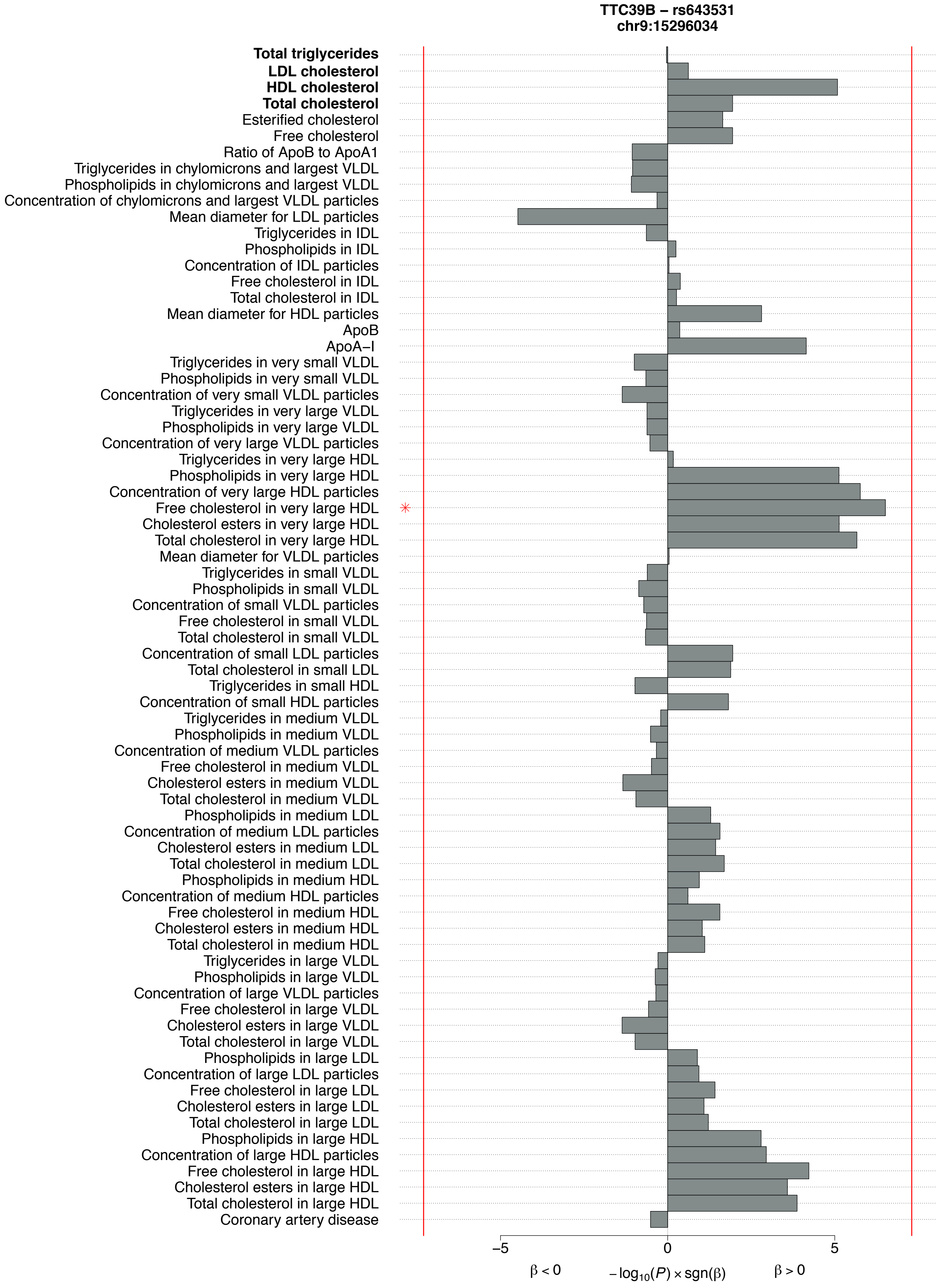

TTC39B – rs643531
chr9:15296034
Total triglycerides
LDL cholesterol
HDL cholesterol
Total cholesterol
Esterified cholesterol
Free cholesterol
Ratio of ApoB to ApoA1
Triglycerides in chylomicrons and largest VLDL
Phospholipids in chylomicrons and largest VLDL
Concentration of chylomicrons and largest VLDL particles
Mean diameter for LDL particles
Triglycerides in IDL
Phospholipids in IDL
Concentration of IDL particles
Free cholesterol in IDL
Total cholesterol in IDL
Mean diameter for HDL particles
ApoB
ApoA–I
Triglycerides in very small VLDL
Phospholipids in very small VLDL
Concentration of very small VLDL particles
Triglycerides in very large VLDL
Phospholipids in very large VLDL
Concentration of very large VLDL particles
Triglycerides in very large HDL
Phospholipids in very large HDL
Concentration of very large HDL particles
Free cholesterol in very large HDL
Cholesterol esters in very large HDL
Total cholesterol in very large HDL
Mean diameter for VLDL particles
Triglycerides in small VLDL
Phospholipids in small VLDL
Concentration of small VLDL particles
Free cholesterol in small VLDL
Total cholesterol in small VLDL
Concentration of small LDL particles
Total cholesterol in small LDL
Triglycerides in small HDL
Concentration of small HDL particles
Triglycerides in medium VLDL
Phospholipids in medium VLDL
Concentration of medium VLDL particles
Free cholesterol in medium VLDL
Cholesterol esters in medium VLDL
Total cholesterol in medium VLDL
Phospholipids in medium LDL
Concentration of medium LDL particles
Cholesterol esters in medium LDL
Total cholesterol in medium LDL
Phospholipids in medium HDL
Concentration of medium HDL particles
Free cholesterol in medium HDL
Cholesterol esters in medium HDL
Total cholesterol in medium HDL
Triglycerides in large VLDL
Phospholipids in large VLDL
Concentration of large VLDL particles
Free cholesterol in large VLDL
Cholesterol esters in large VLDL
Total cholesterol in large VLDL
Phospholipids in large LDL
Concentration of large LDL particles
Free cholesterol in large LDL
Cholesterol esters in large LDL
Total cholesterol in large LDL
Phospholipids in large HDL
Concentration of large HDL particles
Free cholesterol in large HDL
Cholesterol esters in large HDL
Total cholesterol in large HDL
Coronary artery disease
–5
0
5
β < 0
$-\log_{10}(P) \times \mathrm{sgn}(\beta)$
β > 0

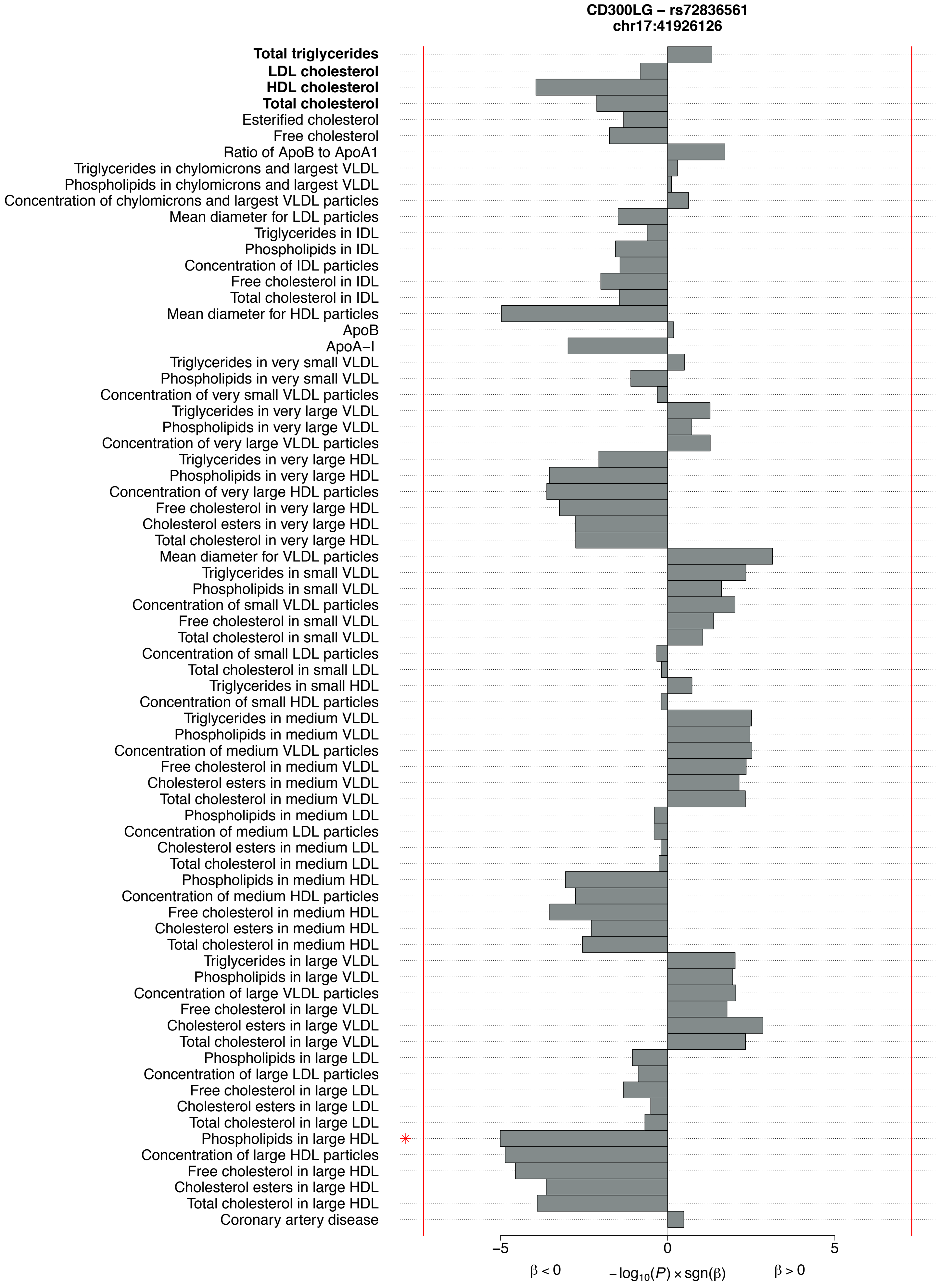
CD300LG – rs72836561
chr17:41926126
Total triglycerides
LDL cholesterol
HDL cholesterol
Total cholesterol
Esterified cholesterol
Free cholesterol
Ratio of ApoB to ApoA1
Triglycerides in chylomicrons and largest VLDL
Phospholipids in chylomicrons and largest VLDL
Concentration of chylomicrons and largest VLDL particles
Mean diameter for LDL particles
Triglycerides in IDL
Phospholipids in IDL
Concentration of IDL particles
Free cholesterol in IDL
Total cholesterol in IDL
Mean diameter for HDL particles
ApoB
ApoA–I
Triglycerides in very small VLDL
Phospholipids in very small VLDL
Concentration of very small VLDL particles
Triglycerides in very large VLDL
Phospholipids in very large VLDL
Concentration of very large VLDL particles
Triglycerides in very large HDL
Phospholipids in very large HDL
Concentration of very large HDL particles
Free cholesterol in very large HDL
Cholesterol esters in very large HDL
Total cholesterol in very large HDL
Mean diameter for VLDL particles
Triglycerides in small VLDL
Phospholipids in small VLDL
Concentration of small VLDL particles
Free cholesterol in small VLDL
Total cholesterol in small VLDL
Concentration of small LDL particles
Total cholesterol in small LDL
Triglycerides in small HDL
Concentration of small HDL particles
Triglycerides in medium VLDL
Phospholipids in medium VLDL
Concentration of medium VLDL particles
Free cholesterol in medium VLDL
Cholesterol esters in medium VLDL
Total cholesterol in medium VLDL
Phospholipids in medium LDL
Concentration of medium LDL particles
Cholesterol esters in medium LDL
Total cholesterol in medium LDL
Phospholipids in medium HDL
Concentration of medium HDL particles
Free cholesterol in medium HDL
Cholesterol esters in medium HDL
Total cholesterol in medium HDL
Triglycerides in large VLDL
Phospholipids in large VLDL
Concentration of large VLDL particles
Free cholesterol in large VLDL
Cholesterol esters in large VLDL
Total cholesterol in large VLDL
Phospholipids in large LDL
Concentration of large LDL particles
Free cholesterol in large LDL
Cholesterol esters in large LDL
Total cholesterol in large LDL
Phospholipids in large HDL
Concentration of large HDL particles
Free cholesterol in large HDL
Cholesterol esters in large HDL
Total cholesterol in large HDL
Coronary artery disease
−5
0
5
β < 0
$-\log_{10}(P) \times \mathrm{sgn}(\beta)$
β > 0

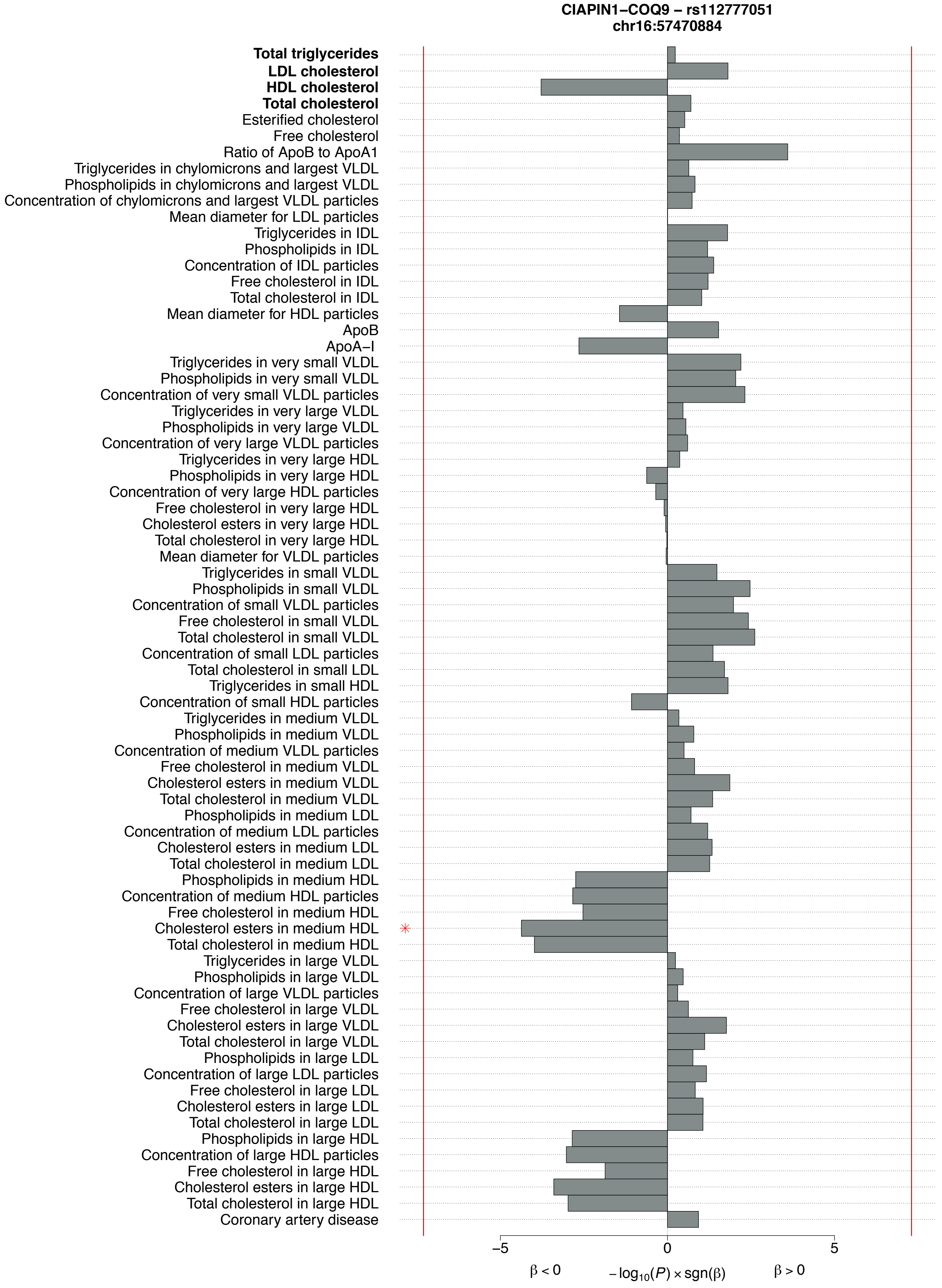
CIAPIN1–COQ9 – rs112777051
chr16:57470884
Total triglycerides
LDL cholesterol
HDL cholesterol
Total cholesterol
Esterified cholesterol
Free cholesterol
Ratio of ApoB to ApoA1
Triglycerides in chylomicrons and largest VLDL
Phospholipids in chylomicrons and largest VLDL
Concentration of chylomicrons and largest VLDL particles
Mean diameter for LDL particles
Triglycerides in IDL
Phospholipids in IDL
Concentration of IDL particles
Free cholesterol in IDL
Total cholesterol in IDL
Mean diameter for HDL particles
ApoB
ApoA–I
Triglycerides in very small VLDL
Phospholipids in very small VLDL
Concentration of very small VLDL particles
Triglycerides in very large VLDL
Phospholipids in very large VLDL
Concentration of very large VLDL particles
Triglycerides in very large HDL
Phospholipids in very large HDL
Concentration of very large HDL particles
Free cholesterol in very large HDL
Cholesterol esters in very large HDL
Total cholesterol in very large HDL
Mean diameter for VLDL particles
Triglycerides in small VLDL
Phospholipids in small VLDL
Concentration of small VLDL particles
Free cholesterol in small VLDL
Total cholesterol in small VLDL
Concentration of small LDL particles
Total cholesterol in small LDL
Triglycerides in small HDL
Concentration of small HDL particles
Triglycerides in medium VLDL
Phospholipids in medium VLDL
Concentration of medium VLDL particles
Free cholesterol in medium VLDL
Cholesterol esters in medium VLDL
Total cholesterol in medium VLDL
Phospholipids in medium LDL
Concentration of medium LDL particles
Cholesterol esters in medium LDL
Total cholesterol in medium LDL
Phospholipids in medium HDL
Concentration of medium HDL particles
Free cholesterol in medium HDL
Cholesterol esters in medium HDL
Total cholesterol in medium HDL
Triglycerides in large VLDL
Phospholipids in large VLDL
Concentration of large VLDL particles
Free cholesterol in large VLDL
Cholesterol esters in large VLDL
Total cholesterol in large VLDL
Phospholipids in large LDL
Concentration of large LDL particles
Free cholesterol in large LDL
Cholesterol esters in large LDL
Total cholesterol in large LDL
Phospholipids in large HDL
Concentration of large HDL particles
Free cholesterol in large HDL
Cholesterol esters in large HDL
Total cholesterol in large HDL
Coronary artery disease
–5
0
5
β < 0
–log10(P) × sgn(β)
β > 0

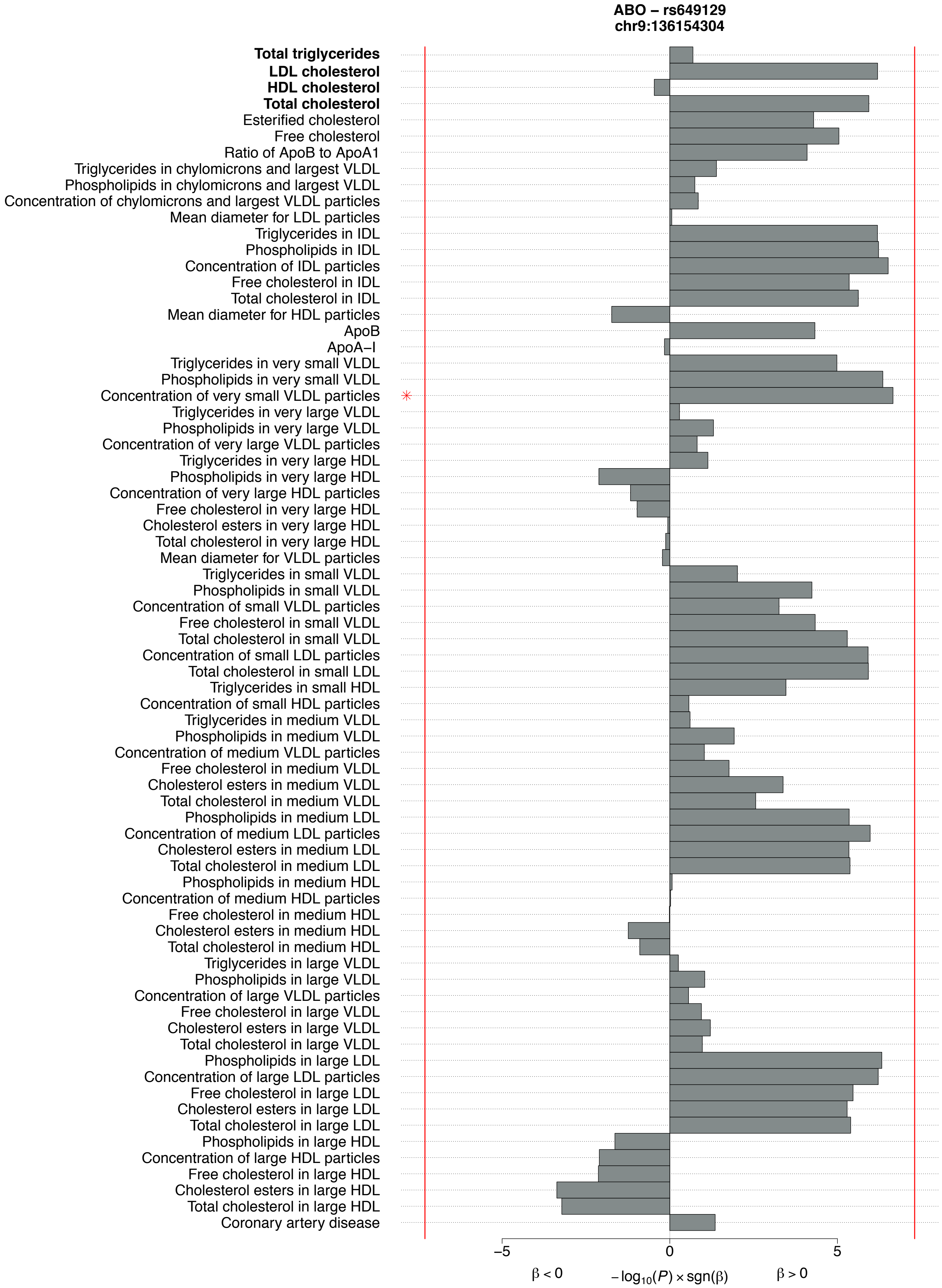
ABO – rs649129
chr9:136154304
Total triglycerides
LDL cholesterol
HDL cholesterol
Total cholesterol
Esterified cholesterol
Free cholesterol
Ratio of ApoB to ApoA1
Triglycerides in chylomicrons and largest VLDL
Phospholipids in chylomicrons and largest VLDL
Concentration of chylomicrons and largest VLDL particles
Mean diameter for LDL particles
Triglycerides in IDL
Phospholipids in IDL
Concentration of IDL particles
Free cholesterol in IDL
Total cholesterol in IDL
Mean diameter for HDL particles
ApoB
ApoA−I
Triglycerides in very small VLDL
Phospholipids in very small VLDL
Concentration of very small VLDL particles
Triglycerides in very large VLDL
Phospholipids in very large VLDL
Concentration of very large VLDL particles
Triglycerides in very large HDL
Phospholipids in very large HDL
Concentration of very large HDL particles
Free cholesterol in very large HDL
Cholesterol esters in very large HDL
Total cholesterol in very large HDL
Mean diameter for VLDL particles
Triglycerides in small VLDL
Phospholipids in small VLDL
Concentration of small VLDL particles
Free cholesterol in small VLDL
Total cholesterol in small VLDL
Concentration of small LDL particles
Total cholesterol in small LDL
Triglycerides in small HDL
Concentration of small HDL particles
Triglycerides in medium VLDL
Phospholipids in medium VLDL
Concentration of medium VLDL particles
Free cholesterol in medium VLDL
Cholesterol esters in medium VLDL
Total cholesterol in medium VLDL
Phospholipids in medium LDL
Concentration of medium LDL particles
Cholesterol esters in medium LDL
Total cholesterol in medium LDL
Phospholipids in medium HDL
Concentration of medium HDL particles
Free cholesterol in medium HDL
Cholesterol esters in medium HDL
Total cholesterol in medium HDL
Triglycerides in large VLDL
Phospholipids in large VLDL
Concentration of large VLDL particles
Free cholesterol in large VLDL
Cholesterol esters in large VLDL
Total cholesterol in large VLDL
Phospholipids in large LDL
Concentration of large LDL particles
Free cholesterol in large LDL
Cholesterol esters in large LDL
Total cholesterol in large LDL
Phospholipids in large HDL
Concentration of large HDL particles
Free cholesterol in large HDL
Cholesterol esters in large HDL
Total cholesterol in large HDL
Coronary artery disease
−5
0
5
β < 0
$-\log_{10}(P) \times \mathrm{sgn}(\beta)$
β > 0

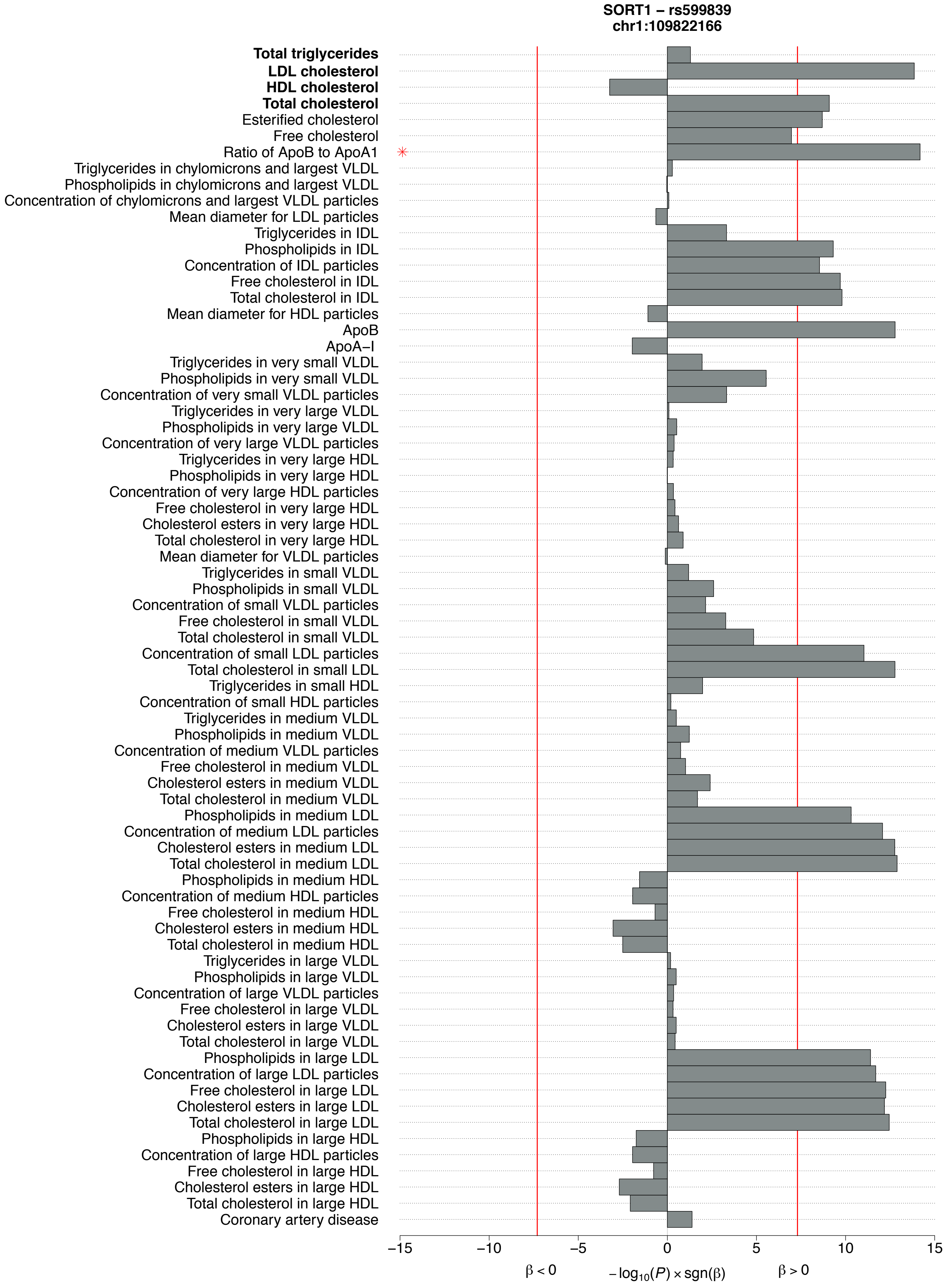
SORT1 – rs599839
chr1:109822166
Total triglycerides
LDL cholesterol
HDL cholesterol
Total cholesterol
Esterified cholesterol
Free cholesterol
Ratio of ApoB to ApoA1
Triglycerides in chylomicrons and largest VLDL
Phospholipids in chylomicrons and largest VLDL
Concentration of chylomicrons and largest VLDL particles
Mean diameter for LDL particles
Triglycerides in IDL
Phospholipids in IDL
Concentration of IDL particles
Free cholesterol in IDL
Total cholesterol in IDL
Mean diameter for HDL particles
ApoB
ApoA–I
Triglycerides in very small VLDL
Phospholipids in very small VLDL
Concentration of very small VLDL particles
Triglycerides in very large VLDL
Phospholipids in very large VLDL
Concentration of very large VLDL particles
Triglycerides in very large HDL
Phospholipids in very large HDL
Concentration of very large HDL particles
Free cholesterol in very large HDL
Cholesterol esters in very large HDL
Total cholesterol in very large HDL
Mean diameter for VLDL particles
Triglycerides in small VLDL
Phospholipids in small VLDL
Concentration of small VLDL particles
Free cholesterol in small VLDL
Total cholesterol in small VLDL
Concentration of small LDL particles
Total cholesterol in small LDL
Triglycerides in small HDL
Concentration of small HDL particles
Triglycerides in medium VLDL
Phospholipids in medium VLDL
Concentration of medium VLDL particles
Free cholesterol in medium VLDL
Cholesterol esters in medium VLDL
Total cholesterol in medium VLDL
Phospholipids in medium LDL
Concentration of medium LDL particles
Cholesterol esters in medium LDL
Total cholesterol in medium LDL
Phospholipids in medium HDL
Concentration of medium HDL particles
Free cholesterol in medium HDL
Cholesterol esters in medium HDL
Total cholesterol in medium HDL
Triglycerides in large VLDL
Phospholipids in large VLDL
Concentration of large VLDL particles
Free cholesterol in large VLDL
Cholesterol esters in large VLDL
Total cholesterol in large VLDL
Phospholipids in large LDL
Concentration of large LDL particles
Free cholesterol in large LDL
Cholesterol esters in large LDL
Total cholesterol in large LDL
Phospholipids in large HDL
Concentration of large HDL particles
Free cholesterol in large HDL
Cholesterol esters in large HDL
Total cholesterol in large HDL
Coronary artery disease
−15
−10
−5
0
5
10
15
β < 0
− log10(P) × sgn(β)
β > 0
